# RNA G-quadruplexes at upstream open reading frames cause DHX36- and DHX9-dependent translation of human mRNAs

**DOI:** 10.1186/s13059-018-1602-2

**Published:** 2018-12-27

**Authors:** Pierre Murat, Giovanni Marsico, Barbara Herdy, Avazeh Ghanbarian, Guillem Portella, Shankar Balasubramanian

**Affiliations:** 1 Department of Chemistry, University of Cambridge, Lensfield Road, Cambridge, CB2 1EW UK; 2 Cancer Research UK Cambridge Institute, University of Cambridge, Li Ka Shing Centre, Robinson Way, Cambridge, CB2 0RE UK; 3 School of Clinical Medicine, University of Cambridge, Cambridge, CB2 0SP UK

**Keywords:** Translation regulation, RNA secondary structures, G-quadruplexes, Helicases, Upstream open reading frames

## Abstract

**Background:**

RNA secondary structures in the 5′-untranslated regions (5′-UTR) of mRNAs are key to the post-transcriptional regulation of gene expression. While it is evident that non-canonical Hoogsteen-paired G-quadruplex (rG4) structures somehow contribute to the regulation of translation initiation, the nature and extent of human mRNAs that are regulated by rG4s is not known. Here, we provide new insights into a mechanism by which rG4 formation modulates translation.

**Results:**

Using transcriptome-wide ribosome profiling, we identify rG4-driven mRNAs in HeLa cells and reveal that rG4s in the 5′-UTRs of inefficiently translated mRNAs associate with high ribosome density and the translation of repressive upstream open reading frames (uORF). We demonstrate that depletion of the rG4-unwinding helicases DHX36 and DHX9 promotes translation of rG4-associated uORFs while reducing the translation of coding regions for transcripts that comprise proto-oncogenes, transcription factors and epigenetic regulators. Transcriptome-wide identification of DHX9 binding sites shows that reduced translation is mediated through direct physical interaction between the helicase and its rG4 substrate.

**Conclusion:**

This study identifies human mRNAs whose translation efficiency is modulated by the DHX36- and DHX9-dependent folding/unfolding of rG4s within their 5′-UTRs. We reveal a previously unknown mechanism for translation regulation in which unresolved rG4s within 5′-UTRs promote 80S ribosome formation on upstream start codons, causing inhibition of translation of the downstream main open reading frames. Our findings suggest that the interaction of helicases with rG4s could be targeted for future therapeutic intervention.

**Electronic supplementary material:**

The online version of this article (10.1186/s13059-018-1602-2) contains supplementary material, which is available to authorized users.

## Background

RNA secondary structures can modulate post-transcriptional regulation of gene expression. This can be achieved through controlling mRNA splicing, export, stability, localization and translation by either recruiting protein factors or by impeding scanning processes [1–4]. A scanning process is key to eukaryotic cap-dependent translation initiation [5] and involves the 43S preinitiation complex (PIC) scanning the 5′-UTR in the 3′-direction up to an initiation codon, where a complete 80S ribosome is formed and translation is initiated. To reach an initiation codon, helicases must either unwind secondary structures or remodel the PIC to help overcome impediments [6]. The best-characterized human helicases required for translation initiation comprise the DEAD-box helicases eukaryotic translation initiation factor 4A (eIF4A, also known as DDX2) [7, 8] and DDX3 [9]; and the DEAH-box helicases DHX29 [10] and DHX9 [11] (also known as RNA helicase A or RHA). Failure to process secondary structures during initiation can cause local PIC stalling and inefficient translation initiation. Since translation initiation is rate limiting for protein synthesis in eukaryotes [12–14], the processing of 5′-UTR structures by helicases directly controls the efficiency of mRNA translation. Transcriptome-wide characterization of human eIF4A [7, 8] and the yeast helicases, eIF4A/B and Ded1 [15, 16], revealed that impairing helicase activity affects the translation efficiency of mRNAs with structured 5′-UTRs. Thus, eukaryotic translation machinery can exploit RNA secondary structures in 5′-UTRs to discriminate between particular mRNA transcripts. Furthermore, stem-loop structures have been shown to improve the recognition of upstream initiator codons, in a suboptimal context, in vitro [17] and activate the translation of bacterial mRNAs [18]. Thus, RNA secondary structure folding can, in some contexts, increase the efficiency of initiation codon recognition and trigger the translation of alternative open reading frames.

Apart from stem-loops that comprise Watson-Crick-paired double-stranded RNA structures (dsRNA), non-canonical Hoogsteen-paired G-quadruplex (rG4) structures inserted or naturally occurring within 5′-UTRs have been shown to impair translation [19, 20]. Studies on a dozen rG4-containing 5′-UTRs inserted in front of reporter genes have demonstrated that rG4 forming sequences can inhibit protein synthesis in a manner that is dependent on the stability of the folded rG4 structure [21]. However, functional studies of rG4s in cells have been limited to the use of artificial reporter gene assays which do not necessarily recapitulate the effects of endogenous rG4-forming sequences in cellular transcripts [22, 23]. Such disparity might be explained by efficient helicase-mediated unfolding of rG4s in cellular mRNAs present at endogenous levels. Such observations highlight the need to study rG4s in their cellular context and call for a comprehensive analysis of the human transcriptome to identify functional rG4-forming sequences within the 5′-UTR of human mRNAs.

While helicases that unfold DNA G-quadruplexes have been well-studied [24], few helicases that bind and resolve rG4s have been identified. The best-characterized rG4 helicase is the DEAH-box helicases DHX36 (also known as RHAU and G4R1). DHX36 was discovered as the major source of DNA G-quadruplex resolving activity in HeLa cell lysates [25] and was later shown to bind rG4 with picomolar affinity and to process rG4 preferentially over DNA G-quadruplexes [26]. RNA immunoprecipitation experiments identified rG4s as enriched motifs in RNA bound by DHX36 in a cellular context [27]. DHX9, another DEAH-box helicase and a DHX36 paralog, was also reported to bind and resolve rG4s in vitro. In contrast to DHX36, DHX9 can process different secondary structures with a preference for RNA substrates [28]. Other helicases such as the DEAD-box helicases DDX21 [29], DDX5 and DDX17 [30], or the putative RNA helicase MOV10L1 [31] were also shown to be involved in rG4 binding and processing. These helicases have been proposed to contribute to the processing of miRNA and piRNA [30–32], and mRNA splicing [30] through rG4 recognition. However, the role of rG4-associated helicases in translational control remains generally unclear. The only example is that DHX36 associated with the Aven complex is required for optimal translation of two transcripts marked by rG4 motifs within their coding sequences (CDSs) and encoding for the oncogenic proteins MLL1 and MLL4 [33]. Impairing translation initiation, by small-molecule inhibition of eIF4A, has shown that r(GGC) repeats within 5′-UTRs that can fold into rG4 in vitro are involved in oncogenic processes [7] but a recent report has demonstrated that the r(GGC)_4_ motif folds into classical dsRNA in full-length mRNAs that require eIF4A for unwinding [34]. Hence, determining which helicases regulate translation of which mRNAs by unfolding rG4s in 5′-UTRs is essential to understand how rG4 folding affects translation initiation.

Here, we use transcriptome-wide approaches to define human transcripts whose translation is modulated by rG4s within their 5′-UTR, dissect the translational output of two rG4-unwinding helicases, and elucidate the mechanism linking rG4 folding and protein synthesis inhibition.

## Results

### Translation efficiency of mRNAs marked by G-quadruplexes within their 5′-UTRs

**Fig. 1:**
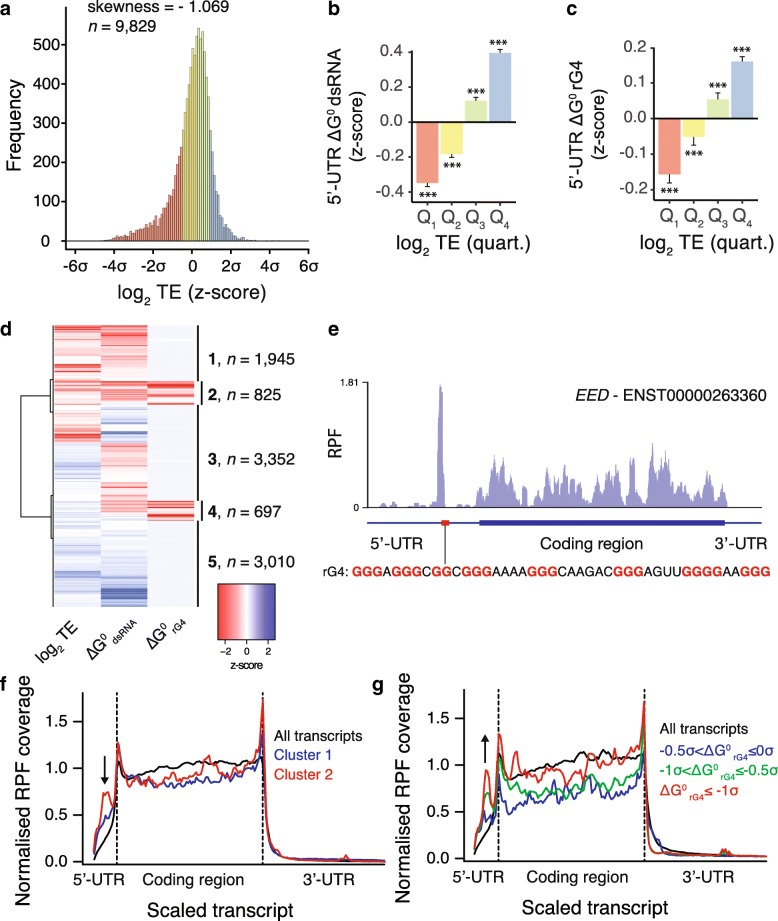
RNA G-quadruplexes within 5′-UTR alter ribosome distribution and impede translation of associated CDSs. **a** The transcriptome-wide distribution of translation efficiency of human mRNAs shows a skew towards inefficiently translated transcripts. **b** dsRNA and **c** rG4 associated predicted folding energies of 5′-UTRs of human transcripts binned according to their TE (first to fourth quartile of TE distribution). Folding energies are expressed as *z*-scores of the minimum free energies normalized by the length of the 5′-UTRs. **d** Hierarchical clustering analysis of human transcripts according to their TE and length-normalized dsRNA and rG4 predicted folding energies of 5′-UTRs. The heatmap reports the *z*-scores of the three variables. **e** Ribosome density within *EED* mRNA showing high ribosome occupancy in the 5′-UTR upstream of rG4-forming sequences (highlighted by a red box). Biophysical characterization of the *EED* rG4 motif is reported in Figure S2 in Additional file 1. **f** Ribosome distribution for transcripts of cluster 1 (blue) and cluster 2 (red), i.e., transcripts with low TE and associated with predicted stable dsRNA or rG4 structures respectively. **g** Ribosome distribution for transcripts of cluster 2 when binned for increasing predicted rG4 stability, − 0.5σ < ΔG^0^rG4 ≤ 0σ (blue line), − 1σ < ΔG^0^rG4 ≤ − 0.5σ (green line), and ΔG^0^rG4 ≤ −1σ (red line), where ΔG^0^rG4 is the *z*-score of 5′-UTR length-normalized rG4 predicted minimum free energy. Ribosome footprint coverage and transcript length are normalized; dotted lines indicates annotated translation start and stop sites and the arrows highlight the presence of RPFs in 5′-UTR. Data reported in **b** and **c** are means ± s.e.m.; *P* values were assessed using one-tailed Mann–Whitney nonparametric tests and represent statistical difference between the binned population and the rest of the population. *P < 0.05, ***P < 0.001

To identify transcripts regulated by rG4s in their 5′-UTR, we conducted transcriptome-wide ribosomal profiling [35, 36] of cycloheximide-treated (i.e., translation elongation inhibited) HeLa cells. Ribosome-profiling generated sequenced reads that exhibited a 28-nucleotide (nt) peak corresponding to 80S-ribosome protected fragments (RPFs) (Additional file 1: Figure S1a). We performed matched transcriptional mRNA sequencing (mRNA-seq) and defined the translation efficiency (TE) for each transcript by normalizing the ribosome footprint frequency to total transcript levels (transcript per millions (TPM) using signal in annotated CDSs). TE values from ribosome profiling showed good technical reproducibility (Spearman correlation = 0.935 across duplicates, Additional file 1: Figure S1b). The transcriptome-wide distribution of TE was skewed (skewness = − 1.069) towards inefficiently translated transcripts (Fig. 1a). To assess the contribution of secondary structures to translation efficiency we calculated the length-corrected minimum free energies of folded RNA secondary structures in 5′-UTRs for either dsRNA or rG4 structures (ΔG^0^_dsRNA_ and ΔG^0^_rG4_ respectively). Inefficiently translated mRNAs (first quartile of log_2_ TE distribution) were characterized by lower than average ΔG^0^_dsRNA_ and ΔG^0^_rG4_ values (i.e., more stable folded structures), while efficiently translated mRNAs (fourth quartile of log_2_ TE distribution) displayed higher than average ΔG^0^_dsRNA_ and ΔG^0^_rG4_ values (i.e., less stable structures, Fig. 1b, c and Additional file 1: Figure S1c). This analysis shows that structured 5′-UTRs is a general hallmark of inefficient translation. Interestingly, genes that were efficiently translated (fourth quartile of log_2_ TE distribution) are associated with the maintenance of basic cellular functions (i.e., housekeeping genes), while genes that were inefficiently translated (first quartile) are largely associated with cancer pathways (Additional file 1: Figure S1d) suggestive of specific mechanisms where RNA secondary structures maintain the TE of cancer genes at a low level.

To separate the contribution of rG4s from that of canonical dsRNA structures, in relation to the observed TE values, we devised a hierarchical clustering approach to identify groups of transcripts of similar TE and 5′-UTR folding energies. This approach identified clusters 1 and 2 (Fig. 1d), both exhibiting low TE and stable predicted secondary structures in their 5′-UTR. Cluster 1 includes transcripts with predicted dsRNA structures but no rG4 structures within their 5′-UTRs, whereas cluster 2 includes mRNAs with both dsRNA and rG4 structures predicted within their 5′-UTRs. It is noteworthy that the most stable rG4s are predicted within 5′-UTRs comprising less stable predicted dsRNA structures (Additional file 1: Figure S1e). Manual inspection of mRNAs from cluster 2, as exemplified by the transcript encoding for the Polycomb protein EED (Fig. 1e), revealed an accumulation of 80S ribosomes within 5′-UTRs. RPF coverage profiles of mRNAs from cluster 2 showed altered ribosome footprint distributions with high RPF density along 5′-UTRs and decreased in CDSs when compared to profiles obtained using all identified transcripts (Fig. 1f). RPF accumulation within 5′-UTRs was more pronounced for mRNAs of cluster 2 than mRNAs of cluster 1 indicating that rG4s has a relatively larger effect on RPF distribution than dsRNA (Fig. 1f). mRNAs from other clusters did not show any altered RPF distributions (Additional file 1: Figure S1f and g). Notably, RPF accumulation within the 5′-UTR of mRNAs of cluster 2 correlate with the predicted stability of rG4s (Fig. 1g and Additional file 1: Figure S1h) but not with the predicted stability of dsRNA (Additional file 1: Figure S1i) suggesting a direct role of rG4 folding on RPF distribution. These observations suggest that rG4s promote accumulation of 80S ribosomes within the 5′-UTR of mRNAs that are inefficiently translated. We confirmed that reads aligning to 5′-UTRs are true ribosome footprints, rather than non-ribosomal contaminants such as RNA regions that are protected by protein complexes or stable RNA secondary structure, by applying the fragment length organization similarity score (FLOSS) pipeline [37] (see Additional file 2).

### G-quadruplexes mark repressive upstream open reading frames

**Fig. 2:**
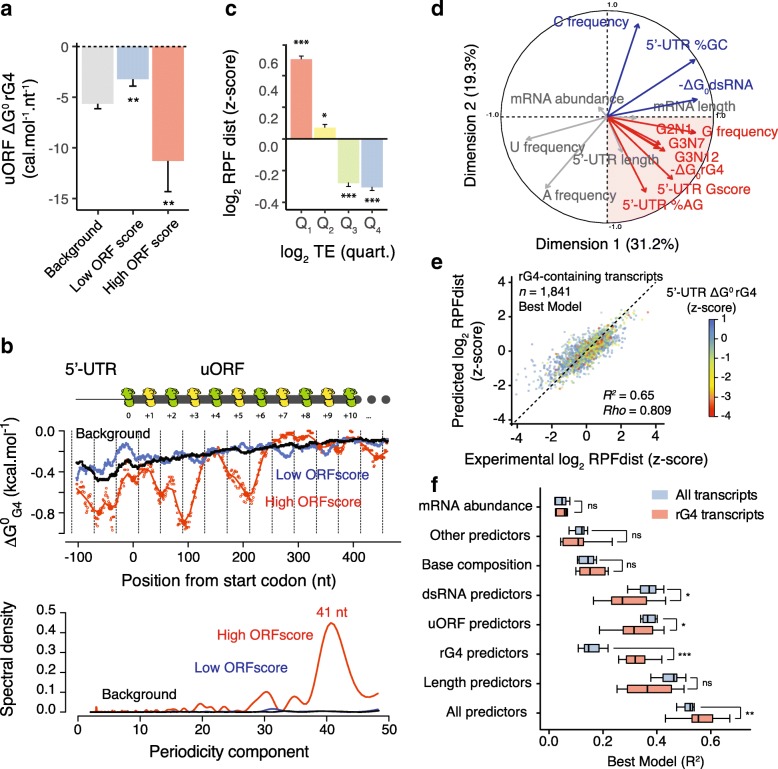
RNA G-quadruplexes are determinants of 5′-UTR translation. **a** Reduced predicted rG4 secondary structures folding energies are associated with translated uORF, i.e., high ORFscore uORFs (ORFscore ≥ 6). Folding energies are expressed as the minimum free energies normalized by the length of the uORFs. **b** rG4 structure potential downstream the start codons of translated uORFs (upper panel). Folding energies were calculated per position using a sliding window of 35 nt and the lines represent the average of the values over 10 nt. Filled points are the identified local minima for High ORF score uORFs. The dotted lines represent the size of 80S ribosomes (40 nt) phased downstream the start codon. The periodograms obtained from the position of rG4s within the uORFs are reported in the bottom panel and highlight a 41 nt periodicity in High ORF score uORFs. The cartoon above the plot depicts a “queue” of ribosomes stretching back to the uORF initiator codon. **c** Excess of RPF within 5′-UTR is associated with inefficient translation. The graph reports RPFdist, a proxy of 5′-UTR translation, expressed as *z*-score, of human mRNAs binned according to their TE (first to fourth quartile of TE distribution). **d** Principal component analysis of human transcripts using features describing mRNA abundance, 5′-UTR secondary structures, 5′-UTR and mRNA length, and 5′-UTR sequence composition statistics. The first two principal components, explaining ~ 50% of the variance, separate features describing rG4 structures (red quadrant) from features describing dsRNA structures (see also Figure S5a–b in Additional file 1 and Supplementary Information in Additional file 2). **e** A statistical model with as few as 32 predictors explains 65% of the RPFdist variation observed in the rG4-containing subset of transcripts (see also Supplementary Information in Additional file 2). **f** Performance of models selected using a subset of predictors on either all transcripts or the rG4-containing subset of transcripts. A model selected using rG4-based predictors only can account for 32.1 ± 8.4% of the observed RPFdist variance in the rG4-containing subset of transcripts making rG4-based predictors as informative as uORF-based predictors (32.0 ± 7.4%, mean ± s.d. over 10 resampling steps). Data in **a** and **c** are means ± s.e.m., *P* values were assessed using one-tailed Mann–Whitney nonparametric tests and compare the reported condition to either background or the rest of the population. Central black lines in (**f**) represent the medians and the other black lines represent quartile boundaries. *P* values were assessed using Kolmogorov–Smirnov nonparametric tests. ns: non significant, *P < 0.05, **P < 0.01, ***P < 0.001

Due to the presence of 80S ribosomes within rG4-containing 5′-UTRs, we then hypothesized that rG4 folding may decrease the translation efficiency of annotated CDSs by stimulating the translation of associated upstream open reading frames (uORFs). Because not all 80S ribosomes are actively engaged in translation, we assessed whether ribosomes within rG4-containing 5′-UTRs are stalled/poised or are actively translating using the ORFscore pipeline [38] (see the “Methods” section for more details). The ORFscore pipeline exploits the single-nucleotide resolution map of ribosome occupancy, determined by ribosome profiling, to quantify the accumulation of 80S ribosomes in the first frame (i.e., the reading frame) of an uORF and therefore defines actively translated regions within 5′-UTRS [38]. Our sequencing libraries show a good three-nucleotide periodicity (see Additional file 2) and we identified 7650 uORFs with 10× coverage, of which 1522 had a low score (0 ≤ ORFscore < 6) and 274 had a high score (ORFscore ≥ 6). Low ORFscores indicate low consistency between the distributions of RPFs with the frame of the uORFs reflecting their lack of coding potential. High ORFscores indicate strong phasing between RPF distributions and the reading frame of the uORFs, indicating actively translating ribosomes. uORFs with a high ORFscore comprised rG4s with higher predicted stability than for uORFs with a low ORFscore (Fig. 2a) suggesting that stable rG4s stimulate uORF translation. Interestingly, we observed that the position of predicted rG4 structures within high ORFscore uORFs are not random but enriched at specific positions downstream the initiator codons of translated uORFs (Fig. 2b). Deconvolution of the signal, i.e., position of rG4s relative to the upstream start codons, by the mean of a Fourier transform revealed a periodic enrichment every 41 nt with respect to the start site (Fig. 2b). The enrichment of rG4s downstream the initiator codons of translated uORFs suggests that rG4s may arrest or slow PIC scanning near upstream start codons, which could promote 80S ribosomes formation and translation. Knowing that a human ribosome is 250–300 Å in diameter and the average internucleotide distance in a ribosome/mRNA complex is 6.5 Å [39], a scanning ribosome is expected to cover 38–46 nt. Hence the observed periodic pattern of 41 nt, suggests that folding of rG4 structures within 5′-UTRs may pause scanning ribosomes inducing a “queue” of ribosomes stretching back to, and in front of, the initiator start codon. The prolonged presence of ribosomes over the sub-optimal upstream start codon may in turn increase engagement of the ribosome at this site leading to translation of the uORF. A similar mechanism has been recently proposed for the translation activation of an uORF within the 5′-UTR of the AZIN mRNA [40]. In our data, this effect appears to be specific to rG4s as there was no increase in GC-richness or periodic enrichment of predicted dsRNA structures either downstream or upstream of uORF start codons (Additional file 1: Figure S3a and b).

To assess how 5′-UTR translation affects the translation efficiency of mRNAs, we next considered RPFdist, which measures the loading of 80S ribosome in the 5′-UTR relative to the 80S loading in the downstream coding region (i.e., number of RPF reads in 5′-UTR/number of RFP reads in CDS). We observed a high RPFdist value for transcripts exhibiting low TE (Fig. 2c) consistent with an accumulation of 80S ribosomes within 5′-UTRs repressing translation of the downstream CDSs. Interestingly, we observed an enrichment of predicted rG4s in the 5′-UTR of transcripts with high RPFdist values (fourth quartile of log_2_ RPFdist distribution) (Additional file 1: Figure S3c–e). It is noteworthy that the concomitant presence of active uORFs, i.e., high ORFscore uORFs, and rG4s within the 5′-UTR of transcripts is associated with inefficient translation, whereas the presence of only one of these two features is associated with average translation efficiencies but with a significant redistribution of ribosomes towards the 5′-UTRs (Additional file 1: Figure S3f–h). Thus, rG4-forming sequences mark repressive upstream open reading frames. In contrast, 5′-UTRs of transcripts with high RPFdist values exhibited lower GC content and less stable predicted dsRNA structures than for 5′-UTR of transcripts with low or moderate RPFdist values (Additional file 1: Figure S3i and j) suggesting that dsRNA structures do not globally affect 5′-UTR translation.

### G-quadruplexes are determinants of 5′-UTR translation

We have shown that predicted rG4 structures mark 5′-UTR translation and inefficient CDS translation; however, rG4s did not fully account for the observed variation of TE and RPFdist values in our data. For example, TE values also correlate with the length of 5′-UTRs or the presence of known *cis*-regulatory elements, such as Cytosine Enriched Regulator of Translation (CERT) [41] and Pyrimidine-rich translation element (PRTE) [42] (Additional file 1: Figure S4a–c). To quantify the contribution of rG4s to 5′-UTR translation, we devised a quantitative model that integrates different mRNA features. We considered mRNA abundance, 5′-UTR secondary structures, 5′-UTR length, CDS length, base composition, the presence of AUG and non-AUG uORFs and known *cis*-regulatory elements, with the goal of predicting 5′-UTR ribosome occupancy, i.e., RPFdist measurement. A principal component analysis (see Supplementary Information in Additional file 2) based on a subset of these features showed that rG4-containing transcripts define a distinct group of transcripts (Fig. 2d). It is noteworthy, that dsRNA and rG4 features are separated by the second component suggesting that they contribute independently to ribosome distribution (Fig. 2d and Additional file 1: Figure S5a–b).

We then constructed regression models (see Supplementary Information in Additional file 2) to predict the RPFdist values of two sets of transcripts based on a list of potential predictors. The first set of transcripts included the 8024 transcripts expressed in HeLa cells with both 5′-UTRs (with a length ≥ 10 nt) and 3′-UTRs annotated, while the second group was the subset of 1841 transcripts displaying clear signature associated with rG4 structures within their 5′-UTRs (defined by the PCA with Dim.1 ≥ 0 and Dim.2 ≤ 0). Our analysis shows that the inclusion of rG4 structures substantially improved the prediction of ribosome distribution. The model trained on the global population of transcripts explained 56% of the variance in RPFdist (Additional file 1: Figure S5c), whereas the model trained on the rG4-containing 5′-UTR subset explained 65% of the variance in RPFdist (Fig. 2e). Moreover a model selected using only rG4-based predictors accounted for 32.1 ± 8.4% (mean ± s.d. over 10 resampling steps) of the RPFdist variance of the rG4-containing 5′-UTR subset (Additional file 1: Figure S5h–k). Our analysis showed that rG4-based predictors explained RPFdist variance as well as uORF-based predictors (32.0 ± 7.4%, mean ± s.d. over 10 resampling steps, Fig. 2f) demonstrating that rG4-forming sequences within 5′-UTRs are important determinants of 80S ribosome distribution within mRNAs.

### DHX9 and DHX36 are associated with polysomes and bind RNA G-quadruplexes

**Fig. 3:**
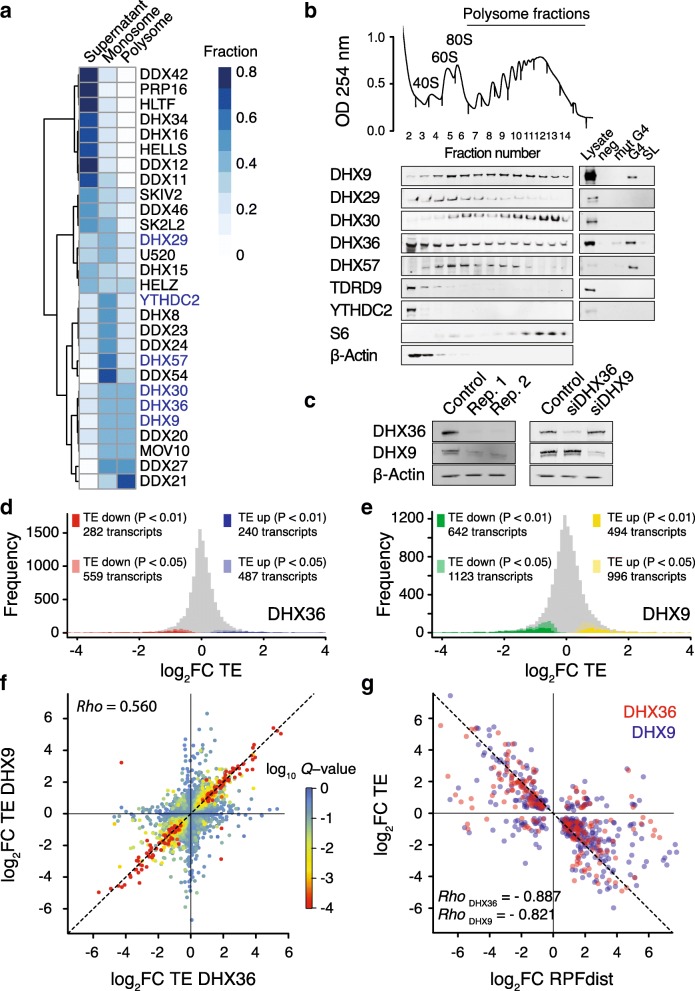
Polysome and ribosome profiling defines the role of DHX36 and DHX9 on translation. **a** Polysome profiling of HeLa cytoplasmic extract coupled with mass spectrometry allowed estimating the enrichment of different helicases in monosome or polysome fractions. Duplicates were used to quantitatively estimate the presence of a given helicase in each fraction. A hierarchical cluster analysis reporting the distribution of human helicases within mono/polysomes is shown. **b** Immunoblots of a polysome profile probing for members of the DEAH-box/RHA helicase family (left blot) confirmed the presence of DHX36 and DHX9 in both light and heavy polysomes. On the right is reported the result of affinity purifications using indicated biotinylated nucleic acids probes showing the presence of DHX9, DHX36, and DHX57 in rG4/ribonucleoprotein complexes. Lysate: 30 μg of total protein, Neg: empty beads, mut G4: mutated rG4-forming sequences, rG4: rG4-forming sequences, SL: stem-loop forming sequence. **c** Immunoblots of siRNA-treated cells probing DHX36 and DHX9 show that siRNA depletion of both helicases is reproducible and selective. Rep: replicate. Ribosome profiling allowed assessing change in TE upon depletion of **d** DHX36 and **e** DHX9. Are reported the frequency distributions of the ratio of TE in DHX36 (top) and DHX9 (bottom) depleted cells over TE in control (*n* = 3 replicates). **f** Comparison of fold changes of TE upon DHX36 and DHX9 depletion. *Q* values were calculated by combining *P* values using Fisher’s method. **g** Anticorrelation between fold changes of TE and fold changes of RPFdist upon depletion of DHX36 (red) or DHX9 (blue) for transcripts showing significant change in TE (*Q* value < 0.05)

To demonstrate a functional role for rG4 folding within 5′-UTR in translation regulation, we explored the contribution of helicases that bind and unwind rG4 structures towards controlling the translation efficiency of mRNAs with 5′-UTR rG4s. To identify helicases associated with polysomes, i.e., actively translated mRNAs, and bound to rG4s, we first performed polysome profiling coupled with proteomics mass spectrometry. HeLa cell lysates were fractionated into supernatant, monosomes (40S, 60S, and single 80S), and polysomes by sucrose density centrifugation (Additional file 1: Figure S6a). Isolated fractions were resolved by gel electrophoresis and high-molecular weight protein complexes were analyzed by mass spectrometry (Additional file 1: Figure S6b). Using function-based gene ontology, we observed enrichment of DEAD- and DExH-box helicases in the polysome fractions (Additional file 1: Figure S6c). A quantitative estimate (Fig. 3a) revealed that three out of the six paralogs of the DEAH-box/RHA family [43] of helicase co-sediment with the heavier polysomal fractions, namely DHX9, DHX30, and DHX36, suggesting a link between this helicase family and translation regulation. Both DHX9 and DHX36 have been previously reported to be associated with translating polyribosomes [11, 33], and we used immunoblotting (Fig. 3b) to compare the distribution of the helicases within the polysome fractions. We found that DHX9, DHX30, and DHX36 were present in both the heavy and light polysome fractions while DHX29 and DHX57 were enriched in the monosome fractions. The two other helicases from the family, TDRD9 and YTHDC2, were not associated with either mono or polysomes. We showed that an rG4 oligonucleotide probe enriched DHX9, DHX36, and DHX57, whereas both a mutated control that could not fold into an rG4 and a stem loop probe each showed no enrichment (Fig. 3b), showing rG4 binding specificity for these helicases. These observations suggest that DHX9 and DHX36 may be involved in the regulation of the translation of rG4-containing mRNAs.

### Translational landscapes of DHX36- and DHX9-deficient cells

To explore how DHX9, DHX36, and rG4s coordinate translation initiation, we performed ribosome profiling of HeLa cells depleted in either DHX36 or DHX9 by siRNAs that provided efficient and selective knockdowns (Fig. 3c). Depletion of both helicases did not affect cell proliferation or trigger eIF2 phosphorylation, a marker for global inhibition of translation (Additional file 1: Figure S7a–b), indicating that depletion of both helicases does not induce a general inhibition of protein synthesis. We assessed the TE of all HeLa transcripts, in triplicates for both knockdown conditions and for a control (pool of non-targeting siRNAs). To evaluate the TE change between the DHX36, DHX9, and control samples, we calculated the ratios TE_DHX36_/TE_control_ and TE_DHX9_/TE_control_. Changes in TE were reproducible across triplicates (Additional file 1: Figure S7c and f). We then contrasted genome-wide transcriptional and translational differences (Additional file 1: Figure S7c–f) and found that the change in TE upon helicase depletion correlated with the change in RPF signal (Spearman correlation = 0.762 and 0.744 for DHX36 and DHX9 knockdowns respectively) rather than change in total RNA signal (Spearman correlation = − 0.239 and − 0.299 for DHX36 and DHX9 knockdowns respectively), indicating a minimal impact of transcriptional variation in our measure of TE variation. We identified 1026 and 2119 transcripts that were significantly (*P* < 0.05) affected by the depletion of DHX36 and DXH9 respectively (Fig. 3d, e). Interestingly, the change in TE upon depletion of DHX36 also correlated with change in TE upon depletion of DHX9 (Pearson correlation = 0.560, Fig. 3f) suggesting that both helicases control a common subset of transcripts. We also found a strong anticorrelation between change in TE and in RPFdist (Fig. 3g, Pearson correlation = − 0.887 and − 0.821 for DHX36 and DHX9 depletion respectively), showing that the change in TE is accompanied with a significant shift in RPF distribution. Changes in RPFdist in both knockdown experiments were strongly correlated (Pearson correlation = 0.648, Additional file 1: Figure S7i) suggesting that both helicases share a common mechanism to regulate translation.

### G-quadruplexes mark the 5′-UTRs of mRNAs whose translation is DHX36- and DHX9-dependent

The mRNAs whose translation efficiency was affected by the helicase knockdowns and the mRNAs whose patterns of ribosomal occupancies were affected by the knockdowns showed significantly overlap (*P* < 0.01, Fisher exact test, Additional file 1: Figure S7j and k). We then defined two groups of mRNAs: a first group, named TE_down_ – RPFdist_up_ (characterized by diminished TE and increased RPFdist values upon the depletion of either helicases), and a second group, named TE_up_ – RPFdist_down_, (increased TE and decreased RPFdist values). The TE_down_ – RPFdist_up_ group included 282 transcripts, the TE_up_ – RPFdist_down_ group includes 195 transcripts, while the background list (whose TE is not affected by the depletion of either helicase) included 946 transcripts.

**Fig. 4:**
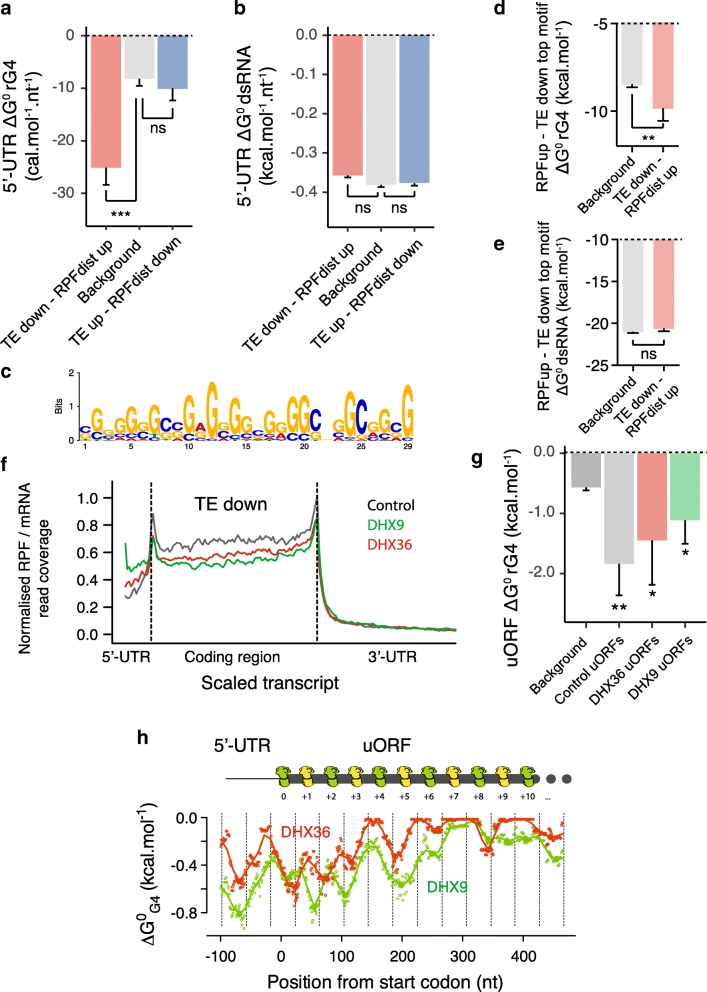
Translation is shifted towards rG4-containing 5′-UTRs in DHX36- and DHX9-depleted cells. rG4s mark the 5′-UTR of DHX36- and DHX9-dependent mRNAs. Are reported the 5′-UTR length-normalized (**a**) rG4 and (**b**) dsRNA secondary structures predicted folding energies of the TE_down_ – RPFdist_up_ and TE_up_ – RPFdisp_down_ groups compared to background transcripts (see main text for description of the different sets of transcripts). **c** Most enriched motif in the 5′-UTR of the TE_down_ – RPFdist_up_ group (*P* < 2.2 × 10^−16^, Fisher exact test). The same motif was found depleted in the 5′-UTR of TE_up_ – RPFdisp_down_ group (*P* = 2.4 × 10^−2^, Fisher exact test). **d** rG4 and **e** dsRNA secondary structures predicted folding energies of the TE_down_ – RPFdist_up_ top motif in the background or TE_down_ – RPFdist_up_ transcripts. Sequences corresponding to the identified motif ± 10 nt were considered to reflect the influence of 5′-UTR base composition on local predicted structures. **f** Ribosome distribution normalized by mRNA signal, describing local translation efficiency, of the TE_down_ group (903 transcripts with combined *Q* value ≤ 0.05) in control (black), DHX36 (red), and DHX9 (green) depleted cells. The plots show changes in 5′-UTR translation upon depletion of the helicases. Ribosome footprint, mRNA signal coverages, and transcript length are normalized; dotted lines indicate annotated translation start and stop sites. **g** Predicted rG4 structure folding energies of detected high ORFscore uORFs in control (gray) or DHX36 (red) and DHX9 (green) depleted cells. The background set (black) represents uORFs with negative ORFscore in control cells. Data are means ± s.e.m.; *P* values were assessed using one-tailed Mann–Whitney nonparametric tests. ns: non significant, *P < 0.05, **P < 0.01. **h** rG4 structure potential downstream the start codons of DHX36- and DHX9-dependent. The cartoon above the plot depicts a “queue” of ribosomes stretching back to the uORF initiator codon. Folding energies were calculated per position using a sliding window of 35 nt and the lines represent the average of the values over 10 nt. Filled points are the identified local minima. The dotted lines represent the size of 80S ribosomes (40 nt) phased downstream the start codon. The cartoon above the plot depicts a “queue” of ribosomes stretching back to the uORF initiator codon

Upon depletion of either helicase, we found the 5′-UTRs of affected transcripts in both the TE_down_ – RPFdist_up_ and TE_up_ – RPFdist_down_ subsets to be longer than the 5′-UTRs of the background subset (Additional file 1: Figure S7l). When normalized for 5′-UTR length, we found that lower than average ΔG^0^_rG4_ values (i.e., more stable folded structures) were found in the TE_down_ – RPFdist_up_ but not in the TE_up_ – RPFdist_down_ group (Fig. 4a). We also observed an enrichment of known rG4 forming motifs [44] of the form G_3+_N_1–7_G_3+_N_1–7_G_3+_N_1–7_G_3+_N_1–7_, where N is any base, in the 5′-UTR of the TE_down_ – RPFdist_up_ group (Additional file 1: Figure S7m). Interestingly, no differences in predicted dsRNA folding energies were observed for the two groups of mRNAs (Fig. 4b). These findings suggest that both 5′-UTR length and rG4 secondary structures make important contributions to changes in TE upon depletion of the helicases.

We used the MEME algorithm [45] to look for enrichment of motifs in each group (Additional file 1: Figure S8). While the TE_down_ – RPFdist_up_ group was characterized by the enrichment of short (12 to 30 nt) GC-rich motifs, the TE_up_ – RPFdist_down_ group was enriched in AT-rich motifs (Additional file 1: Figure S8a and b). The motifs enriched in the TE_down_ – RPFdist_up_ group were depleted in the TE_up_ – RPFdist_down_ group (Additional file 1: Figure S8c and d). The TE_down_ – RPFdist_up_ top motif (Fig. 4c) showed a skew in guanine composition and was found to overlap with known G-quadruplex forming sequences (Additional file 1: Figure S8e). Interestingly, when comparing the context of the sequences (considering a 10 nucleotides flanking region) matching this motif in the TE_down_ – RPFdist_up_ or the background group, we found that the sequences in the 5′-UTR of the TE_down_ – RPFdist_up_ group were characterized by a skew in purine, and more particularly in guanine, with no bias in GC content (Additional file 1: Figure S8f and h). This sequence context favors the formation of rG4 structures, which is confirmed by smaller values of rG4 folding energies, i.e., more stable, but similar values of dsRNA folding energies (Fig. 4d, e). Taken together, these results demonstrate that rG4 structures in 5′-UTRs are a significant determinant for DHX36- and DHX9-dependent translation.

### Depletion of DHX36 and DHX9 shifts translation to 5′-UTRs

RPF coverage profiles of mRNAs whose TE decreased upon the depletion of the DHX36 and DHX9 helicases revealed an increase of ribosome occupancy in 5′-UTRs coupled with a decrease in ribosome occupancy in coding regions (Fig. 4f). In contrast, an increase of RPF density within CDSs with no changes in RPF density within 5′-UTRs was observed when considering transcripts whose TE was increased upon the depletion of the helicases (Additional file 1: Figure S9a). Given that the 5′-UTRs of mRNAs from the TE_up_ group lack stable rG4s (Additional file 1: Figure S8), the increased TE upon helicase depletion is rG4-independent and must involve a different mechanism.

We identified uORFs that are activated upon depletion of the helicases using the ORFscore pipeline and detected 223 additional translated uORFs (uORFs with ORFscore ≥ 6) in cells depleted in either DHX36 or DHX9 as compared to the control cells (141 translated uORFs, Additional file 1: Figure S9b). The DHX36- and DHX9-dependent uORFs overlapped significantly (with 57 out of 223 new uORFs overlapping, *P* < 0.01, Fisher exact test) consistent with a shared mechanism for controlling 5′-UTR translation. The DHX36- and DXH9-dependent uORFs had more stable rG4s than uORFs from background (Fig. 4g). This latter result could not be explained by differences in uORF length (Additional file 1: Figure S9c) and was not observed when considering dsRNA structures (Additional file 1: Figure S9d). These observations suggest that rG4 folding within the 5′-UTR of DHX36- and DHX9-dependent mRNAs shifts translation to 5′-UTRs. It is noteworthy that predicted rG4 structures within DHX36- and DHX9-dependent uORFs were enriched at locations displaying a 42–44 nt pattern downstream the initiator codons (Fig. 4h and Additional file 1: Figure S9e) similar to translated uORFs in untreated samples (Fig. 2b). This result suggests that rG4 folding in the absence of the helicases induce ribosome queuing within the uORFs that stimulate translation initiation at the upstream initiator codons.

### DHX9 binds RNA-quadruplexes in human cells

**Fig. 5:**
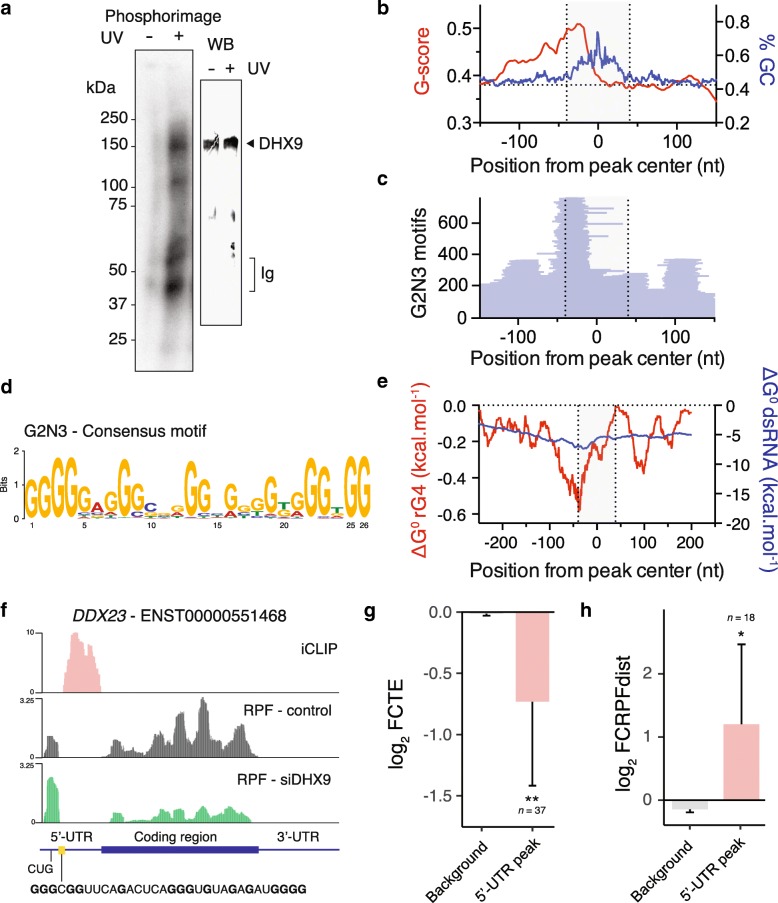
DHX9 binds downstream rG4 motifs in human cells. **a** Phosphorimage of SDS gel (left gel) resolving ^32^P-labeled RNAs crosslinked to DHX9. Immunoprecipitated samples prepared from HeLa cell lysates without UV light (254 nm) treatment are shown as a negative control. Immunoblot of the same membrane (right) probing DHX9 confirm the presence of DHX9 in the RNA-protein complex. **b** Gscore (red) and GC content (blue) of sequences around the center of iCLIP peaks. **c** Distribution of rG4 motifs, of the form G2 N3 (sequences of the form G_2+_N_1-3_G_2+_N_1-3_G_2+_N_1-3_G_2+_ where N is any base), around the center of iCLIP peaks. The gray area represents the median width of iCLIP peaks. **d** Consensus motif of rG4-forming sequences found upstream DHX9 iCLIP peaks. **e** rG4 and dsRNA predicted folding energies within 200 bases from the center of 5′-UTR DHX9 iCLIP peaks. Minimum folding energies were calculated using a sliding window of 35 nt. **f** Mapped DHX9 binding sites (red) and normalized ribosome density in control (black) and DHX9 depleted cells (green) within the *DDX23* transcript. Depletion of DHX9 leads to an increase and a decrease of RPF within the 5′-UTRs, upstream of DHX9 binding sites, and associated CDSs respectively. Biophysical characterization of the *DDX23* rG4 motif is reported in Figure S12 in Additional file 1. Fold changes in **g** TE and **h** RPFdist of transcripts directly bound by DHX9 in their 5′-UTR. Data are means ± s.e.m.; *P* values were assessed using two-sample Kolmogorov–Smirnov tests and compare the reported condition to background. *P < 0.05, **P < 0.01, ***P < 0.001

We used the Individual-nucleotide resolution UV crosslinking and immunoprecipitation (iCLIP) method [46] to map DHX9 binding sites in HeLa cells to determine whether DHX9 binds mRNAs directly via rG4s. The nature of the immunoprecipitated RNA-DHX9 complex was confirmed using controls either excluding UV cross-linking (Fig. 5a) or omitting the DHX9-specific antibody during the immunoprecipitation step (Additional file 1: Figure S10a). Deep sequencing of immunoprecipitated RNAs identified 2667 transcripts (encoding 2411 individual genes) bound by DHX9 with good reproducibility for two replicates (Additional file 1: Figure S10b, *Spearman* correlation = 0.88). We identified 5152 peaks with an average of ~ 2 peaks per transcript of which 7.6% and 89.3% were found in 5′-UTRs and 3′-UTRs respectively. The called peaks showed 97% overlap between replicates (Additional file 1: Figure S10c) supporting the robustness of our iCLIP protocol. After multimodal peak splitting, we identified 11,235 individual binding events characterized by discrete peaks of median width of 82 nt (Additional file 1: Figure S10d).

The DHX9 peaks were enriched in G, C and depleted in A, U residues (Additional file 1: Figure S11a) suggesting that DHX9 binds structured RNA sequences in a cellular context. Interestingly, a higher than baseline Gscore, indicative of guanine richness and rG4s [47], was observed upstream of the DHX9 binding sites (Fig. 5b) suggesting a role for rG4 motifs in DHX9 binding. Analysis of the position and frequency of discrete rG4 forming sequences, such as G2 N1: G_2+_N_1_G_2+_N_1_G_2+_N_1_G_2+_, G2 N3: G_2+_N_1-3_G_2+_N_1-3_G_2+_N_1-3_G_2+_, or G2 N5: G_2+_N_1-5_G_2+_N_1-5_G_2+_N_1-5_G_2+_, revealed enrichment of these rG4 motifs ~ 40 nt upstream of the DHX9 peaks center (Fig. 5c, Additional file 1: Figure S11b and c). Alignment of these motifs revealed G4-consensus motifs with defined G-tracts and short connecting loops (Fig. 5d, Additional file 1: Figure S11d and e) suggesting that DHX9 binds downstream rG4 motifs. This result was further supported by the enrichment of predicted stable rG4 motifs, proximal to all identified DHX9 peaks (Additional file 1: Figure S11f) and ~ 40 nt upstream DHX9 peaks within 5′-UTR (Fig. 5e). It is noteworthy that biochemical analyses of DEAH-box helicases have shown that efficient loading to rG4 substrates require 15 nucleotides downstream the rG4 structural motif and that the helicases translocate in the 3′ to 5′ direction [48, 49]. These observations are consistent with our finding that DHX9 binds 40 nt downstream of ~ 25 nt long rG4 motifs in a cellular environment.

To understand the relationship between DHX9 binding and the change in TE upon DHX9 depletion, we analyzed the ribosome distribution of transcripts displaying DHX9 iCLIP peaks within their 5′-UTR. Figure 5f displays the DHX9 iCLIP signal together with ribosome occupancy along the *DDX23* transcript showing RPF enrichment upstream of rG4-containing DHX9 binding sites within its 5′-UTR. Upon depletion of DHX9, ribosome occupancy within the 5′-UTR increased while it decreased within the downstream CDS. Overall, transcripts bound by DHX9 in their 5′-UTR were likewise characterized by a reduction in TE and an increase in RPFdist (Fig. 5g, h). We also found an enrichment of the TE_down_ – RPFdist_up_ top motif upstream of 5′-UTR DHX9 peaks (Additional file 1: Figure S11g and h). Taken together, these observations demonstrate the regulation of ribosome distribution and TE by DHX9 through direct binding to its rG4 substrate.

### DHX36- and DHX9-dependent transcripts

**Fig. 6:**
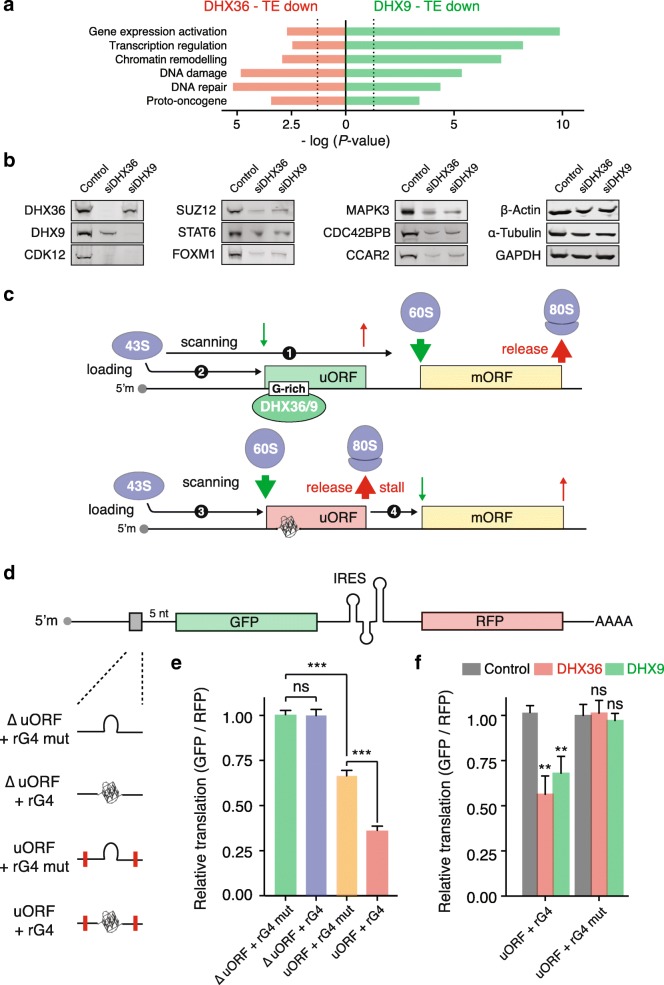
DHX36 and DHX9 mediate translation of selected cancer genes. **a** Gene ontology classification for genes, which TE decreases (*P* < 0.05) upon depletion of DHX36 (red) and DHX9 (green). **b** Immunoblots of lysates from HeLa cells depleted in DHX36 and DHX9 and probed as indicated. Immunoblots were performed 96 h after siRNA transfection. **c** Diagram showing a DHX36 / DHX9-dependent mechanism of translational control. (1) Scanning 43S PICs that translate unstructured 5′-UTRs or rG4-containning 5′-UTRs, that are maintained in their unfolded state by the DHX36 and DHX9 helicases, initiate translation at the main ORF (mORF). (2) A fraction of scanning PICs may initiate translation at upstream start codons, present within 5′-UTR in a suboptimal context, affecting the efficiency of the mORF translation. (3) In the absence of the rG4 processing helicases, rG4 motifs folding may slow down PIC scanning, thereby providing more time for the recognition of the upstream start codon and stimulating the translation of the upstream open reading frame (uORF). (4) 80S ribosomes may either dissociate from the mRNA after termination or stall during elongation or termination by the uORF-encoded attenuator peptide, preventing the translation of the mORF. **d** Schematic of bicistronic reporter genes containing within their 5′-UTRs either an rG4 motif (∆ uORF + rG4), a mutated rG4 (∆ uORF + rG4 mut), an rG4-containing uORF (uORF + rG4) or an rG4-mutated uORF (uORF + rG4 mut). **e** Relative translation of the different expression vectors showing that an rG4 enhances the repressive effect of a short uORF. **f** Effect of DHX36 (red) and DHX9 (green) depletion on the relative translation of reporter genes containing within their 5′-UTRs either an rG4-containing uORF or an rG4-mutated uORF as compared to control (non-targeting siRNAs, gray). Data represent the mean and s.d., *n* = 3 biological replicates. *P*-values were calculated using an unpaired student’s t-test. ns non-significant, **P < 0.01, ***P < 0.001. Representative flow cytometry profiles are reported in Additional file 1: Figure S15

Gene ontology classification for TE_down_ genes in DHX36 and DXH9 depleted samples (Fig. 6a) revealed a preponderance of factors involved in gene expression regulation, chromatin remodeling, and DNA damage/repair. Furthermore, we noted a significant enrichment of proto-oncogenes, such as MDM2, EGFR, or CCAR2. Genes dependent on both helicases highlighted a consistent enrichment of transcription factors (e.g., STAT6 or FOXM1), epigenetic regulators (e.g., SUZ12, MLL1, or MLL5), and kinases (e.g., MAPK3, MAP2K1, or CDC42BPB) in both TE_down_ and RPFdist_up_ groups (Additional file 1: Figure S13a and b). The individual RPF density plots illustrate recurrent patterns of altered ribosome distribution, whereas housekeeping genes (β-Actin, GAPDH, and α-Tubulin) show no changes in ribosome distribution profile upon depletion of the helicases (Additional file 1: Figure S13c). We confirmed the impact of DHX36 and DHX9 depletion on key target proteins (Fig. 6b and Additional file 1: Figure S13d), while controlling that the corresponding mRNAs were unaffected (Additional file 1: Figure S13e).

Given that the DHX36- and DHX9-dependent transcripts included many genes with a known role in cancer pathways, such as MAPK3/ERK1 or FOXM1 [50, 51], we considered the mutational and expression profiles of DHX36 and DHX9 in cancers to evaluate a potential contribution of both helicases in the oncogenic process. We did not find recurrent or frequent mutations associated with DHX36 and DHX9 in cancers (Additional file 1: Figure S14a), though we did find that human cancers show altered expression levels of both helicases. When comparing the expression levels of the helicases across normal tissue and tumors, recovered from the GENT database [52], we found that DHX36 displayed altered expression levels in eight and DHX9 in nine out of 15 types of cancers analyzed (Additional file 1: Figure S14b and c). When these helicases are dysregulated, they both showed higher expression levels in tumors than in normal tissue (Additional file 1: Figure S14d and e) suggesting a role for both helicases in stimulating cancer pathways.

### A DHX36/DHX9-dependent mechanism of translational control

Our data suggest that rG4 formation within the 5′-UTRs of a number of transcripts of biological interest impedes PIC scanning, thus promoting 60S ribosome recruitment and 80S ribosome formation upstream of canonical start codons. Upon DHX36- or DHX9-dependent activation, uORFs then thwart the translation of the downstream CDS (Fig. 6c).

To support our model, we constructed expression vectors in which the translation of a reporter GFP gene is driven by 5′-UTRs containing either an rG4, a short rG4-containing uORF, or a mutated rG4/alternative translation initiation site. It is noteworthy that the studied rG4 is the actual motif we found within the 5′-UTR of *DDX23* and characterized to be bound in cells by DHX9 and controlling its translation efficiency in a DHX36- and DHX9-dependent manner (Fig. 5f). We used bicistronic constructs in order to control for transcriptional variation and positioned the short uORF five bases upstream the downstream gene in order to minimize translation reinitiation at the downstream ORF. Given the uORF was positioned out-of-frame with respect to the downstream ORF, no ribosomes can translate the reporter gene ORF in the event that any ribosome translating the uORF reads through its stop codon (Fig. 6d).

Comparing the expression of the reporter gene driven by a 5′-UTR lacking an alternative translation initiation site and containing the rG4 motif to a similar construct in which the rG4 is mutated (Fig. 6e) shows that the presence of the rG4 has negligible impact on GFP expression. This result is consistent with a previous report demonstrating that rG4s within 5′-UTR do not act as a translational repressor when immediately upstream of a start codon [53]. Comparing the expression of the reporter gene driven by a 5′-UTR lacking an rG4 forming sequence and containing an alternative translation initiation site to a similar construct in which the alternative translation initiation has been mutated (Fig. 6e) shows that the presence of a short uORF moderately affects the expression of the reporter gene. Interestingly comparing the expression of the reporter gene driven by a 5′-UTR containing an alternative translation initiation site with a downstream rG4 to a similar construct in which only the rG4 is mutated (Fig. 6e) shows that an rG4 stimulates the repressive effect of the uORF. These observations support that an rG4 within an uORF stimulate translation initiation at the alternative translation initiation site and thwart the translation of the downstream CDS.

Finally, we assessed the contribution of the helicases to this mechanism by co-transfecting the expression vectors containing 5′-UTRs with either the rG4-containing uORF or the rG4-mutated uORF and siRNAs targeting DHX36 or DHX9 (Fig. 6f). This experiment revealed that depleting DHX36 or DHX9 decreases the expression of the reporter gene driven by an rG4-containing uORF but not of a similar reporter gene in which the rG4 has been mutated. This observation shows that the repressive effect of the uORF is DHX36- and DHX9-dependent, and only so when an rG4 is present.

## Discussion

Post-transcriptional regulation of gene expression allows a cell to orchestrate rapid changes in protein levels from steady state levels of mRNA. Cells have evolved *cis*-regulatory elements that are used to fine-tune the control of translation. Recent evidence supports that non-canonical secondary structures, such as rG4s, contribute to this mechanism by, for example, conferring eIF4A-dependent translation initiation [7] or by impeding ribosome translocation [54]. Herein, we have revealed a particular effect of rG4s on 5′-UTR translation. Specifically, our data suggests that rG4 structures in mRNAs can alter the distribution of ribosomes on mRNAs and that rG4s mark uORFs that upon active translation thwart the translation of the downstream CDS. This model is supported by a recent report suggesting that rG4 formation within G_4_C_2_ repeats from ALS/FTD C9ORF72 transcripts promote the translation of a short ORF using a CUG start codon located upstream of the repeats [55]. Our data suggest that rG4 folding within uORFs stimulate 5′-UTR translation by pausing translating ribosomes and inducing a queue of ribosomes stretching back to the uORF start codons. The prolonged presence of ribosomes over the uORF start codon may stimulate its translation, leading to decreased translation of the downstream CDS. A similar queuing model has been recently proposed for the regulation of yeast and human ORFs by modulating the recognition of weak start codons or by accumulating paused ribosomes within CDSs [40, 56]. Helicases may resolve stalled ribosomes by unfolding rG4s, hence “eliminating traffic jams” and stimulate the translation of the downstream CDS. We further tested this model, by studying the effect of the two DEAH-box helicases, DHX36 and DHX9, on translation efficiency. Our experiments suggest that unfolding of rG4 structures within the 5′-UTR is required to favor translation at canonical start codons.

We found that rG4 structures can stimulate the translation of short open ORFs controlled by AUG, and non-AUG, codons occurring within 5′-UTRs. Whether rG4-dependent uORFs are translated into stable peptides or N-terminal extensions, they could serve an important regulatory role. This is of particular interest because of growing evidence supporting a role of 5′-UTR translation on influencing human phenotypes and diseases. Indeed, besides the well-known role of uORFs in the integrated stress response pathway [57], polymorphic uORFs have been linked to gene expression variation [58] and 5′-UTR translation to tumor initiation [59]. That DHX36 and DHX9 are overexpressed in cancer tissues supports the proposed role of RNA helicases in tumor initiation, progression and maintenance [60]. This finding also supports that DHX36 and DHX9 may have potential to be exploited as cancer drug targets. Owing to the translational control redundancy of both helicases, they may not be essential in somatic cells for survival, but could become crucial in tumors in the absence of other rG4 processing factors establishing a “non-oncogene addiction” state [61].

Characterizing how two rG4-unwinding helicases modulate translation efficiency also supports that the rG4 structure rather than its nucleotide sequence repress translation. It was recently proposed that rG4s, in eukaryotic cells, are globally unfolded in their steady-state and that G-rich regions might impart function through transient folding or result from the stable association of rG4-binding proteins [57]. Our data shows that depletion of rG4 processing enzymes, that bind the rG4 structural motif in a cellular environment, causes changes in mRNA translation that are associated with rG4 structures demonstrating that rG4 folding can affect PIC scanning. Our work, that infers rG4 formation from ribosome pausing events and changes in translation, demonstrate that even transient rG4 formation can profoundly impact the translational landscape of human cells. In the same work [57], the authors show that deletion of DHX36 does not allow increasing rG4 formation to a level above the limit of detection of their footprinting assay. We have shown in this work that DHX36 and DHX9 depletion can dramatically affect the translation of transcripts of biological interest highlighting the need to improve or develop methods to probe RNA structures in vivo and better understand their impact on RNA biology.

## Conclusions

We have provided the first transcriptome-wide analysis on the impact of rG4s on human mRNA translation. We have demonstrated that the eukaryotic translation machinery can utilize rG4 folding to discriminate between particular mRNA transcripts. Our data support a previously unknown mechanism in which rG4 folding, controlled by the two DEAH-box helicases DHX36 and DHX9, impedes the scanning of the 43S preinitiation complex, promotes 80S ribosome formation within 5′-UTRs and consequently represses the translation of transcripts involved in key biological pathways. Because of the enrichment of transcripts with structured 5′-UTRs in cancer pathways and the overexpression of rG4-unwinding helicases in cancer tissues, our findings suggest rG4s associated helicases as new targets for therapeutic intervention.

## Methods

### Ribosome profiling

HeLa cells were cultured in Dulbecco’s modified Eagle Medium (Life Technology) supplemented with 10% FBS (Life Technology). For siRNA transfection, 5 × 10^6^ HeLa cells per 15 cm dish were cultured overnight and treated with 27 μL DharmaFECT 1 Transfection Reagent (Dharmacon) and 90 nM indicated siRNA in 1.8 mL OptiMEM media. Previously described DHX36 siRNA [62, 63] (GGGAACUGCGAAGAAGGUAUU, GUAAGGGAACUGCGAAGAA, and CGGCAUGUGGUACGCGAAA) and DHX9 siRNA (UAGAAUGGGUGGAGAAGAAUU and GGCUAUAUCCAUCGAAAUUUU) were transfected at equimolar concentrations. A pool of four non-targeting siRNAs (ON-TARGETplus Non-targeting Control Pool, Dharmacon) was used as control. The cells were then expended for 48 h. DHX36 and DHX9 downregulation was verified by immunoblot (see corresponding section). The cells were treated with cycloheximide (final concentration 0.1 mg mL^−1^) for 1 min prior lysis.

Total RNA and ribosome-protected fragments (RPFs) were isolated using the TruSeq Ribo Profile Mammalian Kit (Illumina) following the manufacturer’s instructions with some modifications. For RPF purification, lysates were treated with 5 U TruSeq Ribo Profile Nuclease per A_260_ of lysate. RPFs were isolated using Illustra MicroSpin S-400 HR Columns (GE Healthcare, 27-5140-01). rRNAs were removed using the Ribo-zero Magnetic Gold kit (Illumina, MRZG12324). After PAGE purification (15% urea-polyacrylamide gel to select fragments ~ 28–30 nt in length), end-repair, 3′-adapter ligation, reverse transcription (EpiScript reverse transcriptase) and cDNA circularisation (CircLigase, Epicentre), RPFs were amplified using 9 PCR cycles using Phusion polymerase (NEB). For parallel RNA-seq, total RNA was processed similarly, but excluding the nuclease digestion and S-400 columns purification steps. Before the library preparation steps, total RNA samples were heat-fragmented (94 °C for 25 min). PCR amplicons were purified on a 8% native polyacrylamide gel excising bands corresponding to ~ 70–80 nt and ~ 80–100 nt for RPF and total RNA samples respectively. Libraries were sequenced on a NextSeq 500 using 75-nt single-read sequencing runs.

### Sequencing alignment and mapping

RNA-seq libraries were trimmed using cutadapt [64]. The remaining reads were aligned to the GRCh38 human genome using the rsem-calculate-expression of RSEM [65]. For all analyses, we used the version 26 of the human genome transcript annotation from Gencode. For each experiment, the transcriptome was defined by considering transcripts with an expression above 1 TPM (Transcript Per Million) and isoform representation percentage above 5%. This yielded the following number of selected transcripts for each different experiment: 11,557 for untreated cells; 18,455 for control siRNA-treated cells; 18,115 for siDHX36-treated cells and 20,439 for siDHX9-treated cells. Twenty-two thousand nine hundred eleven transcripts representing the union transcriptome of all performed RNA-seq experiments were then considered for all following analyses, and the corresponding RPF libraries were then aligned to this reference transcriptome using RSEM. Only RPF reads in the size range 24–32 were considered for analysis. The libraries prepared in this study display a robust triplet periodicity suitable for analysis of translation (see Additional file 2). Isoform quantifications for both RNA-seq and RPF libraries were obtained in forms of estimated counts and TPM during the alignment procedure. Transcript coverage was calculated using RSEM command rsem-bam2wig and rsem-bam2readdepth, which both take into account multi-mapping reads and proportionally assign multiple assignments, a crucial step for processing short reads libraries [65]. The coverage plots were then used to calculate total signal at 5′-UTR, CDS, and 3′-UTR. Translation efficiency (TE) was calculated as the ratio of the total RPF signal (in TPM) over the coding region (CDS) divided by the total RNA signal (in TPM) over the same region: TE = RPF(CDS)/RNA(CDS). The 5′-UTR pattern of ribosomal occupancy, i.e., ribosome distribution, was defined as the ratio of the total RPF signal (in counts) over the 5′-UTR divided by the total RPF signal (in counts) over the associated CDS: RPFdist = RPF(5′-UTR)/RPF(CDS). Detailed results have been included in Additional file 3.

### Differential translation and ribosome distribution analysis

Change in translation efficiency upon depletion of the DHX9 and DHX36 helicases was defined as TE_siDHX36_/TE_control_ and TE_siDHX9_/TE_control_ respectively, where TE_control_ are values obtained from control siRNA-treated cells. To assess statistical relevance of changes in translation efficiency, RPF and total RNA signals (in TPM) were taken into Xtail [66], an *R* package designed for the identification of differentially translated genes in pairwise comparisons. Xtail estimates and accounts for biological variability in a statistical test based on the negative binomial distribution of the log2 fold changes in RPF and RNA signals. In our hands, Xtail was found to exhibit higher sensitivity as compared to other available pipelines, such as DESeq2 [67] or RiboDiff [68]. To assess co-changes in translation efficiency upon depletion of both helicases, *P* values (assessed using Xtail) were combined using Fisher’s method and referred to as *Q* values [69]. Change in ribosome distribution was defined as RPFdist_siDHX36_/RPFdist_control_ and RPFdist_siDHX9_/RPFdist_control_, where RPFdist_control_ are values obtained from control siRNA-treated cells. 5′-UTR and CDS RPF signal (in counts) were taken to DESeq2, as Xtail statistical model is tailored to translation efficiency estimation. *P* values and log_2_ fold changes estimated by each method were used for all the following analyses. Detailed results have been included in Additional file 4. The TE_down_ – RPFdist_up_ and TE_up_ – RPFdist_down_ groups were defined by selecting transcripts with decreased or increased TE (*Q* values ≤ 0.05) and increased or decreased RPFdist respectively.

### Meta-transcript profiles

Coverage files from estimated counts, generated by the alignment software RSEM (rsem-bam2readdepth command), were considered for the following analysis. To generate normalized profiles, total estimated counts, used for coverage normalization, were calculated for each library by summing up all transcript-wise estimated counts and dividing by 10^6^. The coverage profiles were calculated independently for 5′-UTR, CDS, and 3′-UTR by sampling the normalized profile signal in 15, 90, and 75 bins, respectively. The bin numbers were chosen to reflect the average length distribution of the corresponding regions in all identified HeLa transcripts (22,911 transcripts representing the union transcriptome). Normalized transcripts were averaged together in a vectored way to plot the coverage distribution. Total RNA and RPF signals were treated separately in the same way. To generate coverage plots, outlier values, exceeding the 99.9 percentile for total RNA and RPF signals, were removed and the signal was normalized by the area under the curve. For condition-averaged profiles, the profiles of individual libraries were first averaged and then processed similarly.

### Predictors of translation initiation and efficiency

5′-UTR sequences using annotation from the version 26 of the human transcriptome from Gencode were recovered. These sequences were used to calculate the quantitative parameters used to describe the different mRNA features discussed in this manuscript. A complete and comprehensive list of these features is reported in Additional file 2: Table S1. RNA secondary structures were predicted using the RNAfold 2.2.10 algorithm of the ViennaRNA package [70]. RNAfold computes the minimum free energy (MFE) of optimal secondary structures base on estimating base pairing probabilities. MFEs of dsRNA secondary structures (ΔG^0^_dsRNA_) were computed at 37 °C. MFEs of rG4 secondary structures (ΔG^0^_rG4_) were computed by subtracting MFEs obtained when considering rG4 formation into the structure prediction algorithm to the previous values (ΔG^0^_rG4_ = ΔG^0^_dsRNA_ − ΔG^0^_dsRNA + rG4_).

### Hierarchical clustering analysis

To find groups of similar transcripts or similar helicases, hierarchical clustering was performed using the *R* environment. For transcript clustering, log_2_ TE, 5′-UTR length normalized ΔG^0^_dsRNA_ and ΔG^0^_rG4_ values were used as *z*-scores. Different approaches were used to select the best clustering algorithm and to choose the optimal number of clusters. The reported analysis is using ‘canberra’ distance metrics and ‘ward.D2’ cluster method. Clusters were defined by identifying the five main groups of ΔG^0^_rG4_ values associated with different TE and ΔG^0^_dsRNA_. For helicases clustering, enrichments of each helicases in the three fractions (supernatant, monosomes, polysomes) were used as relative fractions (see the “NanoLC–MS/MS analysis of Polysomal fractions” section for calculation details). The reported analysis used “euclidean” distance metrics and “ward.D2” cluster method.

### Identification of uORFs and ORFscore calculation

Translated 5′-leader sequences in ribosome profiling data from HeLa and DHX36/DHX9-depleted HeLa cells were predicted using the ORFscore pipeline [38]. uORFs were defined as sequences in annotated 5′-UTRs (Gencode version 26) with start codons (AUG, CUG, UUG, and GUG) in frame with a stop codon (UAA, UAG, and UGA). An ORFscore was then calculated for each identified uORFs. To calculate the ORFscores, 28–29 nt RPF reads of each replicates were combined and counted at each position within the uORFs, excluding the first and last coding codons. Any uORFs without RPF reads in one of the replicate were excluded from the analysis. To avoid false positives due to little information about RPF positioning, uORFs with low read coverage (< 350 reads per kilobases) were excluded from the analysis. The ORFscore was then calculated as:

$$ \mathrm{ORFscore}={\log}_2\ \left(\left(\sum \limits_{i=1}^3\frac{{\left({F}_i-\overline{F}\ \right)}^2}{\overline{F}}\right)+1\right)\times \left\{\begin{array}{c}-1,\mathrm{if}\ \left({F}_1<{F}_2\right)\cup \left({F}_1<{F}_3\right)\ \\ {}1,\mathrm{otherwise}\end{array}\right. $$

where *F*_*n*_ is the number of reads in reading frame *n*, $\overline{F}$ is the total number of reads across all three frames divided by 3. Hence, the RPF distribution in each frame of a given uORF is compared to an equally sized uniform distribution using a modified chi-squared statistic. Negative and positive ORFscores are assigned when the distribution of RPFs is inconsistent or consistent, respectively, with the frame of a given uORF. An ORFscore threshold of 6 was used to call with confidence for uORFs that are translated since annotated CDS regions are characterized by higher than this threshold ORFscores [38]. Detailed results have been included in Additional file 5. Periodic enrichment of periodic rG4 motifs within uORFs was assessed using fast Fourier transforms of the signal comprising position of rG4s relative to the upstream start codons. Periodograms were generated with the spec.pgram function in R, and spectral densities were plotted to highlight the main periodicity component.

### Principal component analysis and statistical modeling

Principal component analysis (PCA) and the selection of predictive models were performed using the “factoextra” [71] and “caret” [72] package respectively in the *R* environment. 5′-UTR sequences using annotation from the version 26 of the human transcriptome from Gencode were recovered. These sequences were used to calculate the quantitative parameters used to describe the different mRNA features discussed in this manuscript. A complete and comprehensive list of these features is reported in Additional file 2: Table S1. A PCA was used to select the sets of transcripts displaying clear signature of rG4 structure in their 5′-UTR. We selected a subset of the mRNA features that best describe the variability in mRNA features in our dataset by assessing their variances in term of eigenvalues. The first two dimensions of the PCA reported in this manuscript described 50.5% of the variability of our dataset and used the different features reported in the Fig. 2d. We then used the PCA to select the subset of transcripts that is characterized by discrete rG4 predicted structures marking their 5′-UTR. This set of transcripts, referred to as “rG4-containing transcripts,” were defined by Dim.1 ≥ 0 and Dim.2 ≤ 0 and consisted of 1841 transcripts. We also considered all the transcripts expressed in HeLa cells, referred to as “all transcripts,” with fully annotated 5′-UTR (with a length ≥ 10 nt) and 3′-UTR. These sets represent 8024 transcripts. To identify which features explain the highest amount of variation in ribosome distribution (RPFdist), we used a 10-fold cross validation (CV) scheme to select a subset of features with good predictive power. To this end, each feature value was centered and scaled, i.e., calculated as *z*-score. To penalize for model complexity, predictor selection was performed using the LASSO (least absolute shrinkage and selection operator) procedure (“glmnet” method from the *R* “caret” package) optimizing penalty parameters over the internal cross validation steps. The procedure (using final alpha and lambda parameters of 0.05 and 1 respectively) selected 32 predictors with good predictive power. It is noteworthy that PRTE and TOP-like elements were discarded at this stage. We then assessed the correlations (using a threshold of |correlation| ≤ 0.85) and linear dependencies (using QR decomposition) in between the selected predictors and found that all selected predictors were independent. The selected list of predictors was then used to build regression models predicting ribosome distribution on both transcript data sets (“all transcripts” and “rG4-containing transcripts”). To this end, each feature was centered and scaled; both data sets were randomly portioned into four sets: 70% of the sets were used for training and the remaining 30% were equally portioned providing three testing sets. The training sets were used to select models using a gradient boosting approach (“gbm” model from the caret package). Models were optimized by tuning the gradient boosting parameters over a 10-fold cross validation scheme. The optimized parameters were the number of iterations, the complexity of the tree, the learning rate, and the minimum number of training set samples in a node to commence splitting. To assess the overall performance of the models, we then challenged them against the three test sets (see Additional file 1: Figure S5). Models explaining the highest amount of variation on both the training and test sets were selected. This procedure was used to select models predicting the ribosome distribution (RPFdist) over the two sets of transcripts while considering all predictors or only a subset of predictors according to their category. Seven categories of predictors were studied independently and each predictor was assigned to one of the category (see Additional file 2: Table S1) describing: mRNA abundance, sequence length, base composition statistics, dsRNA structures, rG4 structures, upstream open reading frames (uORF), or other features (such as known *cis*-regulatory elements of translation initiation). The performance of the best models selected for each set of transcripts and each subset of predictors is reported in Additional file 2: Table S1. To characterize the differences between models (generated using different categories of predictors) and quantify the contribution of each predictor category, we compared their resampling distributions. We generate resampling distributions (10 CV repeated 10 times) for each model, using the “resamples” function of the “caret” package. Since models were fit on the same versions of the training data, the differences between the resampling distributions reflect the differences between model performances rather that the correlations that may exist within-resamples. Differences between model performances were assessed using a non-parametric Kolmogorov-Smirnov test and are reported Fig. 2f.

### Polysome profiling

Polysome analysis was performed as previously described [73]. HeLa cells were grown in 15 cm dishes to 80% confluency. Cells were washed three times in cold PBS containing 100 μg mL^−1^ of cycloheximide and scraped off the plate using a rubber policeman and 1 mL of the same solution. Cells were centrifuged for 5 min at 1000 rpm and resuspended in 425 μL of hypotonic lysis buffer (5 mM Tris-HCl pH 7.5, 2.5 mM MgCl_2_, 1.5 mM KCl) supplemented with 25 μL 10% TritonX100, 25 μL 10% Sodiumdeoxicholate, 1 μL 1 M DTT and 5 μL RNAase inhibitor (40 U/μL, Promega). The supernatant was loaded onto a 10–50% sucrose gradient prepared in 20 mM HEPES-KOH pH 7.6, 100 mM KCl and 5 mM MgCl_2_ and was centrifuged in an SW40 rotor at 35,000 rpm for 2 h. Gradients were analyzed by piercing the tube with a Brandel tube piercer, passing 60% sucrose through the bottom of the tube, and monitoring the absorbance of the eluting material with an ISCO UA-6 UV detector. The different collected fractions were either analyzed by mass spectrometry or by immunoblotting.

### NanoLC–MS/MS analysis of polysomal fractions

Two biological replicates were performed. Thirty microliters of each polysome profiling fraction was loaded onto a 4–12% NuPAGE Bis-Tris gel that was run for 60 min at 180 V and stained with Coomassie Brilliant Blue. Band corresponding to proteins and protein complexes with molecular weight above 100 kDa were excised (see Additional file 1: Figure S6a) and analyzed by mass spectrometry as previously described [74]. A molecular weight cut-off was used in order to enrich for high molecular weight complexes and helicases, while minimizing noise and saturation due to highly abundant ribonucleoproteins and ribosomal proteins. Briefly, protein bands were excised, and following several washes, the gel pieces were subjected to a reduction step using 10 mM DTT in 100 mM ammonium bicarbonate (NH_4_HCO_3_) buffer for 45 min at 56 °C. Alkylation was performed using 55 mM iodoacetamide in 100 mM NH_4_HCO_3_ buffer for 30 min at room temperature in the dark. Digestion was performed using 10 μL of trypsin (10 mg/L in 50 mM NH_4_HCO_3_ buffer) overnight at 37 °C. Eluted peptides were recovered, and the gel pieces were subsequently washed in 2.5% formic acid in 80% aqueous acetonitrile for 30 min at 37 °C. The acid wash was combined with the original peptide eluate and dried. Samples were resuspended in 0.1% formic acid and analyzed directly by nano-LC-MS/MS.

Mass spectrometry (MS) was performed using an LTQ Velos-Orbitrap MS (Thermo Scientific) coupled to an Ultimate RSLCnano-LC system (Dionex). Optimal separation conditions resulting in maximal peptide coverage was achieved using an Acclaim PepMap 100 column (C18, 3 μm, 100 Å) (Dionex) with an internal diameter of 75 μm and capillary length of 25 cm. A flow rate of 300 nL/min was used with a solvent gradient of 5% B to 40% B in 55 min followed by increasing the gradient to 95% B over 10 min. Solvent A was 0.1% (*v/v*) formic acid, 5% DMSO in water, whereas the composition of solvent B was 80% (*v/v*) acetonitrile, 0.1% (*v/v*) formic acid, and 5% DMSO in water. The mass spectrometer was operated in positive ion mode using a Nth order double-play method to automatically switch between Orbitrap-MS and LTQ Velos-MS/MS acquisition. Survey full-scan MS spectra (from 400 to 1600 m/z) were acquired in the Orbitrap with resolution (R) 60,000 at 400 m/z (after accumulation to a target of 1000,000 charges in the LTQ). The method used allowed sequential isolation of the 20 most intense ions for fragmentation in the linear ion trap, depending on signal intensity, using CID at a target value of 3000 charges. For accurate mass measurements, the lock mass option was enabled in MS mode, and the 445.120025 ion was used for internal recalibration during the analysis. Target ions already selected for MS/MS were dynamically excluded for 30 s. General MS conditions were electrospray voltage, 1.50 kV with no sheath or auxiliary gas flow, an ion selection threshold of 1000 counts for MS/MS, an activation Q value of 0.25, activation time of 12 ms, capillary temperature of 200 °C, and an S-Lens RF level of 60% were also applied. Charge state screening was enabled, and precursors with unknown charge state or a charge state of 1 were excluded. Raw MS data files were processed using Proteome Discoverer v.1.4 (Thermo Scientific). Processed files were searched against the SwissProt human database using the Mascot search engine version 2.3.0. Searches were done with tryptic specificity allowing up to one miscleavage and a tolerance on mass measurement of 10 ppm in MS mode and 0.6 Da for MS/MS ions. Structure modifications allowed were oxidized methionine, and deamidation of asparagine and glutamine residues, which were searched as variable modifications. Using a reversed decoy database, false discovery rate (FDR) was less than 1%. Detailed results have been included in Additional file 6.

The presence of proteins in supernatant, monosome, and polysome fractions was qualitatively assessed by analyzing the number of unique peptides in each sample. Functional analysis of proteins was performed using the DAVID bioinformatics resources 6.8 [75] using all human proteins with molecular weight above 100 kDa as background. Quantitative enrichment of helicases in polysome fractions was assessed by calculating the ratio of number of unique peptides in each fraction (supernatant, monosome, or polysome) over the total number of unique peptides in all fractions. To avoid false positive, proteins with less than 10 unique peptides in all fractions were excluded from the analysis. Calculated relative fractions were used for the clustering analysis (see the “Hierarchical clustering analysis” section).

### Immunoblots and antibodies

Effects of siRNA transfection on DHX36 and DHX9 protein levels were assessed after 48 h. Down-stream effect of DHX36 and DHX9 depletion was tested after 96 h and two consecutive rounds of siRNA transfections. siRNA transfection was performed as described previously (see the “Ribosome profiling” section). Total cell lysates were prepared using Laemmli lysis buffer (92 mM Tris.HCl pH 6.8, 18% glycerol, 1.8% SDS, 0.02% Bromophenol Blue, 2% β-mercaptoethanol). Before lysis, cells were harvested by trypsinization, suspended in media with serum, and counted. After centrifugation (5 min at 1000 rpm), the cell pellets were resuspended in Laemmli lysis buffer at a concentration of 10^7^ cells per mL, heated at 95 °C for 5 min and sonicated. The equivalent of 10^5^ cells (10 μL) was loaded onto 4–12% NuPAGE Bis-Tris gels then transferred onto nitro-cellulose membranes using an iBlot 2 Gel Transfer Device (ThermoFisher Scientific). Membranes were blocked for 60 min in Odyssey Blocking Buffer (LI-COR Biosciences) and incubated overnight at 4 °C in solutions of primary antibodies in the blocking buffer. After washing with TBS supplemented with 0.1% Tween 20, membranes were incubated with secondary antibodies in the blocking buffer at room temperature for 60 min. IRDye secondary antibodies (LI-COR Biosciences) were used to detect protein bands on a Odyssey CLx Imaging System (LI-COR Biosciences).

Primary antibodies used in this study were DHX36 (Abcam ab70269), DHX9 (RNA Helicase A, Abcam ab26271), DHX29 (Abcam ab70745), DHX30 (Abcam ab85687), DHX57 (Abcam ab86784), TDRD9 (Abcam ab118427), YTHDC2 (Abcam ab176846), S6 ribosomal protein (Cell Signaling #2217), CDK12 (CRKRS, Abcam ab57311), SUZ12 (Abcam ab175187), STAT6 (Abcam ab32520), FOXM1 (Santa Cruz Biotechnology sc-376471), MAPK3 (ERK1, Abcam ab32537), CDC42BPB (Abcam ab61328), CCAR2 (KIAA1967, Abcam ab205526), β-Actin (ACTB, Cell Signalling #4970), α-Tubulin (TUBA1A, Cell Signalling #86298), and GAPDH (Sigma-Aldrich G8795).

### Affinity purification

Affinity purifications were performed as previously described with the following adjustments [76]. HeLa cells (4 × 10^6^ cells) were lysed in hypotonic lysis buffer as described and the protein concentration was determined with the BioRad protein assay (BiorRad) according to the manufacturer’s suggestions. One milligram of cytoplasmic extracts was precleared with Streptavidin MagneSphere® Paramagnetic Particles (Promega) in the RNA pull down buffer (20 mM Hepes pH 8, 100 mM NaCl, 20% glycerol, 0.2 mM EDTA, 1 mM DTT, 0.01% Nonidet-P40, 50 μg/mL yeast tRNA (Ambion), 160 U/mL RNasin). Prior to enrichments, 10 μM solutions of biotinylated oligonucleotides were annealed in 1× PBS supplemented with 2 M KCl by boiling for 5 min and slowly cooling to room temperature and kept at 4 °C until use. The nucleic acids probes were rG4 (Biotin-UGUGGGAGGGGCGGGUCUGGGUGC), mG4 (Biotin-UGUAGAAAGAGCAGAUCUAGAUGC), and SL (Biotin-ACAGGGCUCCGCGAUGGCGGAGCCCAA). Empty beads (B) were used as negative control. Biotinylated RNAs (5 nM) were bound to streptavidin beads and afterwards combined with precleared cytoplasmic extracts to perform affinity purifications for 4 h at 4 °C. The beads were washed with RNA pull down buffer three times and one time with 1× PBS. Interacting proteins were eluted by boiling in 50 μL 1× SDS Laemmli buffer. One half of the eluted protein complexes in Laemmli buffer were loaded onto a 4–12% NuPAGE Bis-Tris gel and the helicases of interest interrogated by immunoblotting with specific antibodies (see the “Immunoblots and antibodies” section).

### Motif identification and analysis

De novo motif discovery and analysis were performed using the Meme suite [45]. The 5′-UTR sequences of the TE_down_ – RPFdist_up_ and TE_up_ – RPFdist_down_ sets were collected for motif prediction. The Meme tool was run using both sequence datasets as primary sequences and the 5′-UTR sequences of all HeLa transcripts, as assessed by RNA-seq, were used as control sequences. A strand-specific three-order Markov model was used to correct for biased frequencies of all *k*-mers (*k* ≤ 3). Meme was run to identify enriched motifs of a maximum length of 30 nucleotides that occur any number of time in a given 5′-UTR sequence. The occurrence of the five more enriched motifs in the primary set of sequences, as compared to the control set, was called using FIMO with default parameters for strand-specific prediction correcting for biased frequencies of all *k*-mers (*k* ≤ 3). Enrichment of motifs was calculated by comparing the density of the motifs to the density of the same motif in the unchanged set, defined as transcripts with FC TE between − 0.1 and 0.1. Density of motifs is defined as the ratio of the number of motif occurrence and the total number of residues in a given set. *P* values for motif enrichment were calculated using a two-sided Fisher exact test. For base composition analysis and predicted secondary structure prediction, the sequences of the most enriched motif in the TE_down_ – RPFdist_up_ set were recovered together with a 10 nucleotides flanking region. *P* values for difference in base composition and stability of predicted structures were assessed using a Mann–Whitney test.

### iCLIP

Two biological replicates of DHX9 iCLIP were performed as previously described [46], with some modifications. For each experiment, 8 × 150 mm plates of HeLa cells were seeded to be at ~ 90% confluence during UV crosslinking. For crosslinking, the cells were washed with cold PBS and then the plates where irradiated on ice with 150 mJ/cm^2^ at 254 nm. The cells were scraped into PBS, pelleted by centrifugation at 1000 g for 5 min at 4 °C, and the pellets were resuspended in 500 μL lysis buffer (50 mM Tris-HCl, pH 7.4, 100 mM NaCl, 1% Igepal CA-360, 0.1% SDS, 0.5% sodium deoxycholate, 1/100 volume Protease Inhibitor Cocktail). At this stage, four lysates were combined to generate four samples for RNA digestion and immunoprecipitation. RNA digestion was performed using 4 unit of RNAse I (Life Technologies AM2295) per mL of lysate. Immunoprecipitation was performed using 100 μL of protein A-coated Dynabeads (Life Technologies 10002D) and a DHX9 antibody (Abcam ab26271) per IP. Beads were extensively washed with high-salt buffer (50 mM Tris-HCl, pH 7.4, 1 M NaCl, 1 mM EDTA, 1% Igepal CA-360, 0.1% SDS, 0.5% sodium deoxycholate). After 3′-end dephosphorylation, 3′ adaptor ligation, and ^32^P 5′-end labelling, a small aliquot (10% of the total volume) was saved for immunoblot analysis while the remaining samples were loaded onto a 4–12% NuPAGE Bis-Tris gel that was run for 60 min at 180 V. The RNA-protein complexes were transferred to a Protran BA85 Nitrocellulose Membrane using a Novex wet transfer apparatus for 2 h at 30 V. After transfer, the membrane was rinsed in PBS buffer and exposed to a Fuji film at − 20 °C overnight. Immunoblots were used to identify the DHX9-RNA complexes to be isolated from the membrane. At this stage, two nitrocellulose pieces were combined to generate the two biological replicates. Each sample was then incubated for 60 min at 50 °C in 200 μL PK/SDS buffer (100 mM Tris, pH 7.5, 50 mM NaCl, 1 mM EDTA, 0.2% SDS) supplemented with 10 μL proteinase K (Fisher Scientific YSJ-762-Q). RNA-protein complexes were recovered by Phenol:Chloroform extraction and ethanol precipitation. Reverse transcription was performed using Superscript III (Life Technologies 18080085) following the manufacturer’s instructions. Residual RNA was removed by alkaline hydrolysis. cDNA was size selected on a 6% polyacrylamide/7–8 M Urea/TBE gel run at 180 V for 40 min. Bands equivalent of 80–120 nt were excised from the gel. PCR amplification was performed using Accuprime Supermix I (Life Technologies 12342028) and 20 to 24 cycles to avoid the formation of secondary products. PCR products were purified on a 8% native polyacrylamide gel run at 200 V for 30 min. Amplicons of 140–170 nt were excised from the gel and recover by ethanol precipitation. PCR primers contained the Illumina P5 and P3 sequences together with degenerated barcodes for PCR duplicates removal. The iCLIP libraries were sequenced on a NextSeq 500 using 75-nt single-read sequencing runs.

Raw Illumina reads were processed as follows: Barcodes (NNNCGGANNN and NNNGGCANNN) were used for demultiplexing. PCR duplicates, i.e., reads having the same sequence and barcode, were removed using a customized Unix script. Remaining reads were aligned to the HeLa transcriptome (22,911 transcripts identified from RNA-seq experiments) using RSEM (rsem-calculate-expression command). Aligned reads were further processed for duplicates removal, i.e., reads aligning at the same location and having the same 10-mer barcode, leaving a single read per location. Transcript coverage was calculated using the rsem-bam2wig command, which takes into account and proportionally assigns multi-mapping reads. Bedgraph files were calculated (command bigWigToBedGraph from the UCSC utilities) and analyzed for peak calling using MACS2 [77] (command macs2 bdgpeakcall -l 40 -g 30 -c 5). Multi-modal peaks were redefined after combining reads from both duplicates and were splitted using PeakSplitter [78], the middle points from each peak were extracted for further analysis. Detailed results have been included in Additional file 7.

### Circular dichroism

CD spectroscopy experiments were conducted on a Chirascan Plus spectropolarimeter. Oligonucleotide solutions were prepared at a final concentration of 4 μM in 10 mM lithium cacodylate (pH 7.2) containing 1 mM EDTA supplemented with either 1 mM or 100 mM salt (where the salt is LiCl, NaCl, or KCl). Oligonucleotides were annealed at 95 °C for 3 min and store at 4 °C at least 12 h before analysis. Scans were performed over the range of 200–320 nm at 5 °C. Each trace was the result of the average of three scans taken with a step size of 1 nm, a time per point of 1 s and a bandwidth of 1 nm. A blank sample containing only buffer was treated in the same manner and subtracted from the collected data. The data were finally baseline corrected at 320 nm. Denaturation experiments were performed by heating the samples to 95 °C at a rate of 1 °C min^− 1^, with data collection every 1 °C. The CD signal at 265 nm was monitored and melting temperature (Tm) values were extracted as the half-maximum decrease in ellipticities.

### Reporter assay

The expression vectors used in this study were constructed from the pCru5-/GCCACC-mEGFP-IRES-mCherry vector (Addgene plasmid # 49226) which is a gift from Clifford Wang [79]. Sequences to control translation initiation were obtained by PCR amplification of synthetic oligonucleotides containing the sequences of interest (see sequences in Additional file 2: Table S2). Plasmids were obtained using standard molecular cloning procedures by inserting the sequences of interest between the XhoI and EcoRI restriction sites with restriction enzymes and T4 DNA ligase (New England BioLabs). Ligation reactions were transformed using TOP10 chemically competent cells (Thermofisher). Transformed bacteria were spread on LB agar plates containing ampicillin (100 μg/mL) and incubated at 37 °C overnight. Clones containing the desired plasmids were identified by Sanger sequencing and plasmids used in the transfection experiments were purified with a Monarch Plasmid Miniprep kit (New England BioLabs). Plasmid DNAs were transiently transfected into HeLa cells in 12-well plates using FuGENE HD (Promega) following the manufacturer instructions and 1 μg of DNA per well. Transfection was performed 48 h prior analysis. In the case of co-transfection with siRNAs, siRNAs and plasmid DNAs were transfected 72 h and 48 h prior analysis respectively. Expression of mEGFP and mCherry was analyzed by flow cytometry on a MACSQuant VYB cytometer. Relative translation is quantified as the ratio of expression of eGFP over mCherry for each event and the reported values are the averages of ratios.

### Statistics

Data were analyzed and statistics performed in Prism6 (GraphPad) and the *R* environment. Significant differences between two groups were noted by asterisks (*P < 0.05, **P < 0.01, ***P < 0.001). Replicates (*n*) in this study refer to biological replicates.

## Additional files

Additional file 1: **Figure S1.** Ribosome profiling of HeLa cells. **Figure S2.** Biophysical characterization of the rG4 motif found in the 5′-UTR of EED. **Figure S3.** Characterization of 5′-UTR translation in HeLa cells. **Figure S4.** Contribution of known cis-regulatory elements to translation efficiency. **Figure S5.** Principal component analysis (PCA) and statistical modelling of RPFdist variation. **Figure S6.** Polysome profiling allows assessing helicase enrichment in polysomes. **Figure S7.** Ribosome profiling defines the role of DHX36 and DHX9 in translation. **Figure S8.** Motifs discovery and analysis within the 5′-UTR of DHX9- and DHX36-dependent mRNAs. **Figure S9.** Characterization of DHX36- and DHX9-dependent uORFs. **Figure S10.** Reproducibility of the DHX9 iCLIP experiment. **Figure S11.** Characterization of DHX9 iCLIP peaks. **Figure S12.** Biophysical characterization of the rG4 motif found in the 5′-UTR of DDX23. **Figure S13.** DHX36- and DHX9-dependent transcripts. **Figure S14.** Mutation and expression profiles of DHX36 and DHX9 in cancer. **Figure S15.** rG4s stimulate the repressive effect of uORFs in a DHX36- and DHX9- dependent manner. (PDF 5852 kb)

Additional file 2: Supplementary Information. (PDF 1838 kb)

Additional file 3: Ribosome profiling and RNA-seq signals in count and TPM over the 5′-UTR and CDS of all transcripts expressed in HeLa cells. (XLS 17233 kb)

Additional file 4: Differential translation efficiency (TE) and ribosome distribution (RPFdist) values for all transcripts expressed in HeLa cells. (XLS 2802 kb)

Additional file 5: ORFscores for all putative uORF in non-treated, control, DHX36- and DHX9-depleted HeLa cells. (XLS 12540 kb)

Additional file 6: NanoLC–MS/MS analysis of polysomes profiling fractions. (XLS 3708 kb)

Additional file 7: Coordinate of all identified DHX9 iCLIP peaks. (XLS 1126 kb)

Additional file 8: Review history. (DOCX 47 kb)
