# Correction to: RNA G-quadruplexes at upstream open reading frames cause DHX36- and DHX9-dependent translation of human mRNAs

**DOI:** 10.1186/s13059-019-1623-5

**Published:** 2019-01-11

**Authors:** Pierre Murat, Giovanni Marsico, Barbara Herdy, Avazeh T. Ghanbarian, Guillem Portella, Shankar Balasubramanian

**Affiliations:** 1 Department of Chemistry, University of Cambridge, Lensfield Road, Cambridge, CB2 1EW UK; 2 Cancer Research UK Cambridge Institute, University of Cambridge, Li Ka Shing Centre, Robinson Way, Cambridge, CB2 0RE UK; 3 European Bioinformatics Institute (EMBL-EBI), Hinxton, Cambridge, CB10 1SD UK; 4 School of Clinical Medicine, University of Cambridge, Cambridge, CB2 0SP UK

**Correction to: Genome Biol**

**https://doi.org/10.1186/s13059-018-1602-2**

Following publication of the original article [1], the authors reported the following error in the name of the fourth author.

Incorrect author name: Avazeh Ghanbarian

Correct author name: Avazeh T. Ghanbarian

Avazeh T. Ghanbarian is affiliated with ‘European Bioinformatics Institute (EMBL-EBI), Hinxton, Cambridge, UK, CB10 1SD’.
