# Correction to: RNA G-quadruplexes at upstream open reading frames cause DHX36- and DHX9-dependent translation of human mRNAs

**DOI:** 10.1186/s13059-019-1737-9

**Published:** 2019-06-18

**Authors:** Pierre Murat, Giovanni Marsico, Barbara Herdy, Avazeh T. Ghanbarian, Guillem Portella, Shankar Balasubramanian

**Affiliations:** 1 Department of Chemistry, University of Cambridge, Lensfield Road, Cambridge, CB2 1EW UK; 2 Cancer Research UK Cambridge Institute, University of Cambridge, Li Ka Shing Centre, Robinson Way, Cambridge, CB2 0RE UK; 3 European Bioinformatics Institute (EMBL-EBI), Hinxton, Cambridge, CB10 1SD UK; 4 School of Clinical Medicine, University of Cambridge, Cambridge, CB2 0SP UK

**Correction to: Genome Biol (2018) 19:229**

**https://doi.org/10.1186/s13059-018-1602-2**

Following publication of the original article [1], the authors reported the following error in the name of the fourth author.

Incorrect author name: Avazeh Ghanbarian

Correct author name: Avazeh T. Ghanbarian

Avazeh T. Ghanbarian is currently affiliated with ‘European Bioinformatics Institute (EMBL-EBI), Hinxton, Cambridge, UK, CB10 1SD’

Further to this correction, the authors have reported a typographical error in the equation under the section *Predictors of translation initiation and efficiency.*

“MFEs of rG4 secondary structures (ΔG^0^_rG4_) were computed by subtracting MFEs obtained when considering rG4 formation into the structure prediction algorithm to the previous values (ΔG^0^_rG4_ = ΔG^0^_dsRNA_ − ΔG^0^_dsRNA + rG4_).”

should be corrected to:

“MFEs of rG4 secondary structures (ΔG^0^_rG4_) were computed by subtracting the previous values to MFEs obtained when considering rG4 formation into the structure prediction algorithm (ΔG^0^_rG4_ = ΔG^0^_dsRNA + rG4_ − ΔG^0^_dsRNA_).”
