# Supplementary material for: RNA G-quadruplexes at upstream open reading frames cause DHX36- and DHX9-dependent translation of human mRNAs
Source: Genome Biol. 2018 Dec 27;19:229. doi: 10.1186/s13059-018-1602-2 (PMC6307142; doi:10.1186/s13059-018-1602-2)
Supplement: Supplementary file 1 — Figure S1. Ribosome profiling of HeLa cells. Figure S2. Biophysical characterization of the rG4 motif found in the 5′-UTR of EED. Figure S3. Characterization of 5′-UTR translation in HeLa cells. Figure S4. Contribution of known cis-regulatory elements to translation efficiency. Figure S5. Principal component analysis (PCA) and statistical modelling of RPFdist variation. Figure S6. Polysome profiling allows assessing helicase enrichment in polysomes. Figure S7. Ribosome profiling defines the role of DHX36 and DHX9 in translation. Figure S8. Motifs discovery and analysis within the 5′-UTR of DHX9- and DHX36-dependent mRNAs. Figure S9. Characterization of DHX36- and DHX9-dependent uORFs. Figure S10. Reproducibility of the DHX9 iCLIP experiment. Figure S11. Characterization of DHX9 iCLIP peaks. Figure S12. Biophysical characterization of the rG4 motif found in the 5′-UTR of DDX23. Figure S13. DHX36- and DHX9-dependent transcripts. Figure S14. Mutation and expression profiles of DHX36 and DHX9 in cancer. Figure S15. rG4s stimulate the repressive effect of uORFs in a DHX36- and DHX9- dependent manner. (PDF 5852 kb) [file 13059_2018_1602_MOESM1_ESM.pdf]

Additional File 1

Supplementary Figures

RNA G-quadruplexes at upstream open reading frames cause DHX36- and DHX9-dependent translation of human mRNAs

Pierre Murat[1,2], Giovanni Marsico[2], Barbara Herdy[2], Avazeh Ghanbarian[2], Guillem Portella[1] and Shankar Balasubramanian[1,2,3,*]

[1] Department of Chemistry, University of Cambridge, Lensfield Road, Cambridge CB2 1EW, UK.

[2] Cancer Research UK Cambridge Institute, University of Cambridge, Li Ka Shing Centre, Robinson Way, Cambridge CB2 0RE, UK.

[3] School of Clinical Medicine, University of Cambridge, Cambridge CB2 0SP, UK.

* Correspondence to: Shankar Balasubramanian (sb10031@cam.ac.uk)

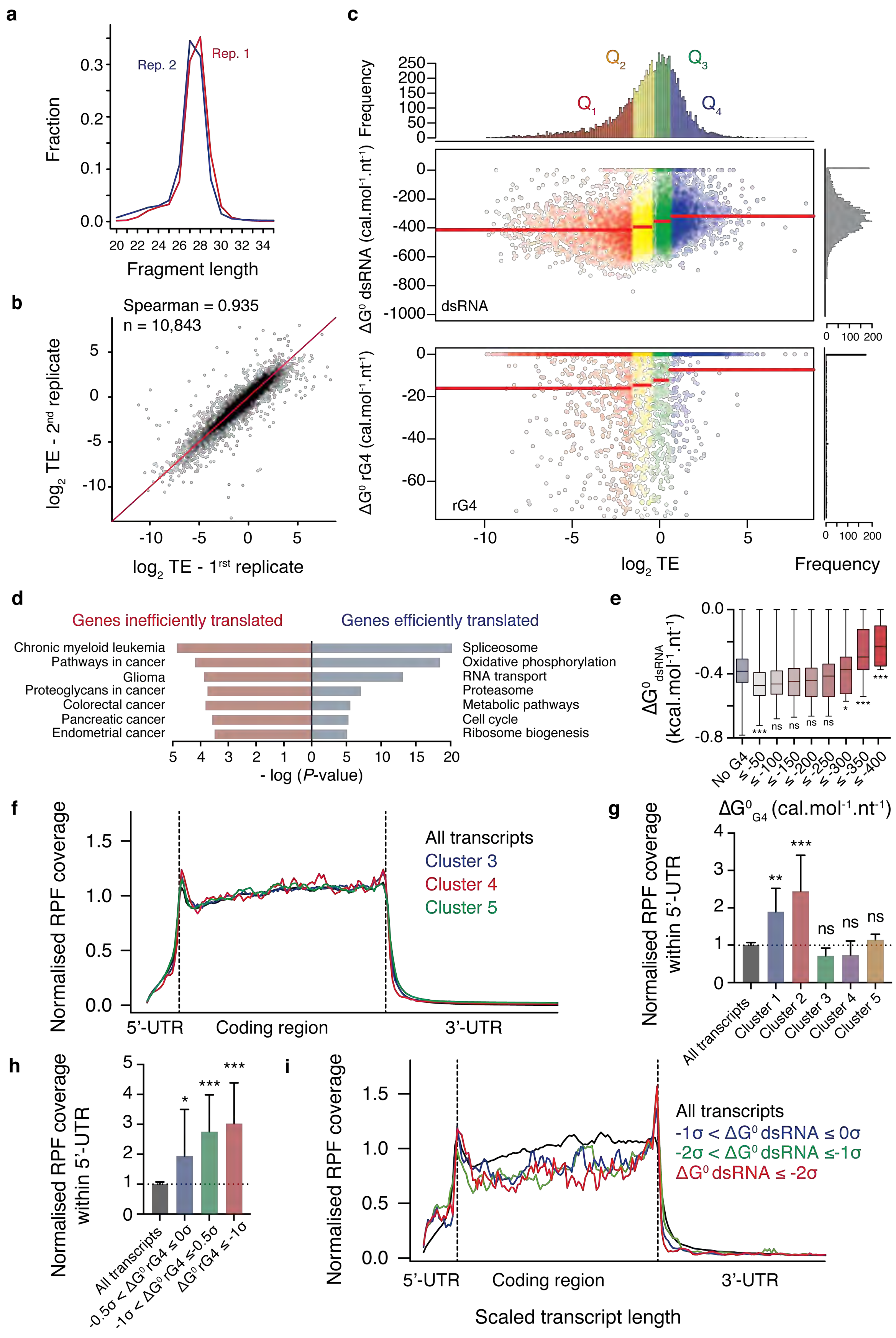
a
Fraction
Rep. 2
Rep. 1
Fragment length
b
Spearman = 0.935
n = 10,843
log2 TE - 2nd replicate
log2 TE - 1rst replicate
c
Frequency
Q1
Q2
Q3
Q4
ΔG0 dsRNA (cal.mol-1.nt-1)
dsRNA
ΔG0 rG4 (cal.mol-1.nt-1)
rG4
log2 TE
Frequency
d
Genes inefficiently translated
Genes efficiently translated
Chronic myeloid leukemia
Pathways in cancer
Glioma
Proteoglycans in cancer
Colorectal cancer
Pancreatic cancer
Endometrial cancer
Spliceosome
Oxidative phosphorylation
RNA transport
Proteasome
Metabolic pathways
Cell cycle
Ribosome biogenesis
- log (P-value)
e
ΔG0dsRNA (kcal.mol-1.nt-1)
No G4
≤ -50
≤ -100
≤ -150
≤ -200
≤ -250
≤ -300
≤ -350
≤ -400
ΔG0G4 (cal.mol-1.nt-1)
f
Normalised RPF coverage
All transcripts
Cluster 3
Cluster 4
Cluster 5
5'-UTR
Coding region
3'-UTR
g
Normalised RPF coverage within 5'-UTR
All transcripts
Cluster 1
Cluster 2
Cluster 3
Cluster 4
Cluster 5
h
Normalised RPF coverage within 5'-UTR
All transcripts
-0.5σ < ΔG0 rG4 ≤ 0σ
-1σ < ΔG0 rG4 ≤ -0.5σ
ΔG0 rG4 ≤ -1σ
i
Normalised RPF coverage
All transcripts
-1σ < ΔG0 dsRNA ≤ 0σ
-2σ < ΔG0 dsRNA ≤ -1σ
ΔG0 dsRNA ≤ -2σ
5'-UTR
Coding region
3'-UTR
Scaled transcript length

Fig. S1 Ribosome profiling of HeLa cells. a) Ribosome-protected fragment length (in nucleotides) in cycloheximide-treated HeLa samples for two replicates. **b)** The plot shows TE correlation between the two independent replicates for the 10,843 transcripts detected (> 1TPM). **b)** Plots showing the distribution of TE, 5'-UTR length-corrected minimum free energies of folded RNA secondary structures, ΔG^0_{dsRNA} and ΔG^0_{rG4}, together with the correlations between minimum free energies and TE for all HeLa mRNAs. Quartiles are defined as in **Fig. 1a**. Red lines represent the average minimum free energy value over the considered TE quartile. **d)** Pathway analysis of inefficiently (1st quartile of TE distribution, red) and efficiently (4th quartile, blue) translated genes. **e)** The presence of rG4 and dsRNA structures within 5'-UTRs is mutually exclusive as seen in reporting the 5'-UTR length normalised dsRNA folding energies of 5'-UTRs binned according to their predicted 5'-UTR length normalised rG4 folding energies. Central black lines represent the medians and the other black lines represent quartile boundaries. *P*-values were assessed using one-tailed Mann-Whitney nonparametric tests. ns: non significant, $*P < 0.05$, $***P < 0.001$. **f)** Ribosome distribution for transcripts of cluster 3 (blue), cluster 4 (red) and cluster 5 (green) showed no differences from the global transcripts population (black). **g)** Normalised RPF coverage within the 5'-UTR of transcripts from clusters 1, 2, 3, 4 and 5 showing an enrichment of RPFs in cluster 1 and 2. **h)** Normalised RPF coverage within the 5'-UTR of transcripts of cluster 2 when binned for increasing predicted rG4 stability (related to **Fig. 1g**). **i)** Ribosome distribution for transcripts of cluster 2 when binned for increasing predicted dsRNA stability, $-1\sigma < \Delta G^0_{dsRNA} \leq 0\sigma$ (blue line), $-2\sigma < \Delta G^0_{dsRNA} \leq -1\sigma$ (green line) and $\Delta G^0_{dsRNA} \leq -2\sigma$ (red line), where ΔG^0_{dsRNA} is the z-score of 5'-UTR length-normalised dsRNA predicted minimum free energy. Ribosome footprint coverage and transcript length are normalized; dotted lines indicates annotated translation start and stop sites. Clusters are defined as reported in **Fig. 1d**. Data in panels **g** and **h** are means ± s.e.m, P-values were assessed using one-tailed Mann-Whitney nonparametric tests and represent statistical difference between the binned population and the rest of the population. $*P < 0.05$, $**P < 0.01$, $***P < 0.001$, ns : non significant.

```
g4-EED:     GGGAGGGCGGCGGGAAAAGGGCAAGACGGGAGUUGGGGAAGGG
g4-EED-mut: GAGAGAGCGGCGAGAAAAGAGCAAGACGAGAGUUGAAGAAGAG
```

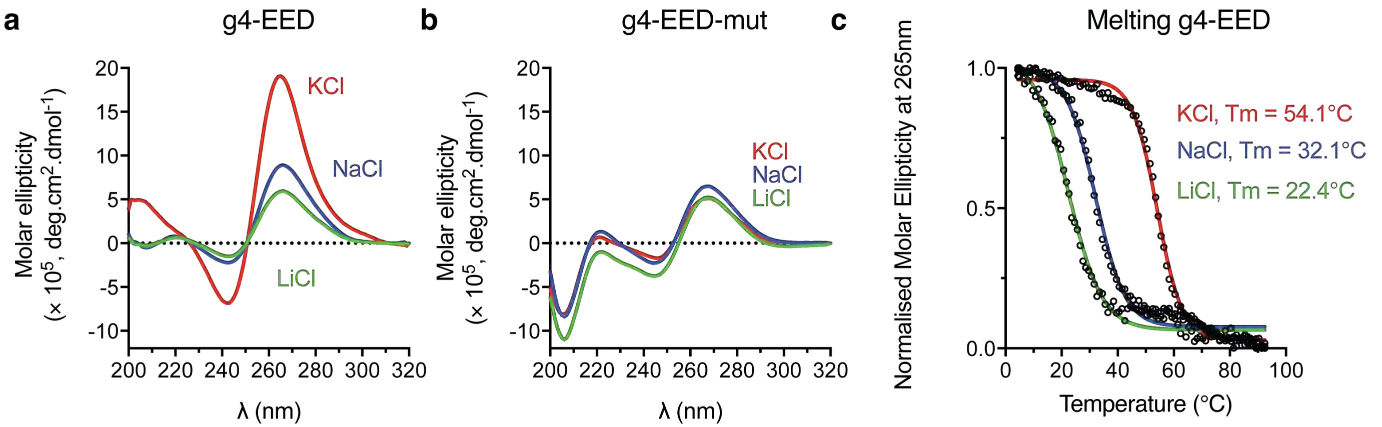

Fig. S2 Biophysical characterisation of the rG4 motif found in the 5'-UTR of *EED*. Circular dichroism (CD) characterisation of the *EED* (ENST00000263360) 5'-UTR quadruplex forming sequence (g4-EED) and a G-to-A mutant (g4-EED-mut). CD spectra of (**a**) g4-EED and (**b**) g4-EED-mut in the presence of 1 mM LiCl (green line), NaCl (blue line) or KCl (red line). The folding of g4-EED, but not g4-EED-mut, is cation dependent. The CD spectra of g4-EED display a maximum and a minimum at 265 and 240 nm, which is characteristic of rG4 structures. **c**) Denaturation of g4-EED in the presence of 1 mM LiCl (green line), NaCl (blue line) or KCl (red line) and corresponding melting temperatures. The folding and stability of g4-EED is cation dependent, which is characteristic of rG4 structures. G-to-A mutation prevents formation of the rG4 motif.

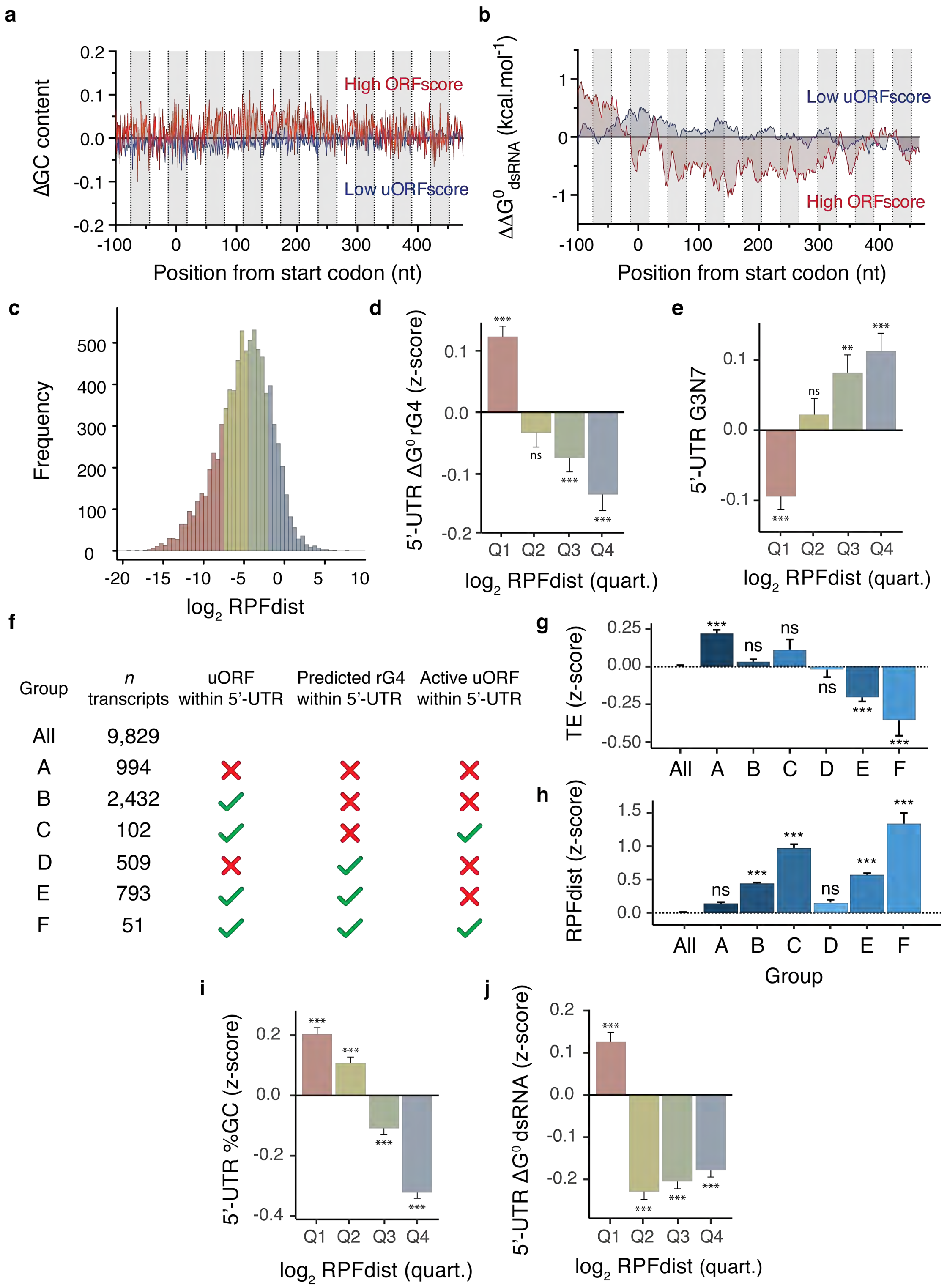

Fig. S3 Characterisation of 5'-UTR translation in HeLa cells. Base composition analysis (**a**) and dsRNA structure prediction (**b**) of sequences around the position of upstream start codons. Δ%GC and ΔMFE were calculated per

position using a sliding window of 35 nt and then normalised by subtracting the values of the background set. The grey and white areas represent the size of 80S ribosomes phased on the upstream start codon. Background are uORFs with negative ORFscore, Low (ORFscore < 6) and High (ORFscore ≥ 6) ORFscore uORFs are uORFs that are unlikely to be translated and uORFs presenting RPF signature consistent with active translation respectively. Refer to the **Method** section for ORFscore calculation. **c)** Transcriptome-wide distribution of RPFdist values of human mRNAs coloured by quartile. Correlations between (**d**) 5'-UTR length-normalised rG4 predicted 5'-UTR folding energies or (**e**) the presence of G3N7 motifs and RPFdist. **f**) mRNAs were grouped according to the presence of uORFs, rG4 (predicted using RNAfold) and/or translated uORFs within 5'-UTRs and the average (**g**) TE and (**h**) RPFdist values of each group were calculated. It is noteworthy that the presence of uORFs within 5'-UTRs (group B) does not affect the TE of mRNAs. The presence of active uORFs, *i.e.* high ORFscore uORFs (group C), affects ribosome distribution but not TE. The presence of rG4s, but not of uORFs, within 5'-UTRs (group D), does not affect TE nor RPFdist. Inefficient translation and altered ribosome distribution are observed only when both uORFs and rG4s are present within the 5'-UTR of a mRNA (group E). This effect is stressed when the uORFs display sign of active translation (ORFscore ≥ 6, group F). Correlations between (**i**) 5'-UTR GC content, (**j**) 5'-UTR length-normalised dsRNA predicted 5'-UTR folding energies and RPFdist. Each feature is expressed as z-score and RPFdist is binned in quartiles (for panels **d**, **e**, **i** and **j**). Data are means ± s.e.m, *P*-values were assessed using one-tailed Mann-Whitney nonparametric tests and compare the reported condition to the rest of the population. ns non-significant, ***P* < 0.01, ****P* < 0.001.

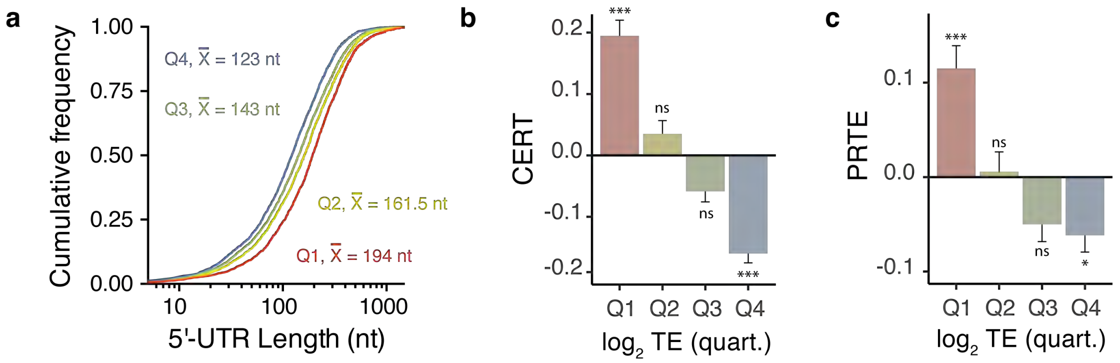

Fig. S4 Contribution of known *cis*-regulatory elements to translation efficiency. a) Comparison of 5'-UTR length (together with the median of the lengths) for transcripts showing different TE. Correlations between the number of known *cis*-regulatory elements such as Cytosine Enriched Regulator of Translation (CERT, **b**) or Pyrimidine-rich translation element (PRTE, **c**) and TE. Each feature is expressed as z-score and TE is binned in quartiles (definition of the different *cis*-regulatory elements is reported in the **Supplementary Information**). Data are means ± s.e.m, *P*-values were assessed using one-tailed Mann-Whitney nonparametric tests and compare the reported condition to the rest of the population. ns non-significant, $^{**}P < 0.01$, $^{***}P < 0.001$.

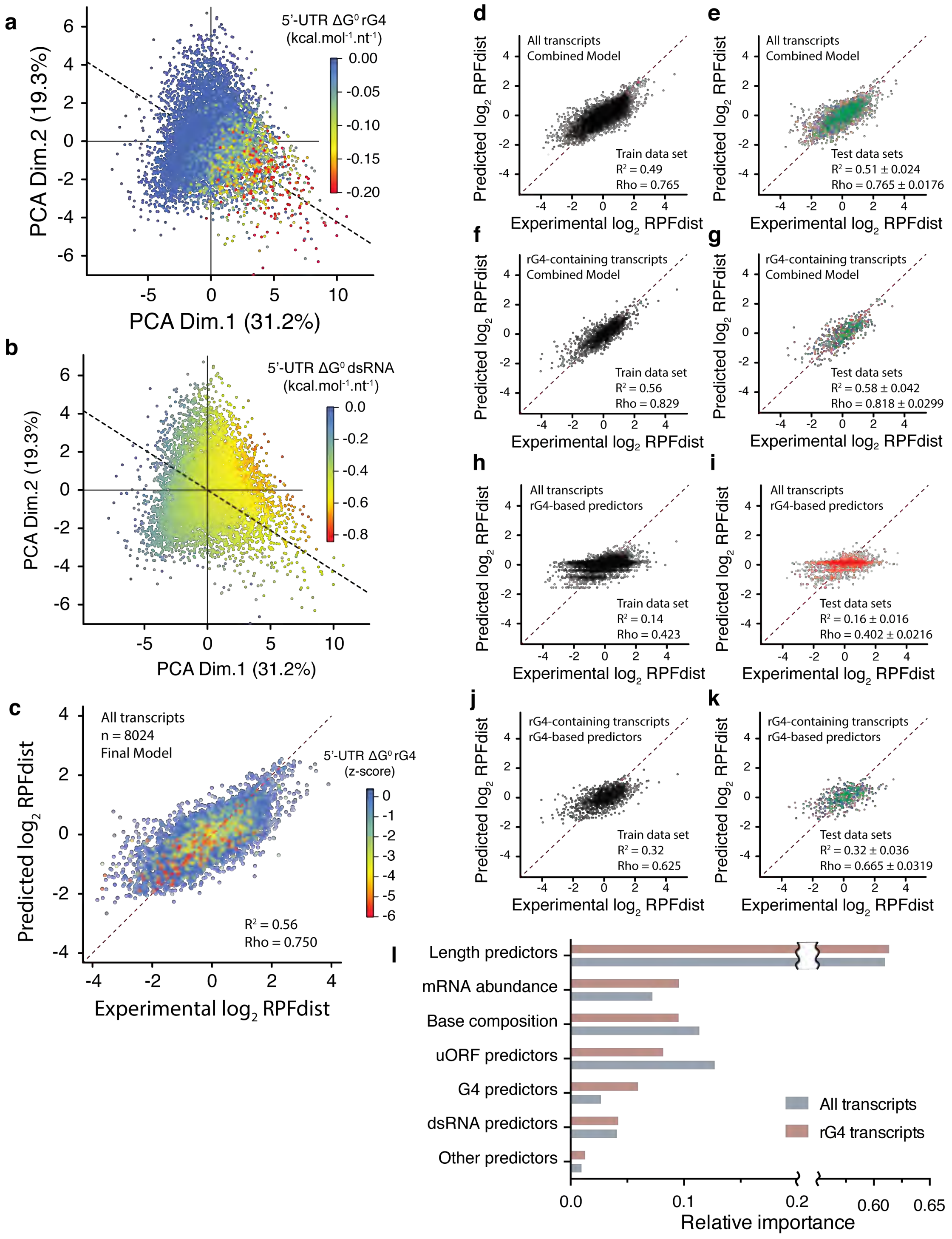

Fig. S5 Principal component analysis (PCA) and statistical modelling of RPFdist variation. Principal component analysis of human transcripts performed similarly as in **Fig. 2d** but reporting individual mRNAs coloured according to 5'-UTR length-normalised 5'-UTR (**a**) rG4 or (**b**) dsRNA predicted folding energies. It is noteworthy, that dsRNA and rG4 features are separated by the second component. The dotted black line represents the projection of TE unto the plan of the PCA. **c)** A statistical model with as few as 32 predictors explains 56% of the RPFdist variation

observed in our dataset (all detected transcripts with fully annotated 5'-UTRs (with a length ≥ 10 nt) and 3'-UTRs, $n = 8{,}024$). The performance of our models, selected on training sets (**d** and **f**), was assessed by challenging them on 3 independent test sets (**e** and **g**). Similar R^2 and Pearson correlation (*Rho*) were found when comparing predicting and experimental values supporting the robustness of our model selection pipeline (detailed in **Supporting Information**). Panel **h** to **k** reports the performance of our models selected to predict RPF variation of the global population of transcripts and the rG4-containing 5'-UTR subset when considering only rG4-based predictors. **l**) Relative importance of each category of predictors in the final models selected to predict RPF variation in global population of transcripts and the rG4-containing 5'-UTR subset. Taken together, these results demonstrate that rG4s are determinants of ribosome distribution within transcripts displaying clear signature of rG4 structures within their 5'-UTRs (defined by the PCA with Dim.1 ≥ 0 and Dim.2 ≤ 0).

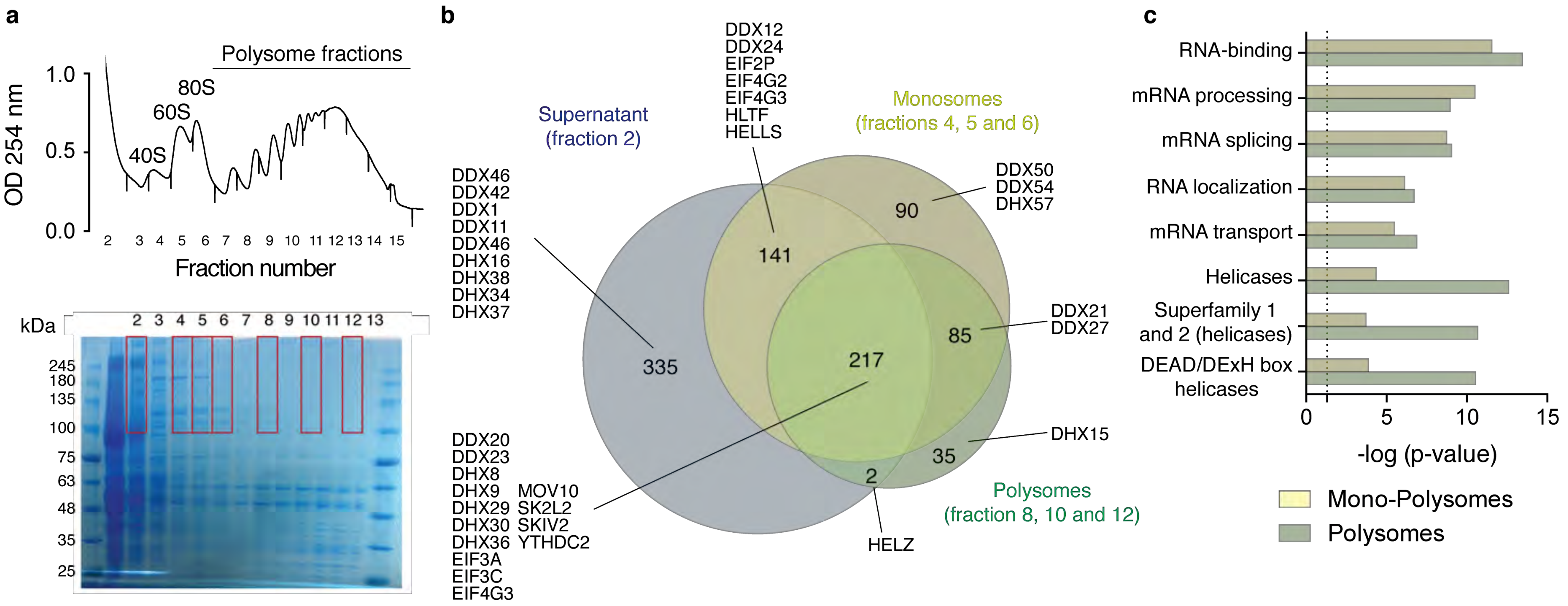

Fig. S6 Polysome profiling allows assessing helicase enrichment in polysomes. a) Cycloheximide-treated HeLa cytoplasmic extracts were loaded onto 10% - 50% sucrose gradients and centrifuge at high-speed. Supernatant (fraction 2), monosomes (fraction 3 to 6) and polysomes (fraction 7 to 15) were fractioned and loaded onto acrylamide gels. High molecular protein complexes (Mw ≥ 100 kDa, cut bands are highlighted in red) were analysed by mass spectrometry. **b)** Venn diagram reporting the number of proteins identified in each fraction of the polysome profiling experiment performed in duplicates. **c)** Functional analysis of identified proteins associated with mono/polysomes (fractions 4, 5, 6, 8, 10 and 12) or with polysomes (fractions 8, 10 and 12) revealed an enrichment of DEAD and DExH-box in the polysomes fractions.

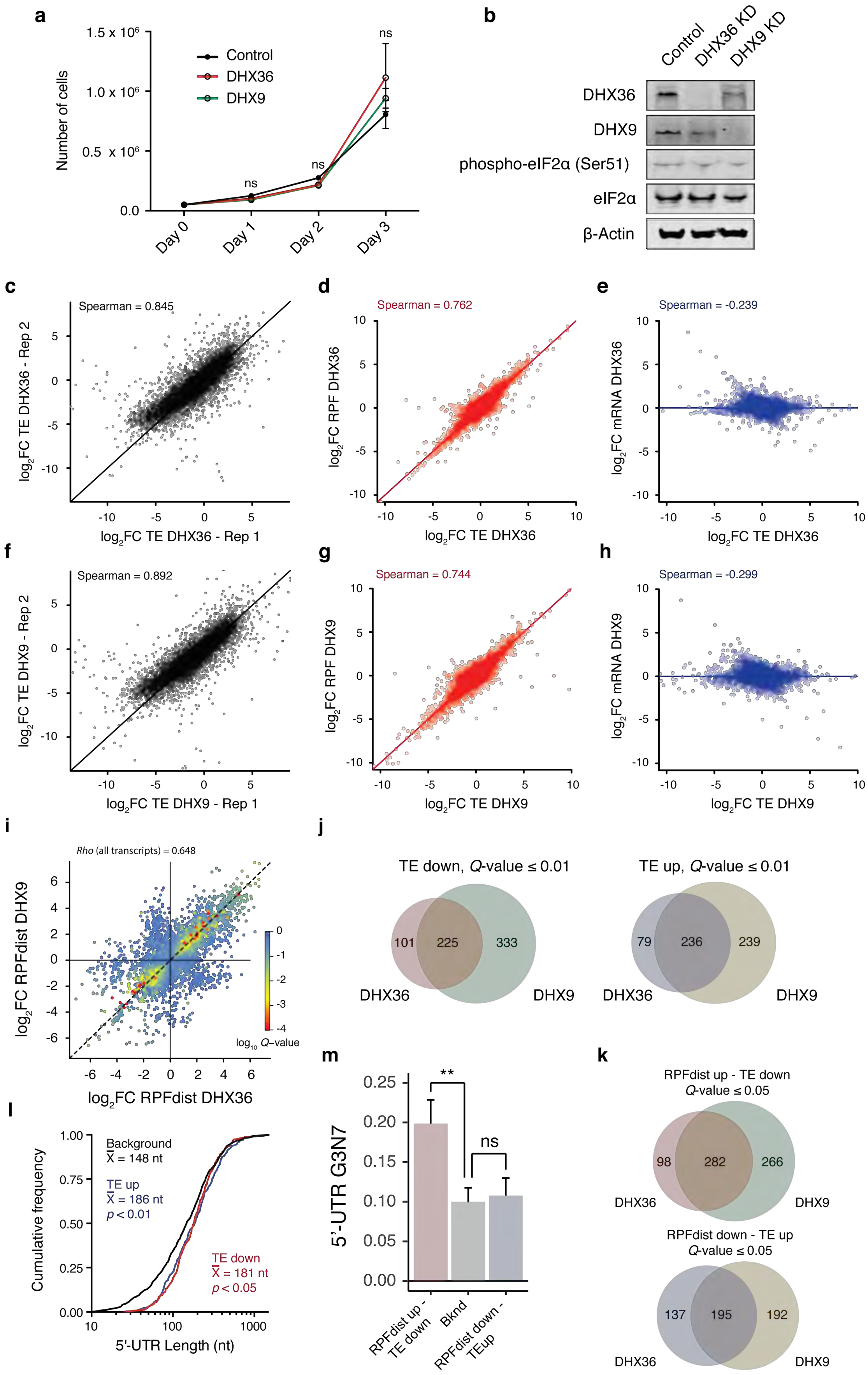
a
1.5 x 10^6
1.0 x 10^6
0.5 x 10^6
0.0
Number of cells
Control
DHX36
DHX9
ns
ns
ns
Day 0
Day 1
Day 2
Day 3
b
Control
DHX36 KD
DHX9 KD
DHX36
DHX9
phospho-eIF2α (Ser51)
eIF2α
β-Actin
c
Spearman = 0.845
log2FC TE DHX36 - Rep 2
log2FC TE DHX36 - Rep 1
d
Spearman = 0.762
log2FC RPF DHX36
log2FC TE DHX36
e
Spearman = -0.239
log2FC mRNA DHX36
log2FC TE DHX36
f
Spearman = 0.892
log2FC TE DHX9 - Rep 2
log2FC TE DHX9 - Rep 1
g
Spearman = 0.744
log2FC RPF DHX9
log2FC TE DHX9
h
Spearman = -0.299
log2FC mRNA DHX9
log2FC TE DHX9
i
Rho (all transcripts) = 0.648
log2FC RPFdist DHX9
log2FC RPFdist DHX36
log10 Q–value
j
TE down, Q-value ≤ 0.01
101
225
333
DHX36
DHX9
TE up, Q-value ≤ 0.01
79
236
239
DHX36
DHX9
l
Cumulative frequency
Background
X = 148 nt
TE up
X = 186 nt
p < 0.01
TE down
X = 181 nt
p < 0.05
5'-UTR Length (nt)
m
5'-UTR G3N7
**
ns
RPFdist up - TE down
Bknd
RPFdist down - TEup
k
RPFdist up - TE down
Q-value ≤ 0.05
98
282
266
DHX36
DHX9
RPFdist down - TE up
Q-value ≤ 0.05
137
195
192
DHX36
DHX9

Fig. S7 Ribosome profiling defines the role of DHX36 and DHX9 in translation. a) Cell proliferation assay of cells depleted in DHX36 and DHX9 or treated with a pool of non-targeting siRNAs (control). Cells were treated with siRNAs for 24h and the number of cells were then monitored for 3 days. No significant difference in number of cells was observed when comparing the different conditions. **b)** Immunoblots of siRNA-treated cells probing eIF2α (Cell signalling antibody #9722) and phospho-eIF2α (Ser51) (Cell signalling antibody #3398) show that siRNA depletion of both helicases does not trigger eIF2α phosphorylation. **c)** and **f)** Plots showing the correlation between fold changes in TE for two independent replicates upon depletion of DHX36 and DHX9. Correlation between changes in RPF (**d** and **g**) or mRNA (**e** and **h**) and changes in TE indicate that TE variations reflect translational rather than transcriptional variations. (**i**) Correlation between changes in RPFdist in DHX36 and DHX9 samples. *Q*-values were calculated by combining *P*-values associated to RPFdist variation using Fisher's method. **j-k)** Venn diagrams reporting the overlap between the transcripts whose TE or RPFdist values changed upon DHX36 and DHX9 depletion. Transcripts were binned according to the polarity of their TE or RPFdist values variation. All reported groups overlap significantly ($P <$ 0.01, Fisher exact test), which supports that DHX36 and DHX9 shares the same mRNA targets. **l)** 5'-UTR length of the TE down and TE up group of transcripts compared to background (unchanged TE). Both TE down and TE up mRNAs have longer 5'-UTRs. *P*-values were calculated using an unpaired student's t-test. **m)** Enrichment of the G3N7 quadruplex-forming motif in the RPFdist up – TE down group of mRNAs. Data are means ± s.e.m, *P*-values were assessed using one-tailed Mann-Whitney nonparametric tests. ns non-significant, $^{**}P < 0.01$. Report to the main manuscript for the definition of each group.

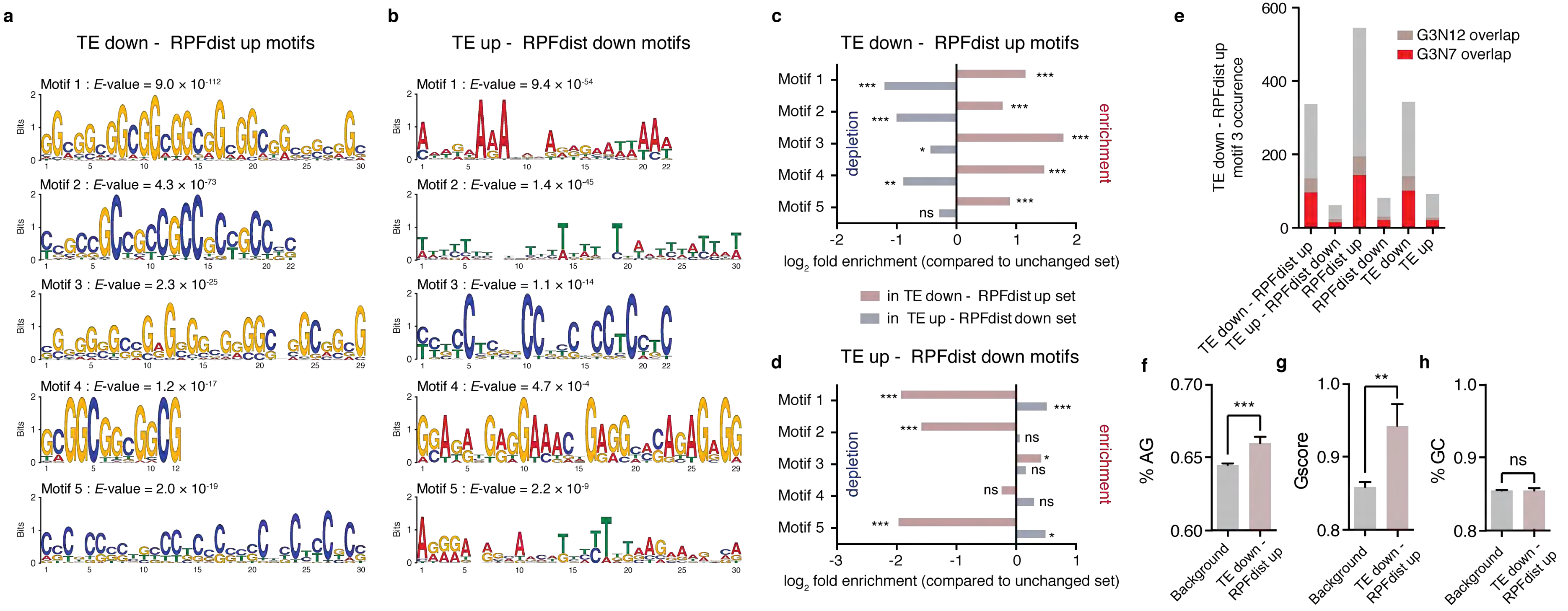

Fig. S8 Motifs discovery and analysis within the 5'-UTR of DHX9- and DHX36-dependent mRNAs. De novo motif discovery and analysis were performed using the Meme suite (see **Method** section). Here we report the five more enriched motifs in the 5'-UTR of the (**a**) TE down – RPFdist up and (**b**) TE up – RPFdist down groups. *E*-values refer to enrichment when compared to control set (5'-UTR sequences of all expressed mRNA in HeLa cells). In a second step, motifs enrichments (**c-d**) were calculated by comparing the density of a given motifs to the density of the same motif in the unchanged set, defined as transcripts with FC TE between -0.1 and 0.1. *P*-values were assessed using two-sided Fisher exact tests. ns non-significant, $^{*}P < 0.05$, $^{**}P < 0.01$, $^{***}P < 0.001$. **e)** Occurrence and rG4-forming motifs overlap of the TE down – RPFdist up most enriched motif (motif 3 in panel **a**) in the different group of mRNAs. **(f-g)** Base composition analysis

of the TE down – RPFdist up most enriched motif in the background or TE down – RPFdist up transcripts. Sequences corresponding to the identified motif ± 10 nt were considered to reflect the sequences context. The motifs were found enriched in purine and more particularly in guanine when in the 5'-UTR of TE down – RPFdist up transcripts. No differences in GC content were observed. Data are means ± s.e.m, *P*-values were assessed using one-tailed Mann-Whitney nonparametric tests. ns non-significant, $^{**}P < 0.01$, $^{***}P < 0.001$.

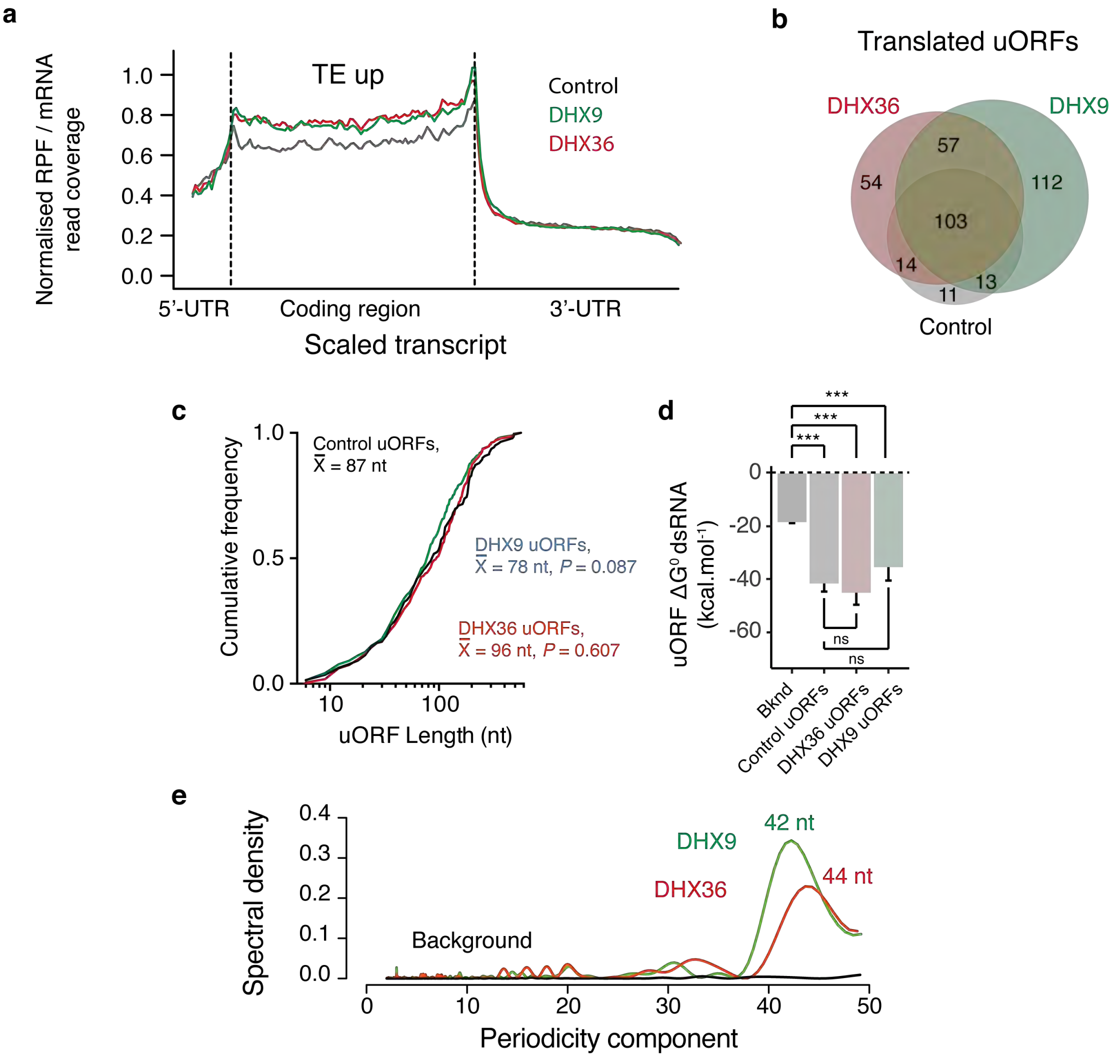

Fig. S9 Characterisation of DHX36- and DHX9-dependent uORFs. a) Ribosome distribution normalised by mRNA signal, describing local translation efficiency, of the TE up group (918 transcripts with combined Q-value ≤ 0.05) in control (black), DHX36 (red) and DHX9 (green) depleted cells. Ribosome footprint, mRNA signal coverages and transcript length are normalized; dotted lines indicates annotated translation start and stop sites. **b)** Venn diagram depicting total number of detected high ORFscore uORFs in control (black), DHX36 (red) and DHX9 (green) depleted cells. Depletion of the helicases induces the translation of new uORFs. **c)** Length of DHX36- and DHX9-dependent uORFS compared to control uORFs (detected translated uORFs in control cells). *P*-values were calculated using an unpaired student's t-test. **d)** Predicted dsRNA structure folding energies of detected high ORFscore uORFs in control (grey) or DHX36 (red) and DHX9 (green) depleted cells. The background set (black) represents uORFs with negative ORFscore in

control cells. Data are means ± s.e.m, *P*-values were assessed using one-tailed Mann-Whitney nonparametric tests. ns non-significant, ***$P < 0.001$. **e**) Periodograms obtained from the position of rG4s within the DHX36- and DHX9-dependent uORFs (**Fig. 4h**) highlighting periodicities of 42-44 nt.

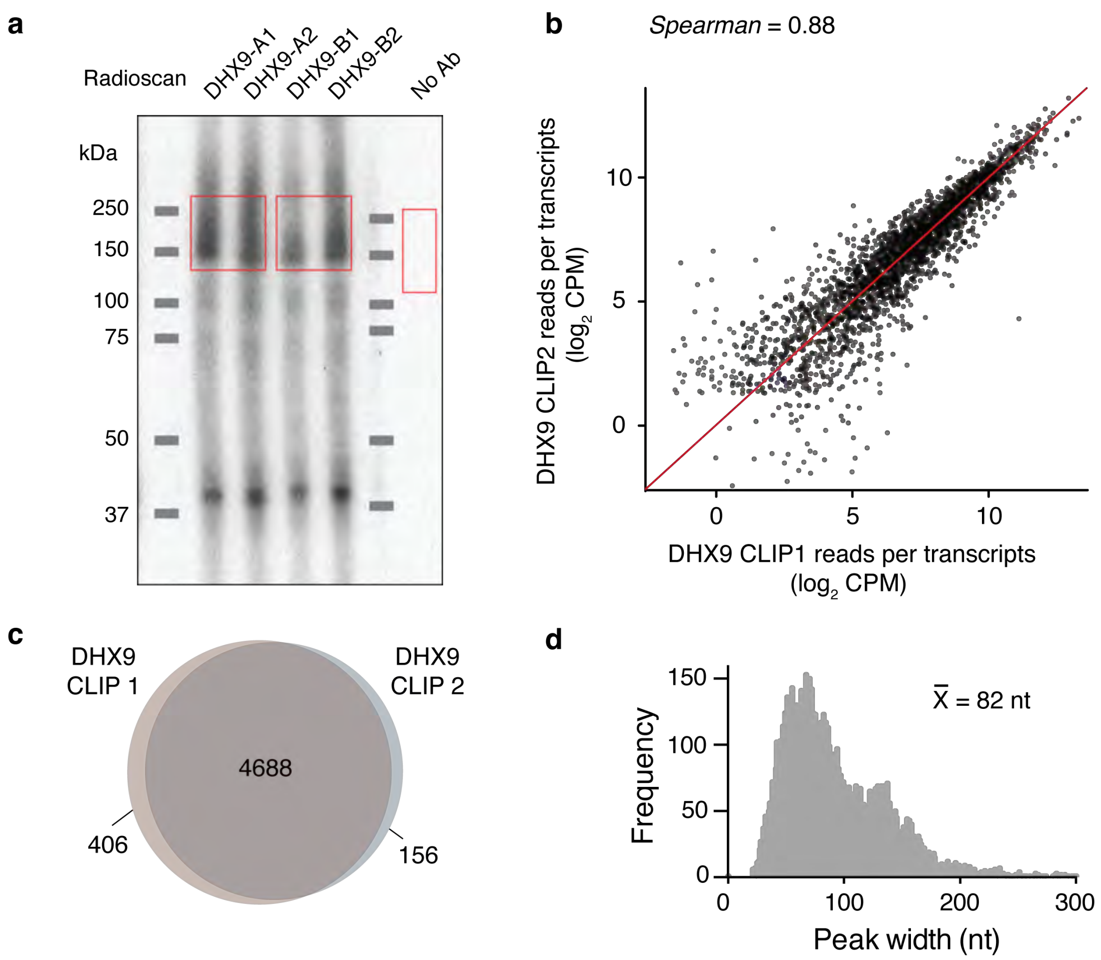

Fig. S10 Reproducibility of the DHX9 iCLIP experiment. a) Two biological replicates of DHX9 iCLIP were performed. This panel reports a phosphorimage of a SDS gel resolving ^{32}P-labelled RNAs crosslinked to DHX9. Each lane represents immunoprecipitated RNA from 4 150 mm plates of HeLa cells. The materials from two lanes were combined to generate the two biological replicates. The last lane of the gel shows a negative control obtained when omitting the DHX9 antibody during the immunoprecipitation step. **b)** The plot shows the correlation of DHX9 iCLIP reads (expressed in CPM per transcripts) between the two replicates. **c)** Venn diagram reporting the overlap between the peaks called for the second DHX9 iCLIP experiments and the peaks called for the first replicate. **d)** Peak width distribution (in nucleotides) after multimodal peak splitting (refer to the **Method** section for more details).

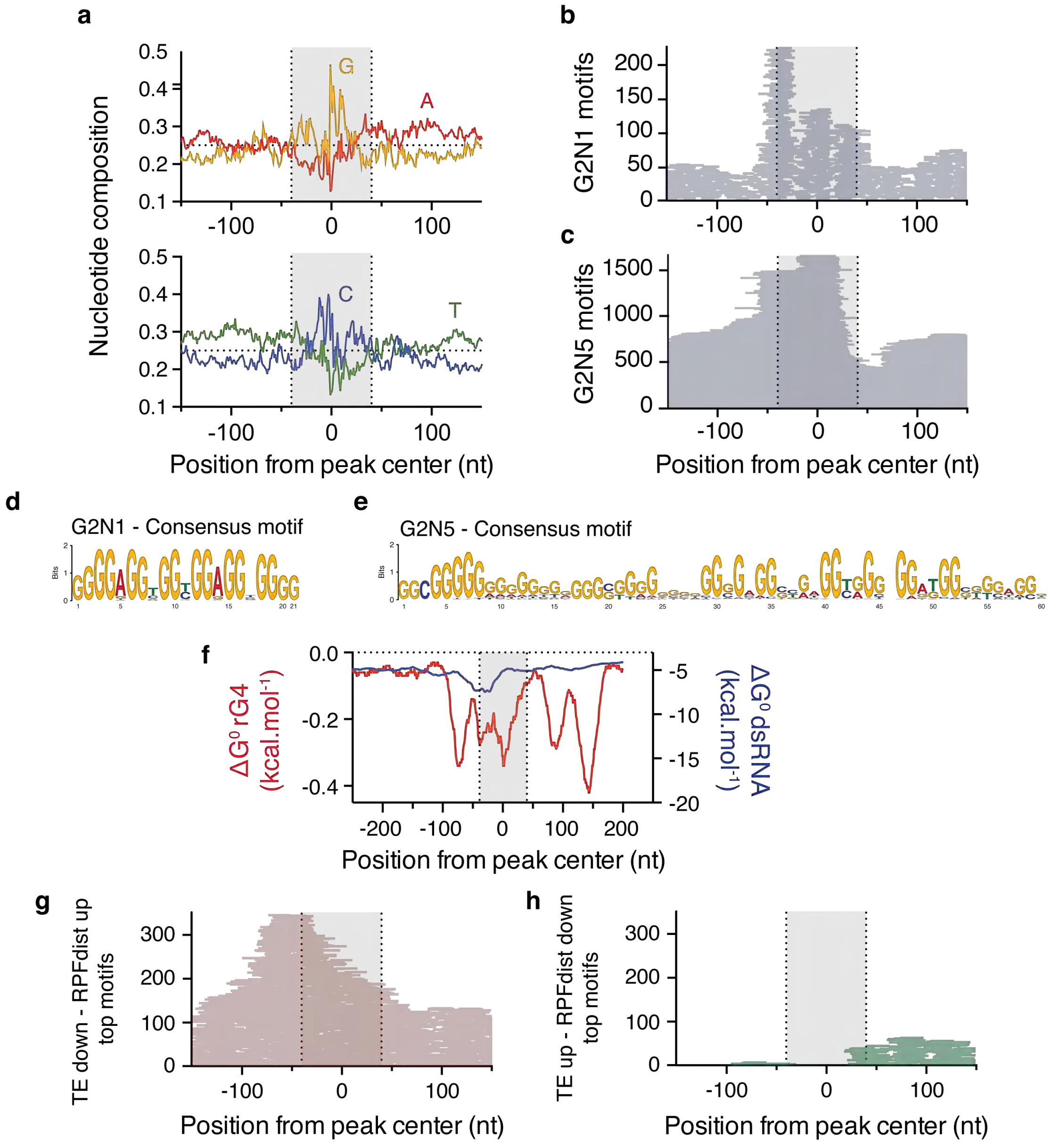

Fig. S11 Characterisation of DHX9 iCLIP peaks. a) Base composition analysis of DHX9 iCLIP peaks. **b-c)** Distribution of rG4 motifs, of the form G2N1 and G2N5, around the centre of iCLIP peaks. **d-e)** Consensus motif of G2N1 and G2N5 rG4-forming sequences found upstream DHX9 iCLIP peaks. **f)** rG4 and dsRNA predicted folding energies within 200 bases from the centre of DHX9 iCLIP peaks. Enrichment of rG4s downstream the DHX9 peaks centre is likely reflecting the multimodal nature of DHX9 iCLIP peaks. Minimum folding energies were calculated using a sliding window of 35 nt. **g-h)** Distribution the TE down –RPFdist up and TE up – RPFdist down top motifs around the centre of iCLIP peaks. The grey area represents the median width of iCLIP peaks.

g4-DDX23: GGGCGGUUCAGACUCAGGGUGUAGAGAUGGGG

g4-DDX23-mut: GAGCGAUUCAGACUCAGAGUGUAGAGAUGAAG

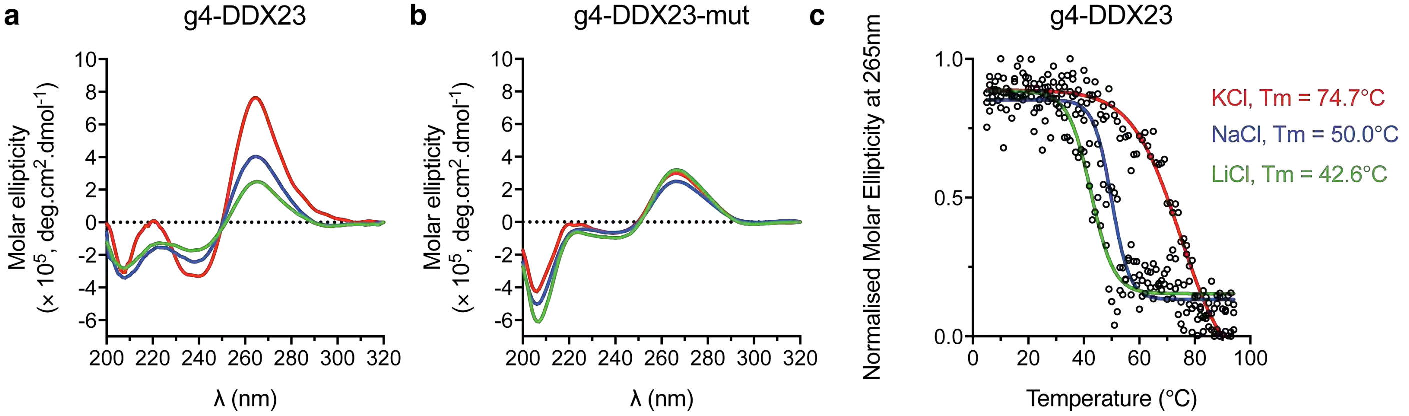

Fig. S12 Biophysical characterisation of the rG4 motif found in the 5'-UTR of *DDX23*. Circular dichroism (CD) characterisation of the *DDX23* (ENST00000551468) 5'-UTR quadruplex forming sequence (g4-DDX23) and a G-to-A mutant (g4-DDX23-mut). CD spectra of (**a**) g4-DDX23 and (**b**) g4-DDX23-mut in the presence of 100 mM LiCl (green line), NaCl (blue line) or KCl (red line). The folding of g4-DDX23, but not g4-DDX23-mut, is cation dependent. The CD spectra of g4-DDX23 display a maximum and a minimum at 265 and 240 nm, which is characteristic of rG4 structures. **c**) Denaturation of g4-DDX23 in the presence of 100 mM LiCl (green line), NaCl (blue line) or KCl (red line) and corresponding melting temperatures. The folding and stability of g4-DDX23 is cation dependent, which is characteristic of rG4 structures. G-to-A mutation prevents formation of the rG4 motif.

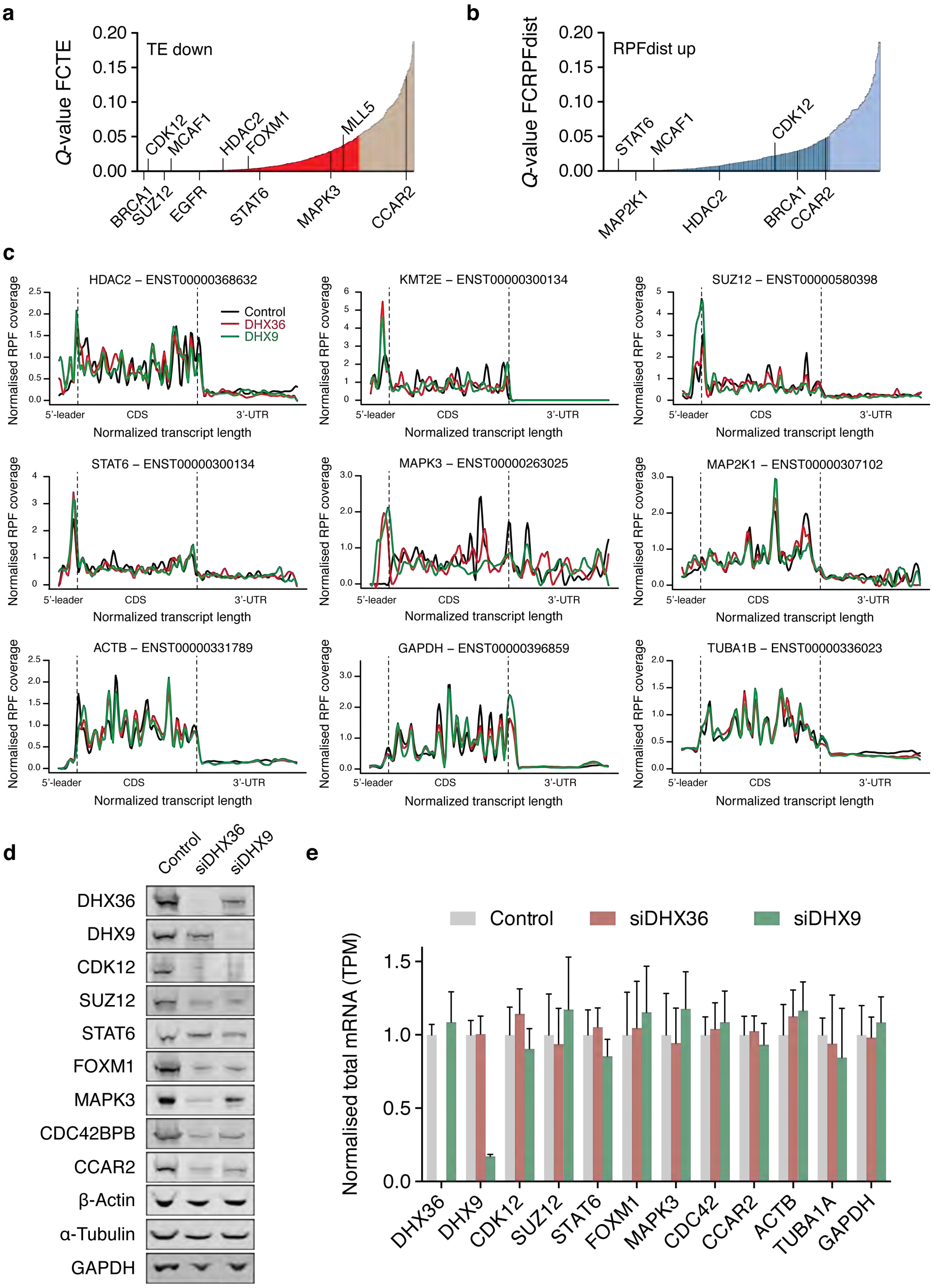

Fig. S13 DHX36- and DHX9-dependent transcripts. a) TEdown (Q < 0.05) and (**b**) RPFdistup (Q < 0.05) genes ranked by significance. TEdown and RPFdistup transcripts include many genes involved in cancer pathways. **c)** Normalised ribosome distribution for the indicated transcripts, n = 3 replicates, in control (black), DHX36 (red) and DHX9 (green) depleted cells. Transcript length are normalized; dotted lines indicates annotated translation start and stop sites. Profiles of DHX36-

and DHX9-dependent mRNAs presented altered distribution in 5'-leader sequences, while house keeping genes (ACTB, GAPDH and TUBA1B) did not show any enrichment of RPF in their 5'-UTR. **d)** Immunoblots of lysates from HeLa cells depleted in DHX36 and DHX9 and probed as indicated (Biological replicate of experiment presented in **Fig. 6d**. **e)** mRNA levels (in TPM) for the indicated genes in control (black), DHX36 (red) and DHX9 (green) depleted cells. Data represent the mean and s.d., *n* = 3 biological replicates. mRNA levels are from the RNA-seq experiments performed in parallel to the Ribo-seq experiments. For genes encoded by more than one transcript, the level of the most abundant mRNA is reported. It is noteworthy that DHX36 mRNA was not detected in DHX36-depleted cells.

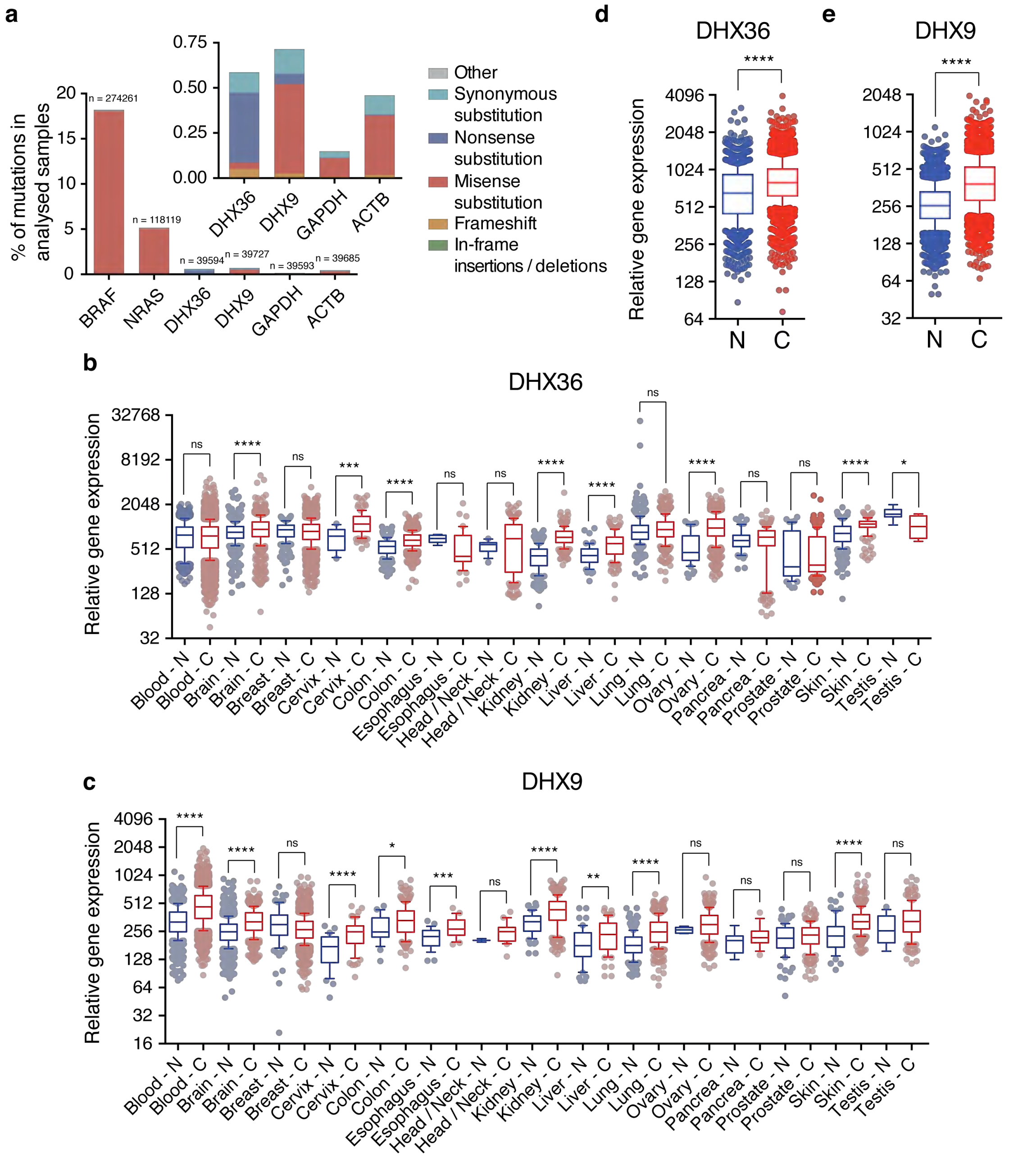

Fig. S14 Mutation and expression profiles of DHX36 and DHX9 in cancer. a) Mutation frequencies were recovered form the COSMIC database (COSMIC v82, http://cancer.sanger.ac.uk/cosmic). We searched for mutations in BRAF, NRAS (frequently mutated in cancers), DHX36, DHX9, GAPDH and ACTB (non-mutated in cancers) in all human cancers (the number of available samples are reported for each genes). Identified mutations were stratified into six classes: synonymous, nonsense and missense substitutions, frameshifts, in-frame deletions or insertions and others. The insert shows that DHX36 and DHX9 do not show any frequent mutations in human cancers. **b-c)** Expression profiles of DHX36 and DHX9

in normal and cancer tissues were recovered from the GENT database (http://medicalgenome.kribb.re.kr/GENT/) using data generated by Affymetrix U133plus2 platforms. Both DHX36 and DHX9 showed altered expression levels in brain, cervix, colon, kidney, liver and skin cancerous tissues. **d-e)** Expression profiles of DHX36 and DHX9 in normal and cancer tissues when combining data from tissues presenting altered expression profiles. These plots showed that both DHX36 and DHX9 are overexpressed in cancer tissues. In the box plots, the central lines represent the medians and the other lines represent quartile boundaries. Points represent individual values outside the 10-90 percentiles. *P*-values were assessed using two-tailed Student's t-tests. ns: non significant, *$P < 0.05$, *$P < 0.01$, ***$P < 0.001$ and ****$P < 0.0001$.

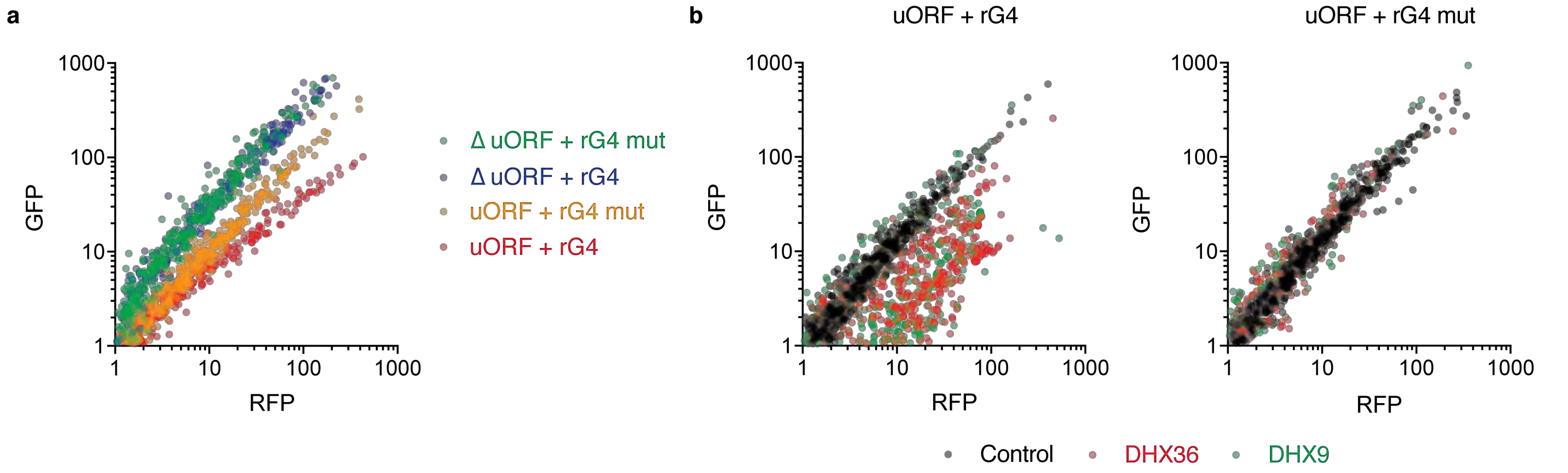

Fig. S15 rG4s stimulate the repressive effect of uORFs in a DHX36- and DHX9- dependent manner. a) Representative flow cytometry profiles of HeLa cells transfected with bicistronic reporter genes containing within their 5'-UTRs either an rG4 motif (Δ uORF + rG4), a mutated rG4 (Δ uORF + rG4 mut), an rG4-containing uORF (uORF + rG4) or an rG4-mutated uORF (uORF + rG4 mut). **b)** Representative flow cytometry profiles of HeLa cells transfected with bicistronic reporter genes containing within their 5'-UTRs either an rG4-containing uORF (uORF + rG4) or an rG4-mutated uORF (uORF + rG4 mut) and non-targeting siRNAs (control), siRNAs targeting DHX36 or DHX9. The relative translation of the reporter genes (ratio of GFP over RFP) is reported in Fig. 6e and 6f in the main manuscript.
