# Supplementary material for: RNA G-quadruplexes at upstream open reading frames cause DHX36- and DHX9-dependent translation of human mRNAs
Source: Genome Biol. 2018 Dec 27;19:229. doi: 10.1186/s13059-018-1602-2 (PMC6307142; doi:10.1186/s13059-018-1602-2)
Supplement: Supplementary file 2 — Supplementary Information. (PDF 1838 kb) [file 13059_2018_1602_MOESM2_ESM.pdf]

Additional File 2

Supplementary Information

RNA G-quadruplexes at upstream open reading frames cause DHX36- and DHX9-dependent translation of human mRNAs

Pierre Murat[1,2], Giovanni Marsico[2], Barbara Herdy[2], Avazeh Ghanbarian[2], Guillem Portella[1] and Shankar Balasubramanian[1,2,3,*]

[1] Department of Chemistry, University of Cambridge, Lensfield Road, Cambridge CB2 1EW, UK.

[2] Cancer Research UK Cambridge Institute, University of Cambridge, Li Ka Shing Centre, Robinson Way, Cambridge CB2 0RE, UK.

[3] School of Clinical Medicine, University of Cambridge, Cambridge CB2 0SP, UK.

* Correspondence to: Shankar Balasubramanian (sb10031@cam.ac.uk)

Ribosome profiling quality control...p 2

Characterisation of ribosome protected fragments.......................................p 3

Principal component analysis (PCA) and Statistical Modelling.......................p 5

Oligonucleotides used in this study..p 11

Additional references...p 12

Ribosome profiling quality control

Analysis of ribosome-protected mRNA fragments (RPFs) yields a quantitative and detailed map of ribosome occupancy that reveals translation with single-nucleotide resolution.[1] Most ribosome footprints fall within known coding sequences where they showed three-nucleotide periodicity reflecting the triplet nature of the genetic code. The periodicity plots reported in **Fig. S1** show that all the libraries prepared in this study display a robust triplet periodicity suitable for analysis of translation.

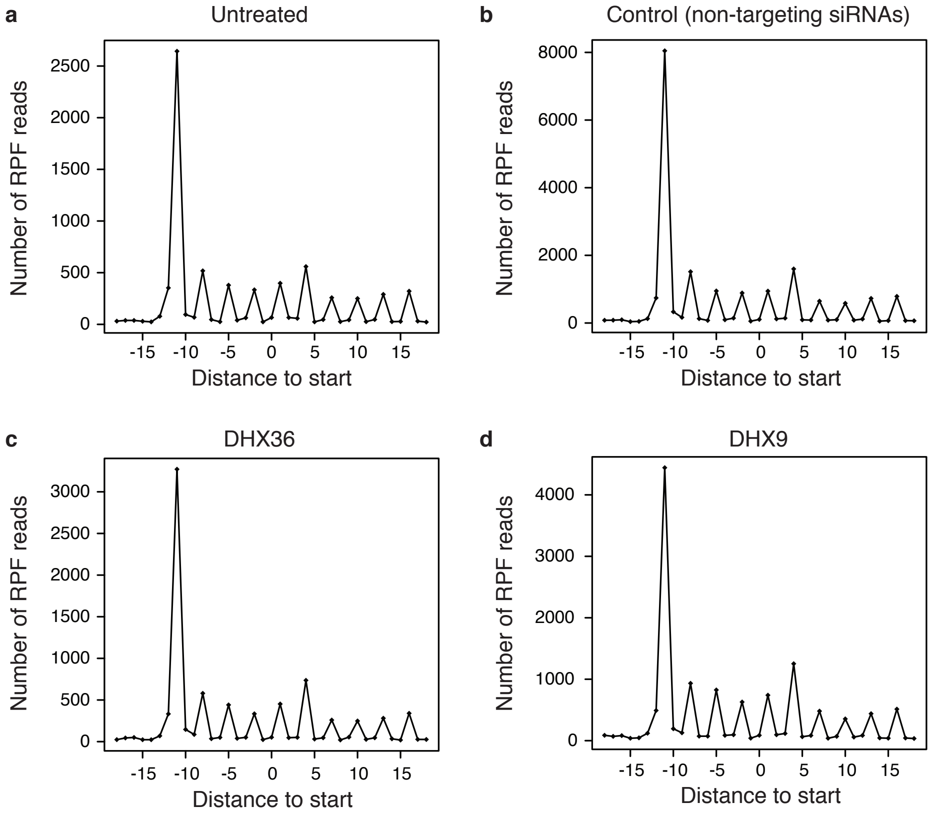

Fig. S1 Periodicity plots. Representative plots reporting the position of the 5'-ends of the 28 and 29 nt ribosome footprints falling near the beginning of annotated CDSs for ribosome profiling libraries prepared from (**a**) untreated, (**b**) non-targeting, (**c**) DHX36- or (**d**) DHX9-targeting siRNAs treated HeLa cells. 3 nts periodicity was observed across all libraries in this study.

Characterisation of ribosome protected fragments

In this work we focus on ribosome protected fragments that were mapped within the 5'-UTRs of translated mRNAs. In order to assess whether reads aligning to 5'-UTRs are true ribosome footprints, rather than nonribosomal contaminants such as RNA regions that are protected by protein complexes or stable RNA secondary structure, we applied the fragment length organization similarity score (FLOSS) pipeline developed by Ingolia et al.[2] The FLOSS measures the magnitude of disagreement between the distribution of fragments lengths mapping a region of interest to a reference distribution from all annotated nuclear protein-coding transcript. FLOSS was computed from a histogram of read lengths for footprints on an annotated CDS or within a given 5'-UTR. A reference histogram was produced using raw counts on all annotated nuclear protein-coding transcript. The FLOSS was calculated as

$$FLOSS = 0.5 \times \sum_{l=22}^{35} | f(l) - f_{ref}(l) |$$

where f(l) is the fraction of reads at length l in the transcript histogram and $f_{ref}(l)$ is the corresponding fraction in the reference histogram. Reads overlapping with annotated start codons and 5'-UTRs smaller than 10 nucleotides were excluded from the analysis. Low FLOSS characterise regions with ribosome-profiling-derived reads that are true ribosome footprints, while high FLOSS is associated with nonribosomal background. We formalized this classification by defining a threshold FLOSS value based on the read counts and FLOSS values for known protein-coding genes using Tukey's method. The FLOSS cutoff score, as a function of the total number of reads, was counted from a rolling window of individual annotated genes and the computing of the upper extreme outlier cutoff for each window using Tukey's method (Q3 + 3*IQR, where Q3 is the 3rd quartile and IQR is the interquartile range). FLOSS analysis (**Fig. S2**) revealed that 95.2 % of all analysed 5'-UTRs and 96.5 % of 5'-UTRs of mRNAs from cluster 2 (as defined in **Fig. 1d**) resemble coding sequences in our total HeLa cell ribosome profiling.

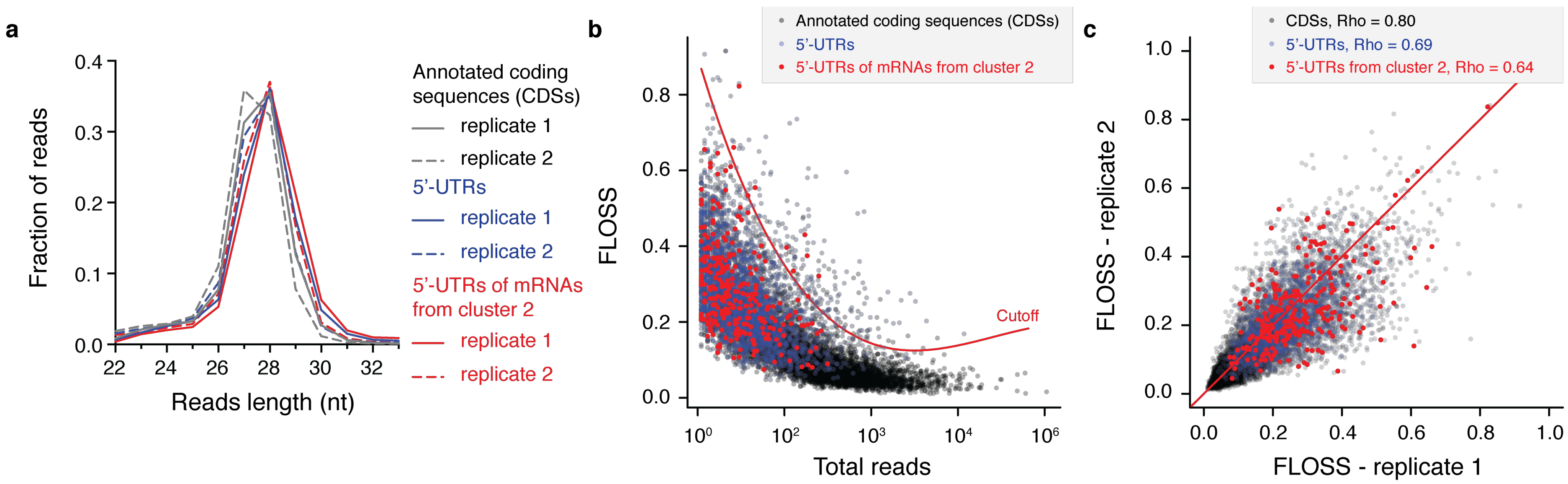

Fig. S2 Classification of 5' UTRs using the FLOSS. a) Fragment length distribution of RPFs mapping to CDSs compared to RPFs mapping 5'-UTRs or the 5'-UTRs of mRNAs from cluster 2 (as defined in **Fig. 1d**). **b**) Disagreement between the distributions was quantified using the FLOSS. Plotting FLOSS in function of the number of reads within the regions of interest and defining a cutoff using Tukey's method shows that 95.2 % of all 5'-UTRs (blue) and 96.5 % of 5-UTRs from mRNAs of cluster 2 (red) display reads that are true ribosome footprints rather than nonribosomal contamination. **c**) The FLOSS pipeline was found reproducible as shown by the correlations between values obtained from duplicates.

Principal component analysis (PCA) and Statistical Modelling

Features collection. 5'-UTR sequences using annotation from the version 26 of the human transcriptome from Gencode were recovered. These sequences were used to calculate the quantitative parameters used to describe the different mRNA features discussed in this manuscript. A complete and comprehensive list of these features is reported in the **Table S1**.

Principal component analysis (PCA). A principal component analysis was used to select the sets of transcripts displaying clear signature of rG4 structure in their 5'-UTR. The PCA was performed using the 'factoextra' package in the *R* environment. We selected a subset of the mRNA features that best describe the variability in mRNA features in our dataset by assessing their variances in term of eigenvalues. The first two dimensions of the PCA reported in this manuscript described 50.5 % of the variability of our dataset and used the different features reported in the **Fig. S3**. It is noteworthy that the second dimension separates the features describing dsRNA and rG4 structures.

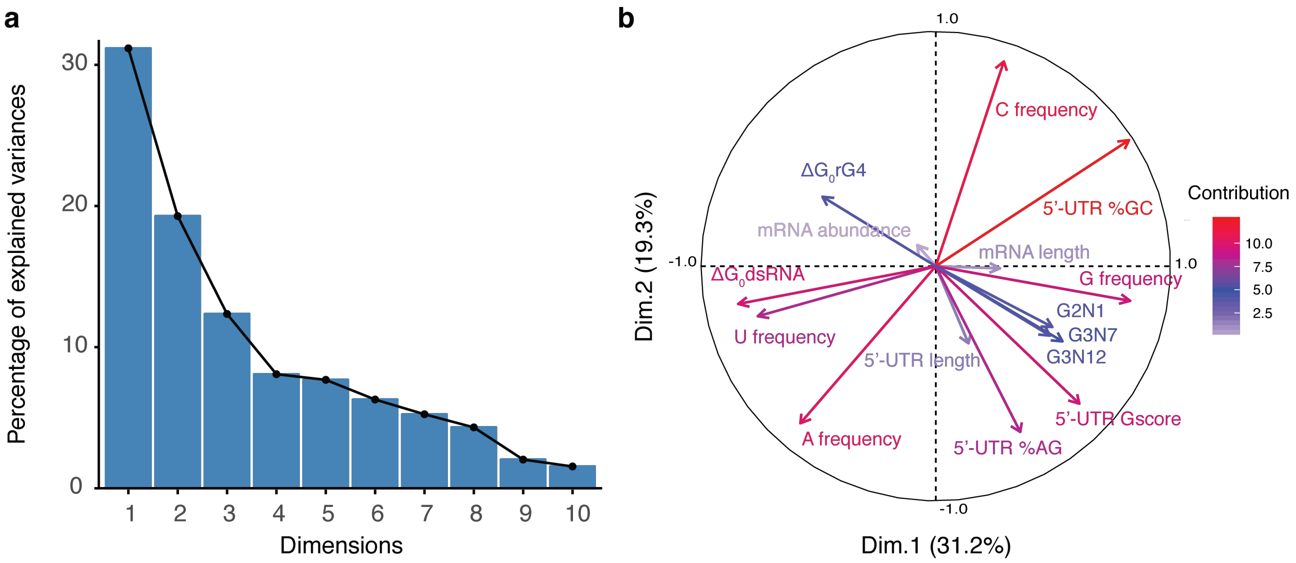

Fig. S3 Principal component analysis. a) Scree plot and **(b)** projection of eigenvectors into the plan of the first two dimensions.

mRNA subsets. We used the PCA to select the subset of transcripts that is characterised by discrete rG4 predicted structures marking their 5'-UTR. This set of transcripts, referred

to as ‘rG4-containing transcripts’, were defined by Dim.1 ≥ 0 and Dim.2 ≤ 0 and consisted of 1,841 transcripts. We also considered all the transcripts expressed in HeLa cells, referred to as ‘all transcripts’, with fully annotated 5’-UTR (with a length ≥ 10 nt) and 3’-UTR. This sets represents 8,024 transcripts.

Features selection. To identify which features explain the highest amount of variation in ribosome distribution (RPFdist), we used a 10-fold cross validation (CV) scheme to select a subset of features with good predictive power. To this end, each feature (see **Table S1**) value was centred and scaled, *i.e.* calculated as z-score. To penalize for model complexity, predictor selection was performed using the LASSO (least absolute shrinkage and selection operator) procedure (‘glmnet’ method from the *R* ‘caret’ package) optimising penalty parameters over the internal cross validation steps. The procedure (using final alpha and lambda parameters of 0.05 and 1 respectively) selected 32 predictors with good predictive power. It is noteworthy that PRTE and TOP-like elements were discarded at this stage. We then assessed the correlations (using a threshold of |correlation| ≤ 0.85) and linear dependencies (using QR decomposition) in between the selected predictors, and found that all selected predictors were independent.

Model selection. We then used the selected list of predictors to build regression models predicting ribosome distribution on both transcript data sets (‘all transcripts’ and ‘rG4-containing transcripts’). To this end, each feature was centred and scaled, both data sets were randomly portioned into 4 sets: 70% of the sets were used for training and the remaining 30% were equally portioned providing 3 testing sets. The training sets were used to select models using a gradient boosting approach (‘gbm’ model from the caret package). Models were optimised by tuning the gradient boosting parameters over a 10-fold cross validation scheme. The optimised parameters were the number of iterations, the complexity of the tree, the learning rate and the minimum number of training set samples in a node to commence splitting. To assess the overall performance of the models, we then challenged them against the three test sets (see **Additional File 1: Fig. S5**). Models explaining the highest amount of variation on both the training and test sets were selected. This procedure was used to select models predicting the ribosome distribution (RPFdist) over the two sets of transcripts while considering all predictors or only a subset

of predictors according to their category. Seven categories of predictors were studied independently and each predictor was assigned to one of the category (see **Supplementary Table 1**) describing: mRNA abundance, sequence length, base composition statistics, dsRNA structures, rG4 structures, upstream open reading frames (uORF) or other features (such as known *cis*-regulatory elements of translation initiation). The performance of the best models selected for each set of transcripts and each subset of predictors in reported in the **Table S1**.

Model comparison. To characterise the differences between models (generated using different categories of predictors) and quantify the contribution of each predictor category, we compared their resampling distributions. We generate resampling distributions (10 CV repeated 10 times) for each model, using the 'resamples' function of the 'caret' package. Since models were fit on the same versions of the training data, the differences between the resampling distributions reflect the differences between model performances rather that the correlations that may exist within-resamples. Differences between model performances were assessed using a non-parametric Kolmogorov-Smirnov test and are reported **Fig. 2f**.

Table S1. Determinants of translation initiation and efficiency. This table reports the name and the description of mRNA features, organised by categories, which were considered as determinants of translation efficiency and ribosome distribution. The identification of these features in mRNA sequences was performed using custom scripts ran with either Python or *R* on sequences recovered from the version 26 of the human transcriptome from Gencode. The last column of the table reports the prediction performance (mean and s.d. over 10 resampling steps) of models considering only one category of features. When considering all predictors, the best models performed at 0.48 ± 0.03 R^2 and 0.56 ± 0.08 R^2 when considering 'all transcripts' or only the 'rG4-containing transcripts' respectively.

Category	Name	Description	Best model (R^2) Mean ± s.d.
**mRNA abundance**	*mRNA abundance*	mRNA abundance was assessed using the matched RNA-seq experiments performed in parallel of ribosome profiling. mRNA abundance was expressed as the log10 of total RNA signal over the CDSs in TPM.	All transcripts: 0.04 ± 0.02<br>rG4-containing transcripts: 0.05 ± 0.03
**Length**	*mRNA length*<br>*5'-UTR length*<br>*5'-UTR relative length*	Log10 of the length of mRNA sequences comprising 5'-UTR, CDS and 3'-UTR.<br>Log10 of the length of 5'-UTR sequences.<br>Ratio between the length of 5'-UTR sequences over the length of the corresponding CDS.	All transcripts: 0.42 ± 0.03<br>rG4-containing transcripts: 0.37 ± 0.10
**Base Composition**	*Base frequency*<br>*GC content*<br>*Purine skew*	The frequency of A, C, G and U nucleotides in 5'-UTR sequences. The frequency of nucleotide N was defined as N/(A+C+G+U)<br>GC-richness of 5'-UTR expressed as (G+C)/(A+C+G+U). GC-richness may also be an indication of secondary structures.<br>Bias in purine frequency of 5'-UTR sequences expressed as (A+G)/(A+C+G+U).	All transcripts: 0.13 ± 0.02<br>rG4-containing transcripts: 0.15 ± 0.04
**dsRNA structures**	ΔG^0_{dsRNA}<br>*Length-normalised* ΔG^0_{dsRNA}<br>*dsRNA cap structures*	5'-UTR minimum free energy (MFE) predicted by RNAfold 2.2.10 considering only dsRNA structures.<br>dsRNA 5'-UTR MFE normalised to 5'-UTR length.<br>dsRNA MFE of sequences comprising the first 30 nucleotides of 5'-UTR.	All transcripts: 0.34 ± 0.04<br>rG4-containing transcripts: 0.28 ± 0.08
**rG4 structures**	ΔG^0_{rG4}	5'-UTR minimum free energy (MFE) predicted by RNAfold 2.2.10. The algorithm was run twice considering dsRNA structures only (ΔG^0_{dsRNA}) and considering rG4 structures in the context of dsRNA structures ($\Delta G^0_{dsRNA+rG4}$). ΔG^0_{rG4} is the difference between both MFE ($\Delta G^0_{rG4} = \Delta G^0_{dsRNA+rG4} - \Delta G^0_{dsRNA}$).	

	Length-normalised ΔG^{0}_{rG4}	rG4 5'-UTR MFE normalised to 5'-UTR length.	
	rG4 cap structures	rG4 MFE of sequences comprising the first 30 nucleotides of 5'-UTR.	All transcripts:<br>0.14 ± 0.03<br><br>rG4-containing transcripts:<br>0.32 ± 0.07
	Gscore	Quantitative estimation of G-richness and G-skewness of 5'-UTR sequences. Gscore calculation is based on the G4Hunter algorithm developed by Bedrat *et al.*[2] Briefly, each position in a sequence is given a score between -4 and 4. To account for G-richness, a single G is given a score of 1, in a GG sequence each G is given a score of 2; in a GGG sequence each G is given a score of 3; and in a sequence of 4 or more Gs each G is given a score of 4. To account for G-skewness, Cs are scored similarly but values are negative. The Gscore is the maximum value obtained while scanning 5'-UTR sequences using a 35 nt window and averaging the score of each nucleotides over the considered window.	
	G3N7 motifs	Number of G3N7 motifs in 5'-UTR sequences, which represent the strict definition of G4 forming sequences according to the Quadparser algorithm introduced by Huppert *et al.*[3] G3N7 motifs are sequences of the form $d(\mathbf{G}_{3+}N_{1-7}\mathbf{G}_{3+}N_{1-7}\mathbf{G}_{3+}N_{1-7}\mathbf{G}_{3+})$, where N is any base.	
	G3N12 motifs	Number of G3N12 motifs, which represent the loose definition of G4 forming sequences.[4] G3N12 motifs are sequences of the form $d(\mathbf{G}_{3+}N_{1-12}\mathbf{G}_{3+}N_{1-12}\mathbf{G}_{3+}N_{1-12}\mathbf{G}_{3+})$, where N is any base.	
	G2N1 motifs	Number of G2N1 motifs, which are enriched in the 5'-UTR of eIF4A-dependent mRNAs[5] and can impede ribosome scanning.[6] G2N1 motifs are sequences of the form $d(\mathbf{G}_{2+}N_{1}\mathbf{G}_{2+}N_{1}\mathbf{G}_{2+}N_{1}\mathbf{G}_{2+})$, where N is any base.	
**uORF**	*Upstream start codons*	Number of in-frame and out-of-frame upstream AUG and non-AUG start codons. The number of triplets (AUG, CUG, GUG and UUG) in annotated 5'-UTRs were considered independently.	All transcripts:<br>0.35 ± 0.02<br><br>rG4-containing transcripts:<br>0.32 ± 0.07
	Upstream open reading frames	Number of potential reading frames upstream the main ORF. The number of sequences in annotated 5'-UTRs with AUG and non-AUG (CUG,GUG and UUG) in frame with a stop codon (UAA, UAG and UGA). The number of AUG and non-AUG uORFs were considered independently.	
**Other**	*Homopolymeric tracts*	Number of A, G, C and U homopolymeric tracts with length superior or equal 5.	
	CERT elements	Number of cytosine-enriched regulator of translation (CERT) elements. CERT motifs are 15 nt long motifs enriched within the 5'-UTR of eIF4E-dependent mRNAs.[7] In this work, CERT motifs were defined as sequences complying to the following regular expression: [CG][CGU][CGU][CG][CGU][C][CGU][C][CA][GU][C][CGU][CGUA][CG][C].	All transcripts:<br>0.11 ± 0.02<br><br>rG4-containing transcripts:<br>0.10 ± 0.06
	PRTE elements	Number of pyrimidine-rich translational element (PRTE) elements. PRTE elements consist of sequences with invariant uridine at position 6 flanked by pyrimidines that are enriched within the 5'-UTR of mTOR-dependent mRNAs.[8] In this work, PRTE motifs were defined as sequences complying to the following regular expression:	

	[CU][CU][CU][CU][CU][U][CU][CU][CU]
TISU elements	Number of Translator Initiator of Short 5'-UTR (TISU) elements. TISU elements are translation and transcription regulatory elements enriched in the 5'-UTR of TATA-less promoter-containing protein coding genes.[9] In this work, TISU motifs were defined as sequences complying to the following regular expression: [CG][A][A][CG][A][U][G][G][C][G][G][C].
TOP-like elements	Presence of terminal oligopyrimidine tract (TOP)-like elements. TOP-like elements are short pyrimidine-rich elements found at the 5' extremity of mTORC1-dependent mRNAs.[10] In this work, TOP elements containing mRNAs were defined as mRNAs with 5'-UTR starting with sequences complying to the following regular expression: [C][TC][TC][TC][TC].

Table S2. Oligonucleotides used in this study.

Name	Sequence (5' to 3')
**Oligonucleotides for biophysics**	
g4-EED	GGGAGGGCGGCGGGAAAAGGGCAAGACGGGAGUUGGGGAAGGG
g4-EED mutated	GAGAGAGCGGCGAGAAAAGAGCAAGACGAGAGUUGAAGAAGAG
g4-DDX23	GGGCGGUUCAGACUCAGGGUGUAGAGAUGGGG
g4-DDX23 mutated	GAGCGAUUCAGACUCAGAGUGUAGAGAUGAAG
**Oligonucleotides for reporter assay** (rG4 forming sequence in red, uORF start and stop codon in blue, eGFP start codon in bold)	
PCR forward primer	CATCCTCTAGACTGCCGGATCTCG
PCR reverse primer	GAACAGCTCCTCGCCCTTGCTG
Sequencing primer 1	GGATACACGCCGCCCACGTG
Sequencing primer 2	TAGGTGGCATCGCCCTCGCCCT
uORF + rG4	CATCCTCTAGACTGCCGGATCTCGAGTAACTAACTAATTTATGG GTGAGGCCGCGTTGGGCGGTTCAGACTCAGGGTGTAGAGAAGGG GTTTGATTACC**ATG**GGCGAATTCAGCAAGGGCGAGGAGCTGTTC
uORF + rG4 mutated	CATCCTCTAGACTGCCGGATCTCGAGTAACTAACTAATTTATGG GTGAGGCCGCGTTGAGCGATTCAGACTCAGAGTGTAGAGAAGAA GTTTGATTACC**ATG**GGCGAATTCAGCAAGGGCGAGGAGCTGTTC
Δ uORF + rG4	CATCCTCTAGACTGCCGGATCTCGAGTAACTAACTAATTTTTTG GTGAGGCCGCGTTGGGCGGTTCAGACTCAGGGTGTAGAGAAGGG GTTTGATTACC**ATG**GGCGAATTCAGCAAGGGCGAGGAGCTGTTC
Δ uORF + rG4 mutated	CATCCTCTAGACTGCCGGATCTCGAGTAACTAACTAATTTTTTG GTGAGGCCGCGTTGAGCGATTCAGACTCAGAGTGTAGAGAAGAA GTTTGATTACC**ATG**GGCGAATTCAGCAAGGGCGAGGAGCTGTTC

Additional references.

1. Ingolia, N. T., Ghaemmaghami, S., Newman, J. R. S. & Weissman, J. S. Genome-Wide Analysis in Vivo of Translation with Nucleotide Resolution Using Ribosome Profiling. *Science* **324,** 218–224 (2009).

2. Ingolia, N. T., Brar, G. A., Stern-Ginossar, N., Harris, M. S., Talhouarne, G. J. S., Jackson, S. E., Wills, M. R. & Weissman, J. S. Ribosome Profiling Reveals Pervasive Translation Outside of Annotated Protein-Coding Genes. *Cell Rep.* **8,** 1365–1379 (2014).

3. Bedrat, A., Lacroix, L. & Mergny, J. Re-evaluation of G-quadruplex propensity with G4Hunter. *Nucleic Acids Res.* **44,** 1746–1759 (2016).

4. Huppert, J. L. & Balasubramanian, S. Prevalence of quadruplexes in the human genome. *Nucleic Acids Res.* **33,** 2908–2916 (2005).

5. Kwok, C. K., Marsico, G., Sahakyan, A. B., Chambers, V. S. & Balasubramanian, S. rG4-seq reveals widespread formation of G-quadruplex structures in the human transcriptome. *Nat. Methods* **13,** 841–848 (2016).

6. Wolfe, A. L. *et al.* RNA G-quadruplexes cause eIF4A-dependent oncogene translation in cancer. *Nature* **513,** 65–70 (2014).

7. Murat, P., Zhong, J., Lekieffre, L., Cowieson, N. P., Clancy, J. L., Preiss, T., Balasubramanian, S., Khanna, R. & Tellam, J. G-quadruplexes regulate Epstein-Barr virus-encoded nuclear antigen 1 mRNA translation. *Nat. Chem. Biol.* **10,** 358–64 (2014).

8. Truitt, M. L., Conn, C. S., Shi, Z., Seo, Y., Barna, M., Truitt, M. L., Conn, C. S., Shi, Z., Pang, X., Tokuyasu, T. & Coady, A. M. Differential Requirements for eIF4E Dose in Normal Development and Cancer. *Cell* **162,** 59–71 (2015).

9. Hsieh, A. C. *et al.* The translational landscape of mTOR signalling steers cancer initiation and metastasis. *Nature* **485,** 55–61 (2012).

10. Dikstein, R. Transcription and translation in a package deal: The TISU paradigm. *Gene* **491,** 1–4 (2012).

11. Thoreen, C. C., Chantranupong, L., Keys, H. R., Wang, T., Gray, N. S. & Sabatini, D. M. A unifying model for mTORC1-mediated regulation of mRNA translation. *Nature* **486,** 109–113 (2012).
