# Supplementary material for: RNA G-quadruplexes at upstream open reading frames cause DHX36- and DHX9-dependent translation of human mRNAs
Source: Genome Biol. 2018 Dec 27;19:229. doi: 10.1186/s13059-018-1602-2 (PMC6307142; doi:10.1186/s13059-018-1602-2)
Supplement: Supplementary file 8 — Review history. (DOCX 47 kb) [file 13059_2018_1602_MOESM8_ESM.docx]

Review History

**First round of review**

**Reviewer 1 wished to have his/her comments unpublished. This reviewer’s comments and the authors’ responses to these comments have been left out of this document.**

**Reviewer 2**

**Are the methods appropriate to the aims of the study, are they well described, and are necessary controls included?**

Yes

**Are the conclusions adequately supported by the data shown?**

In general yes. There is one feature of the model at the end of the paper that does seem to be consistent with the data, as explained in point 1 of my comments to the authors.

**Are sufficient details provided to allow replication and comparison with related analyses that may have been performed?**

Yes

**Does the work represent a significant advance over previously published studies? Please explain.**

Yes -- as described in comments to authors, the work presents a global survey of correlations between translation efficiency and the potential for 5'UTR structure, including G4s, and suggests that DEAH-box helicases DHX36 and DHX9 are involved in regulating 5'UTR structure, presumably by using ATP to disrupt structure.

**Is the paper of broad interest to others in the field, or of outstanding interest to a broad audience of biologists?**

Yes: I think the work will be of broad interest to those studying translation, including the processes of initiation, scanning, and uORF translation. It will also be of interest to those studying RNA structure and the roles of helices in manipulating RNA structure.

**Comments to author**

This manuscript describes a series of genomic experiments that identify links between translation and the potential for 5'-UTR structure, including G4s, and the impact of key RNA helicases on this translation. There are several important findings. First, RNAs with the potential for stable structure, including G4s, tend to be translated less efficiently. Intriguingly, this set of mRNAs includes genes involved in growth and cancer. Second, these inefficiently translated genes frequently have ribosomes bound within the 5'-UTR that are translating uORF sequences. Third, DHX36 and DHX9 are enriched in polysomes, suggesting a function on translated RNAs, and depletion of DHX36 or DHX9 renders translation of mRNAs with the potential for structure in the 5'-UTR even less efficient. These mRNAs include a significant fraction with potential for a G4.

The results have broad implications and will be appreciated by researchers interested in rG4s, helicases linked to them, and those more broadly interested in translational regulation. Nevertheless, there are several points regarding the presentation of the work that should be improved.

1. In the current form of the manuscript, the model regarding the role of the helicases on uORF translation is unclear. The data indicate that the presence of the helicases favors translation of uORFs, apparently at the expense of the genic coding sequence. This seems clear through the Results section. But then at the close of the Discussion, one encounters: "Our data support a previously unknown mechanism in which rG4 folding, mediated by the two DEAH-box helicases DHX36 and DHX9, impedes the scanning of the 43S preinitiation complex…" The straightforward read of this sentence is that the helicases contribute to blocking scanning, but both the data in the current paper and the prior knowledge of the role of helicases indicate that the opposite is true. Helicases disrupt the G4s, which promotes scanning of the ribosome. This could be just ambiguity about the meaning of 'mediated', but then the next sentence seems to confuse things more and along the same lines: "Because 5'-UTR translation is crucial to stress response and cancerogenesis, our findings suggest rG4s associated helicases as new targets for therapeutic intervention." But again, the helicases disrupt the G4s, decreasing uORF translation, so if the goal of a therapeutic were to block uORF translation, one would want to activate the helicase, not block its function. Disrupting helicase function would seem to be predicted by the model to decrease translation of the coding sequence, and given that the genes with structured 5'UTR sequences include oncogenes, perhaps this would be a basis for suggesting these helicases as a therapeutic rather than linking the helicases to a positive role for uORF translation in promoting cancer.

2. DHX36 and DHX9 are referred to as helicases that disrupt G4s specifically (e.g. p. 4, "Here we…dissect the translational output of two rG4-specialized helicases", p. 16, "Characterising how two rG4-specific helicases modulate translation efficiency", p. 16 "Our new data shows that depletion of rG4 processing enzymes, that bind the rG4 structural motif in a cellular environment…". In reality, the knowledge about the extent to which the two helicases are directed toward G4s is not at all equal. DHX36 has been shown to bind with pM affinity to G4s with the involvement of a specific G4 binding domain in the protein (Lattman et al, NAR 2010; Heddi et al, PNAS 2015), and there are recent detailed studies on interactions of DHX36 with G4s from structural and mechanistic perspectives (Chen et al, Structure 2018; Yangyuoru et al, JBC 2018). In contrast, DHX9 has ancillary domains that bind dsRNA and is not known to possess a domain that recognizes G4s specifically, to my knowledge there is only one paper that tested whether DHX9 disrupts G4s (Chakraborty and Grosse, DNA Repair 2011, as cited in the current manuscript), and it was not probed in that work whether DHX9 is specific in any way for G4s. It is intriguing that the current manuscript shows that DHX36 and DHX9 are present on many of the same mRNAs, and it is a reasonable proposition that DHX9 functions in part on G4s. However, it is misleading to suggest that these results fit in with an existing body of knowledge on the molecular properties of DHX9 toward G4s.

3. Along with the analysis of the relationship between the potential for 5'UTR structure and translation efficiency shown in Fig. 1b and c, it would be helpful to include plots displaying, for all mRNAs analyzed, the translation efficiency vs predicted stability of G4s (analogous to panel b) and hairpins (analogous to panel c), presumably as Supplementary material.

4. p. 4, the conclusion that eIF4A disrupts G4s in 5' UTRs has been challenged by a recent paper in which it was shown that many of the sequences previously inferred to form G4s in vivo are actually more stable as hairpins (Waldron et al, NAR 2018). This recent work should also be noted here.

5. It would be useful to know the extent to which the mRNAs pulled down with DHX9 overlap with those pulled down by DHX36 in the previous work (Lattman et al, NAR 2011).

6. p. 16 "It was recently proposed that rG4s are globally unfolded in eukaryotic cells, and that the regulatory role of rG4s in 5'-UTRs might result from the stable association of RNA-binding proteins that maintain the rG4 regions in the unfolded state [56]." The work in ref. 56 (Guo et al, Science 2016) did more than just propose that rG4s are globally unfolded - the authors used footprinting methods to measure rG4 formation across mRNAs in vivo and found no evidence for rG4 formation beyond the limit of detection. As noted by the authors in that work, it would be possible that very low levels of G4s were present and escaped detection. Nevertheless, this set of results would seem to place important constraints on viable models for rG4s in mRNAs, and it is important to keep in mind that the work in the current manuscript does not probe directly for rG4 formation, but rather infers rG4 formation based on sequence potential, the link to decreased translation, and the co-localization of DHX36 and DHX9.

**Reviewer 3**

**Are the methods appropriate to the aims of the study, are they well described, and are necessary controls included?**
Yes

**Are the conclusions adequately supported by the data shown?**

Yes

**Are sufficient details provided to allow replication and comparison with related analyses that may have been performed?**

Yes

**Does the work represent a significant advance over previously published studies? Please explain.**

The work reveals that mechanism underlying translational regulation by rG4s.

**Is the paper of broad interest to others in the field, or of outstanding interest to a broad audience of biologists?**

Yes: The work bring new knowledge in the field of translation and cancer gene expression regulation by specific RNA structures

**Comments to author**

The presence of stable rG4s is often associated to translational repression. Murat and collaborators provide novel mechanistic insights into the RG4-dependent inhibitory mechanism by showing that this involves the translation of upstream open reading frames regulated by rG4-bound helicases.

Major points:

-To provide more convincing evidence of the impact of rG4s on uORF/CDS translation, the authors should validate that the G-rich sequences predicted to form rG4s do fold into rG4 structures in vitro/cellulo. This is important since recent studies show i) differences between rG4 predictions and experimental validation in vitro and in cellulo (ex Guo, 2016), ii) GGC-rich motifs (as those in fig. S7) in full-length 5'UTRs could form stem-loop secondary structures rather than rG4s (Waldron, 2018).

-It would greatly strengthen the study if the authors validate the model proposed in Fig.6E using reporter constructs that bear selected 5'UTRs derived from their analyses.

-The distribution of helicases in polysomes should be controlled using puromycin or EDTA.

Minor points:

-	Appropriateness of ref 19: the rG4 sequence inserted in the 5'UTR of the luciferase reporter mRNA derives from the coding sequence.

-	The observation that DHX36 has been found dispensable for the unfolding of endogenous RG4 should be discussed.

-	Fig. 3C: Clarify "Rep" in the legend.

**Authors Response**

**Point-by-point responses to the reviewers’ comments:**

Reviewer #2:

This manuscript describes a series of genomic experiments that identify links between translation and the potential for 5'-UTR structure, including G4s, and the impact of key RNA helicases on this translation. There are several important findings. First, RNAs with the potential for stable structure, including G4s, tend to be translated less efficiently. Intriguingly, this set of mRNAs includes genes involved in growth and cancer. Second, these inefficiently translated genes frequently have ribosomes bound within the 5'-UTR that are translating uORF sequences. Third, DHX36 and DHX9 are enriched in polysomes, suggesting a function on translated RNAs, and depletion of DHX36 or DHX9 renders translation of mRNAs with the potential for structure in the 5'-UTR even less efficient. These mRNAs include a significant fraction with potential for a G4.

The results have broad implications and will be appreciated by researchers interested in rG4s, helicases linked to them, and those more broadly interested in translational regulation. Nevertheless, there are several points regarding the presentation of the work that should be improved.

1. In the current form of the manuscript, the model regarding the role of the helicases on uORF translation is unclear. The data indicate that the presence of the helicases favors translation of uORFs, apparently at the expense of the genic coding sequence. This seems clear through the Results section. But then at the close of the Discussion, one encounters: "Our data support a previously unknown mechanism in which rG4 folding, mediated by the two DEAH-box helicases DHX36 and DHX9, impedes the scanning of the 43S preinitiation complex…" The straightforward read of this sentence is that the helicases contribute to blocking scanning, but both the data in the current paper and the prior knowledge of the role of helicases indicate that the opposite is true. Helicases disrupt the G4s, which promotes scanning of the ribosome. This could be just ambiguity about the meaning of 'mediated', but then the next sentence seems to confuse things more and along the same lines: "Because 5'-UTR translation is crucial to stress response and cancerogenesis, our findings suggest rG4s associated helicases as new targets for therapeutic intervention." But again, the helicases disrupt the G4s, decreasing uORF translation, so if the goal of a therapeutic were to block uORF translation, one would want to activate the helicase, not block its function. Disrupting helicase function would seem to be predicted by the model to decrease translation of the coding sequence, and given that the genes with structured 5'UTR sequences include oncogenes, perhaps this would be a basis for suggesting these helicases as a therapeutic rather than linking the helicases to a positive role for uORF translation in promoting cancer.

*These sentences from the conclusion section were indeed equivocal and have now been modified to read as follows: “Our data support a previously unknown mechanism in which rG4 folding, controlled by the two DEAH-box helicases DHX36 and DHX9, impedes the scanning of the 43S preinitiation complex, promotes 80S ribosome formation within 5’-UTRs and consequently represses the translation of transcripts involved in key biological pathways. Because of the enrichment of transcripts with structured 5’-UTRs in cancer pathways and the overexpression of rG4-unwinding helicases in cancer tissues, our findings suggest rG4s associated helicases as new targets for therapeutic intervention.” (p19 L9-17).*

2. DHX36 and DHX9 are referred to as helicases that disrupt G4s specifically (e.g. p. 4, "Here we…dissect the translational output of two rG4-specialized helicases", p. 16, "Characterising how two rG4-specific helicases modulate translation efficiency", p. 16 "Our new data shows that depletion of rG4 processing enzymes, that bind the rG4 structural motif in a cellular environment…". In reality, the knowledge about the extent to which the two helicases are directed toward G4s is not at all equal. DHX36 has been shown to bind with pM affinity to G4s with the involvement of a specific G4 binding domain in the protein (Lattman et al, NAR 2010; Heddi et al, PNAS 2015), and there are recent detailed studies on interactions of DHX36 with G4s from structural and mechanistic perspectives (Chen et al, Structure 2018; Yangyuoru et al, JBC 2018). In contrast, DHX9 has ancillary domains that bind dsRNA and is not known to possess a domain that recognizes G4s specifically, to my knowledge there is only one paper that tested whether DHX9 disrupts G4s (Chakraborty and Grosse, DNA Repair 2011, as cited in the current manuscript), and it was not probed in that work whether DHX9 is specific in any way for G4s. It is intriguing that the current manuscript shows that DHX36 and DHX9 are present on many of the same mRNAs, and it is a reasonable proposition that DHX9 functions in part on G4s. However, it is misleading to suggest that these results fit in with an existing body of knowledge on the molecular properties of DHX9 toward G4s.

*According to the existing literature, DHX9 can indeed interact with other structures in addition to rG4s and is in that respect different from DHX36. We have changed some of the text to reflect this point: in the introduction the multifunctional role of DHX9 has been emphasized (p4 L3-4, p4 L9-12) and we are now describing DHX9 as an “rG4-unwinding helicase” rather than a “rG4-specific helicase” throughout the manuscript (p1 L33, p4 L30, p10 L5, p18 L17).*

3. Along with the analysis of the relationship between the potential for 5'UTR structure and translation efficiency shown in Fig. 1b and c, it would be helpful to include plots displaying, for all mRNAs analyzed, the translation efficiency vs predicted stability of G4s (analogous to panel b) and hairpins (analogous to panel c), presumably as Supplementary material.

*We have now added in supplementary information (Additional File 1) a figure (Fig S1c) reporting plots displaying the translation efficiency versus the predicted stability of rG4 and dsRNA structures of the 5’-UTRs of all mRNAs analyzed.*

4. p. 4, the conclusion that eIF4A disrupts G4s in 5' UTRs has been challenged by a recent paper in which it was shown that many of the sequences previously inferred to form G4s in vivo are actually more stable as hairpins (Waldron et al, NAR 2018). This recent work should also be noted here.

*The report from Waldron et al. was published during the latest stage of the preparation of our manuscript and we failed to cite and discuss it. This article is now mentioned in the revised version of the manuscript (p4 L23-26).*

5. It would be useful to know the extent to which the mRNAs pulled down with DHX9 overlap with those pulled down by DHX36 in the previous work (Lattman et al, NAR 2011).

*Lattman et al. reported a list of transcripts bound by DHX36 identified by RIP-chip coupled to microarray analysis. Out of the 100 mRNAs reported to be bound by DHX36, 67 were found in our dataset (transcripts consistently and reproducibly expressed in HeLa cells as assessed by RNA-seq). Only 6 out of these 67 transcripts are also found among the 2,667 transcripts bound by DHX9 identified by iCLIP. Unfortunately the low number of transcripts identified by Lattman et al. does not allow a confident assessment of whether the overlap of the two datasets is significant.*

6. p. 16 "It was recently proposed that rG4s are globally unfolded in eukaryotic cells, and that the regulatory role of rG4s in 5'-UTRs might result from the stable association of RNA-binding proteins that maintain the rG4 regions in the unfolded state [56]." The work in ref. 56 (Guo et al, Science 2016) did more than just propose that rG4s are globally unfolded - the authors used footprinting methods to measure rG4 formation across mRNAs in vivo and found no evidence for rG4 formation beyond the limit of detection. As noted by the authors in that work, it would be possible that very low levels of G4s were present and escaped detection. Nevertheless, this set of results would seem to place important constraints on viable models for rG4s in mRNAs, and it is important to keep in mind that the work in the current manuscript does not probe directly for rG4 formation, but rather infers rG4 formation based on sequence potential, the link to decreased translation, and the co-localization of DHX36 and DHX9.

*We have now extended the discussion about the work from Guo et al. in the revised version of the manuscript (p18 L19 – p19 L2). As stated by this reviewer, in this work, we infer rG4 formation from ribosome pausing events at rG4s and changes in translation upon depletion of rG4-unwinding helicases. We found that transient rG4 folding, that cannot be captured by traditional footprinting assays such as the one used by Guo et al., can have a profound effect on translation. These observations highlight the need to improve or develop methods to probe the dynamic folding of RNA structures in vivo in order to better understand the impact of rG4 on RNA biology.*

Reviewer #3:

The presence of stable rG4s is often associated to translational repression. Murat and collaborators provide novel mechanistic insights into the RG4-dependent inhibitory mechanism by showing that this involves the translation of upstream open reading frames regulated by rG4-bound helicases.

Major points:

1. To provide more convincing evidence of the impact of rG4s on uORF/CDS translation, the authors should validate that the G-rich sequences predicted to form rG4s do fold into rG4 structures in vitro/cellulo. This is important since recent studies show i) differences between rG4 predictions and experimental validation in vitro and in cellulo (ex Guo, 2016), ii) GGC-rich motifs (as those in fig. S7) in full-length 5'UTRs could form stem-loop secondary structures rather than rG4s (Waldron, 2018).

*This reviewer suggests that probing for the folding of some identified rG4 structures in vitro/ vivo will provide more evidences of the impact of rG4s on uORF/CDS translation. In order to provide additional evidences supporting that the structure rather than the G-richness of quadruplex-forming sequences impact translation, we have analysed and characterised using Circular Dichroism spectroscopy the folding of two 5’-UTR rG4 motifs highlighted in the figures of the manuscript (rG4 within the 5’-UTR of EED (Fig 1e) and DDX23 (Fig 5f)). We found that both sequences fold into K+ dependent structures and that G-to-A mutations of key G residues prevent folding, which is characteristic of rG4 formation. CD spectroscopic characterisation of both rG4 motifs is reported in Figure S2 and S12. These experiments are mentioned in the figure legends (p43 L15-16 and p47 L8-10). Description of the CD spectroscopy experiments is reported in the Method section (p33 L4-18). While probing the folding of rG4, and RNA structures in general, in living cells is an active and challenging field of research in need of further innovation (see also response to point 6 of referee 2), we consider that this is out the scope of the current article.*

2. It would greatly strengthen the study if the authors validate the model proposed in Fig.6E using reporter constructs that bear selected 5'UTRs derived from their analyses.

*Reviewers 1 and 3 have suggested that further validation of our model proposed in Fig 6e would strengthen our study. The essence of our proposed model is that rG4 folding shifts translation from annotated coding regions of mRNAs to short upstream open reading frames in the 5’-UTR. The type of experimental evidence we have provided in the current version of the manuscript, i.e. DHX36- and DHX9-dependent in-frame-periodic distribution of ribosomes within rG4-containing uORFs, is in line with the state-of-the-art methodology and evidence commonly used (and cited) in the field to indicate the translation of small ORFs located in untranslated regions of canonical mRNAs or on transcripts annotated as non-coding (for example see Ruiz-Orera et al. eLife, 2014, 3, e03523 or Bazzini et al. The EMBO Journal, 2014, 33, 981). The reason being there are practical limitations in providing unambiguous and direct evidence for uORF translation. Monitoring the synthesis of short open reading frame-encoded peptides is very challenging due to the cellular instability of short peptides and the lack of available methods for detection. These points have been discussed in recent papers (e.g. Couso et al. Nature Reviews Mol Cell Biol, 2017, 18, 575-589). The use of state-of-the-art peptidomic approaches have only allowed the identification of just a few dozens of short ORF-encoded polypeptides in human cells (e.g. see Slavoff et al. Nature Chem Biol, 2013, 9, 59-64 or Sendoel et al. Nature 2017, 541, 494-499). Therefore, using mass spectrometry to directly detect short peptides is highly specialised and also in need of further technological innovation. This cannot be easily done using biochemical methods for protein detection and isolation because standard methods fail to detect peptides below 10 kDa, which escapes a typical gel or filter. Conventional reporter assays expressing bioluminescent or fluorescent proteins are inadequate because peptides encoded by short uORFs would be non-fluorescent and still be very challenging to detect. The invention of some form of suitable reporter assay that could detect the products of uORF translation would in itself be a major contribution to the field and is beyond the scope of the present study.*

3. The distribution of helicases in polysomes should be controlled using puromycin or EDTA.

*We performed polysome profiling coupled to mass spectrometry experiments to identify helicases associated with polysomes that could contribute to the translation of rG4-containing mRNAs. We introduced a molecular weight cut-off of the proteins analysed to decrease noise and avoid saturation of the mass analysis by peptides originating from highly abundant ribonucleoproteins (hnRNPA1 Mw ~ 40kDa, hnRNPF Mw ~ 50kDa) and ribosomal proteins (RPS2 and RPS6 Mw ~ 30 kDa, associated kinases S6K1 Mw ~ 75 kDa). We set a cut-off at Mw = 100 kDa due to our interest in helicases (DEAD-box helicases Mw ~ 100 – 130 kDa, Ski2-like helicases Mw = 120 – 150 kDa, DEAH-box helicases Mw ~ 130 – 150 kDa) and associated complexes. We have added a comment in the Method section of our revised manuscript (p27 L32 – p28 L2) to clarify this point.*

*The physical association of DHX9 and DHX36 with actively translating polyribosomes (polysomes) has been previously demonstrated in two independent reports (Hartman et al. Nat Struct & Mol Biol 2006, 13, 509-516 and Thandapani et al. eLife 2015, 4, e06234) showing that the two helicases does not co-sediment with mRNPs other than polysomes by showing that puromycin and/or EDTA treatments (both of which cause dissociation of polysomes) lead to the redistribution of the helicases towards the lighter fractions. This supports that these helicases are not incidentally co-sedimenting with polysomes, but rather that they are interacting with polysomes. The main point of our analysis (Figure 3b of our manuscript) was actually to assess differences in the distribution of the RHA-like helicases in the polysomal fractions to select candidate helicases involved in the regulation of the translation of rG4-containing mRNAs. We have rephrased this paragraph in the revised version of the manuscript (p10 L16-32) to clarify this point.*

Minor points:

4. Appropriateness of ref 19: the rG4 sequence inserted in the 5'UTR of the luciferase reporter mRNA derives from the coding sequence.

*This is has now been specified (p3 L19).*

5. The observation that DHX36 has been found dispensable for the unfolding of endogenous RG4 should be discussed.

*Please, refer to point 6 of the answers to reviewer #2.*

6. Fig. 3C: Clarify "Rep" in the legend.

*The figure legend has been amended (p45 L16-17).*

**Second round of review**

**Reviewer 1**

**Reviewer 2**

The authors have adequately addressed all of the points from my earlier review. In my opinion the multiple lines of evidence used by the authors strongly suggest a role of rG4s in suppressing gene translation and promoting translation of uORFs, and a role for DHX36 and DHX9 in reversing these effects, presumably by disrupting the rG4s.

**Reviewer 3**

The authors have provided a strong response to the reviewers' concerns. However, as suggested by reviewer 1 and myself, it is important to validate the proposed mechanism using reporters with 5'UTRs containing uORFs (with AUG wt/mut) +/-G4 (wt/mut) subcloned upstream of a luciferase coding sequence. To ensure that translation is initiated at the uORF AUG the luciferase constructs can also be modified such that the AUGs of the uORFs are in-frame with the luciferase start codon. These experiments are routinely done to demonstrate that uORFs repress translation of the downstream main ORF. The papers cited by the authors concern the identification of uORFs in non-coding RNAs, not the uORF-mediated translational control of the downstream ORF.

**Reviewer 4 (new statistics reviewer)**

I looked over the use of statistics in the manuscript -- overall the authors chose reasonable tests and approaches. Genomic data was analyzed by established methods, and the use of non-parametric statistics (Mann-Whitney, K-S, and Spearman's rho) is appropriate.

My concerns (linked to specific figures)

1a Is translational efficiency computed by assessing the density of ribosome footprints on CDSes alone? Is the start codon excluded due to known cycloheximide artifacts there?

1f and 1g describe a trend with no statistical criteria. The claim of excess RPF coverage on cluster 2 transcripts with strong rG4 structure is pretty central to the manuscript — statistics should be tested.

2b the manuscript interprets periodicity but doesn't provide any statistical test -- there are tests for periodicity and one should be used.

In 3f, reporting the Rho when the Q-value < 0.01 is misleading and should be excluded; the overall Rho clearly supports the overall claim, and the Q-value threshold implicitly selects for points where changes are large and correlated.

**Authors Response**

**Point-by-point responses to the reviewers’ comments:**

Reviewer #3:

The authors have provided a strong response to the reviewers' concerns. However, as suggested by reviewer 1 and myself, it is important to validate the proposed mechanism using reporters with 5'UTRs containing uORFs (with AUG wt/mut) +/-G4 (wt/mut) subcloned upstream of a luciferase coding sequence. To ensure that translation is initiated at the uORF AUG the luciferase constructs can also be modified such that the AUGs of the uORFs are in-frame with the luciferase start codon. These experiments are routinely done to demonstrate that uORFs repress translation of the downstream main ORF. The papers cited by the authors concern the identification of uORFs in non-coding RNAs, not the uORF-mediated translational control of the downstream ORF.

*We have now carried out additional experiments to assess the contribution of quadruplexes to the repressive effect of uORFs. Specifically, we have constructed expression vectors in which the translation of a reporter GFP gene is driven by 5’-UTRs containing either a rG4, a short rG4-containing uORF or a mutated rG4/alternative translation initiation site. An assessment of the expression of these constructs showed that (i) rG4 has negligible impact on translation when close to the start codon of the reporter gene, (ii) the presence of a short uORF moderately affect the expression of the reporter gene and (iii) the presence of a rG4 within an uORF stimulate its repressive effect. These observations support that rG4s within uORFs stimulate the recognition of alternative translation initiation sites in a weak context and thwart the translation of the downstream CDS. Moreover, we also report that depleting DHX36 or DHX9 decreases the expression of a reporter gene driven by a rG4-containing uORF but not of a similar reporter gene in which the rG4 has been mutated. Taken together these results do provide additional support for the mechanism proposed in our model. These experiments are reported in Fig 6d-f and discussed in the manuscript (p17 L28 – p19 L7).*

Reviewer #4:

I looked over the use of statistics in the manuscript -- overall the authors chose reasonable tests and approaches. Genomic data was analyzed by established methods, and the use of non-parametric statistics (Mann-Whitney, K-S, and Spearman's rho) is appropriate.

My concerns (linked to specific figures)

1a Is translational efficiency computed by assessing the density of ribosome footprints on CDSes alone? Is the start codon excluded due to known cycloheximide artifacts there?

*As reported in the “Methods” section, translation efficiency (TE) was calculated as the ratio of the total RPF signal (in TPM) over the coding region (CDS) divided by the total RNA signal (in TPM) over the same region. Signal over the start codons was not excluded since we didn’t identify any enrichment/artifact at these sites. The potential effects of cycloheximide pre-treatment near the start codon have been extensively discussed, but recent reports have demonstrated that the observed “5’ ramp” of ribosomes is rather due to codon usage, ribosome drop-off or the fact that elongation is slower during the early phase of translation (see Weinberg et al. Cell Reports, 2016, 14, 1787-1799 for a comprehensive analysis).*

1f and 1g describe a trend with no statistical criteria. The claim of excess RPF coverage on cluster 2 transcripts with strong rG4 structure is pretty central to the manuscript — statistics should be tested.

*In order to assess the statistical relevance of Fig 1f and 1g, we have quantified the RPF coverage within the 5’-UTRs of the different clusters and within the 5’-UTRs of mRNAs from cluster 2 when binned by quadruplex stability. Appropriate statistical test have been used to demonstrate the relevance of these results. These data are reported in Fig S1g-h in Additional File 1.*

2b the manuscript interprets periodicity but doesn't provide any statistical test -- there are tests for periodicity and one should be used.

*We have found difficult to define a null hypothesis for a statistical test on periodicity. Nevertheless the absence of periodicity observed in the signal related to the background and Low ORFscore uORF supports the relevance of our observations (see answer to reviewer #1 point 2). Instead of providing statistical tests, we now report a quantitative analysis of periodicities by the mean of fast Fourier transforms that allow to unambiguously extract periodicities of 41 - 44 nt from the position of quadruplexes within translated uORFs. We believe this new analysis adds further support our hypothesis.*

In 3f, reporting the Rho when the Q-value < 0.01 is misleading and should be excluded; the overall Rho clearly supports the overall claim, and the Q-value threshold implicitly selects for points where changes are large and correlated.

*We have removed correlations for data with Q-value < 0.01 as it was indeed misleading.*
